# Research Progress in Rare Earth-Doped Perovskite Manganite Oxide Nanostructures

**DOI:** 10.1186/s11671-019-3243-0

**Published:** 2020-01-13

**Authors:** Weiren Xia, Zhipeng Pei, Kai Leng, Xinhua Zhu

**Affiliations:** National Laboratory of Solid State Microstructures, School of Physics, Nanjing University, Nanjing, 210093 China

**Keywords:** Rare earth-doped perovskite manganite, Nanostructures, Fabrication methods, Structural characterization, Physics properties, Functional applications

## Abstract

Perovskite manganites exhibit a broad range of structural, electronic, and magnetic properties, which are widely investigated since the discovery of the colossal magnetoresistance effect in 1994. As compared to the parent perovskite manganite oxides, rare earth-doped perovskite manganite oxides with a chemical composition of Ln_x_A_1-x_MnO_3_ (where Ln represents rare earth metal elements such as La, Pr, Nd, A is divalent alkaline earth metal elements such as Ca, Sr, Ba) exhibit much diverse electrical properties due to that the rare earth doping leads to a change of valence states of manganese which plays a core role in the transport properties. There is not only the technological importance but also the need to understand the fundamental mechanisms behind the unusual magnetic and transport properties that attract enormous attention. Nowadays, with the rapid development of electronic devices toward integration and miniaturization, the feature sizes of the microelectronic devices based on rare earth-doped perovskite manganite are down-scaled into nanoscale dimensions. At nanoscale, various finite size effects in rare earth-doped perovskite manganite oxide nanostructures will lead to more interesting novel properties of this system. In recent years, much progress has been achieved on the rare earth-doped perovskite manganite oxide nanostructures after considerable experimental and theoretical efforts. This paper gives an overview of the state of art in the studies on the fabrication, structural characterization, physical properties, and functional applications of rare earth-doped perovskite manganite oxide nanostructures. Our review first starts with the short introduction of the research histories and the remarkable discoveries in the rare earth-doped perovskite manganites. In the second part, different methods for fabricating rare earth-doped perovskite manganite oxide nanostructures are summarized. Next, structural characterization and multifunctional properties of the rare earth-doped perovskite manganite oxide nanostructures are in-depth reviewed. In the following, potential applications of rare earth-doped perovskite manganite oxide nanostructures in the fields of magnetic memory devices and magnetic sensors, spintronic devices, solid oxide fuel cells, magnetic refrigeration, biomedicine, and catalysts are highlighted. Finally, this review concludes with some perspectives and challenges for the future researches of rare earth-doped perovskite manganite oxide nanostructures.

## Introduction

Perovskite manganites refer to a family of manganese compounds with a general composition of AMnO_3_, where A = La, Ca, Ba, Sr, Pb, Nd, Pr, which crystallize in the perovskite structure named after the mineral CaTiO_3_. Depending on the composition, they exhibit various magnetic and electric phenomena such as ferromagnetic, antiferromagnetic, charge, and orbital ordering. Thus, these properties have potential applications in the fields of sensors and spintronic devices. The early studies of perovskite manganites began in 1950, first performed by Jonner and Van Santen [1]. They found that the change of proportion of Mn^4+^ by introducing the bivalent alkaline earth metal elements (e.g., Ca, Sr, Ba) with different doping ratio into LaMnO_3_, could lead to the changes in the Curie temperature (namely the *T*_C_) and saturation magnetization. Since then the term of “manganites” was adopted to refer to these compounds containing trivalent as well as tetravalent manganese. One year later, Zener [2] proposed a “double exchange” (DE) mechanism to explain the unusual correlation between magnetism and electrical conduction, which was reported by Jonner and Van Santen. Based on the Zener’s theoretical studies; the DE mechanism was further developed in more detail [3–5]. At the same time, the experimental researches were also carried out.

As compared to the parent perovskite manganite oxides, rare earth-doped perovskite manganite oxides with a chemical composition of Ln_x_A_1-x_MnO_3_ (where Ln represents rare earth metal elements such as La, Pr, Nd, A is divalent alkaline earth metal elements such as Ca, Sr, Ba) exhibit much diverse electrical properties due to that the rare earth doping leads to a change of valence states of manganese which plays a core role in the transport properties. For example, La-doped SrMnO_3_ (La_0.7_Sr_0.3_MnO_3_) is a ferromagnetic (FM) metal, whereas SrMnO_3_ is an antiferromagnetic (AFM) insulator. Wollan and Koe [6]. found a series of rare earth-doped perovskite manganite oxides Ln_x_Ca_1-x_MnO_3_ with the feature of FM and AFM properties depending upon the relative ion manganese content (Mn^3+^ and Mn^4+^). In 1994, Jin et al. [7] first reported on the colossal magnetoresistance (CMR) effect in the perovskite La_0.67_Ca_0.33_MnO_3_ thin films grown on LaAlO_3_ substrates by laser ablation, where a several-tesla magnetic field could induce a 1000-fold change in the resistance of the epitaxial thin film of La_0.67_Ca_0.33_MnO_3_. Since that time, perovskite manganites become the focus of great interest again, both theories and experiments have been further advanced. In 1995, Millis et al. [8] pointed out that the phenomena observed in experimental consequences cannot be accounted by double exchange alone, such as the sharp drop in resistivity just below *T*_C_. Before long, Millis et al. [9] indicated that the essential physics of manganites are dominated by the interplay between electron-phonon coupling arising from the Jahn-Teller effects [10] and double exchange mechanism. Later, this newer theory as well as Jahn-Teller effect were adopted and discussed [11, 12]. In order to explain the novel physical transport properties more reasonably, many theoretical models have been proposed in recent years, such as one-orbital model (that is simple but incomplete) and two-orbital model (that is essential to explain the notorious orbital order tendency in Mn-oxides) [13]. From 1998 to 1999, Dagotto and his collaborators [14, 15] developed a theory of phase separation where phase segregation tendencies appeared in manganites. Gradually, phase separation theory was verified and recognized as the mainstream theory describing the perovskite manganese oxides [16, 17].

Rare earth-doped perovskite manganite oxides belong to the group of highly correlated systems, which display a wide spectrum of novel properties, including CMR effect, metal–insulator (M–I) transition, electronic phase separation (EPS), and complex structural phases in their phase diagrams due to the complex interactions among the spin, charge, orbital, and lattice degrees of freedom. There is not only the technological importance but also the need to understand the fundamental mechanisms behind the unusual magnetic and transport properties that attract enormous attention. Nowadays, with the rapid development of electronic devices towards integration and miniaturization, the feature sizes of the microelectronic devices based on rare earth-doped perovskite manganite are down-scaled into nanoscale dimensions. At nanoscale, various finite size effects in rare earth-doped perovskite manganite oxide nanostructures (e.g., zero-dimensional (0D), one-dimensional (1D), and two-dimensional (2D) nanostructures) will lead to more interesting novel properties of this system. In the past two decades, researches on the rare earth-doped perovskite manganite oxide nanostructures have achieved much progress after considerable experimental and theoretical efforts. In this paper, an overview of the state of art in the rare earth-doped perovskite manganite oxide nanostructures is presented, which covers the fabrication, structural characterization, properties, and functional applications. Due to the tremendous research efforts and the space limitations, it would be impossible to provide a complete overview on all existing topical literature, and therefore we limit ourselves to selected, but the representative results. Wherever possible, the readers are referred to the review articles, books and/or chapters in which selected sub-topics on the rare earth-doped perovskite manganite oxide nanostructures are discussed in full detail. Also, this review article seeks to present the topic not only from the viewpoint of fabrication methods but also tries to motivate the interest in these special compounds from the perspective of structural characterization, physical properties, and functional applications in the fields of microelectronic, magnetic, and spintronic devices, solid oxide fuel cells, magnetic refrigeration, biomedicine, and catalysts. This overview ends with some perspectives and challenges for the future researches of rare earth-doped perovskite manganite oxide nanostructures.

## Synthesis Methods of Rare Earth-Doped Perovskite Manganite Oxide Nanostructures

### Rare Earth-Doped Perovskite Manganite Oxide Nanoparticles

#### Molten Salt Synthesis

Molten salt synthesis (MSS) method is a simple, versatile, and environmental-friendly approach, which is widely used to synthesize high purity and nanoscale inorganic oxides with controllable compositions and morphologies. In this approach, inorganic molten salt is served as the reaction medium to enhance the reaction rate and to reduce the reaction temperature of the reactant oxides [18]. Due to the short diffusion distances and large mobilities of the reactant oxides in the molten salts, the whole solid-state reactions are easily carried out at moderate temperatures (600–800 °C) in a short dwell time (less one hour). Besides the low formation temperature, molten salts also promote to stabilize the specific morphology of the final products. In addition, the morphology of the final products can be well controlled by adjusting the MSS processing parameters (e.g., the types and quantities of the used molten salts, different reactant oxides, annealing temperature and dwell time, and heating/cooling rates) in the MSS process.

In recent years, MSS method has been successfully used to synthesize rare earth-doped perovskite manganite oxide nanoparticles. For example, Luo et al. [19] synthesized La_0.7_Sr_0.3_MnO_3_ (LSMO) powders via MSS route, where stoichiometric La(NO_3_)_3_·6H_2_O, Sr(NO_3_)_2_, and Mn(NO_3_)_2_ were used as starting materials, and KNO_3_ was used as molten salt. By controlling the molar ratio of KNO_3_ and metal nitrates and the reaction temperature, they obtained the LSMO particles with average grain size modulated from 20 to 50 nm. A significant enhanced magnetoresistance was observed in these nanosized LSMO powders, especially at low temperature. Tian et al. [20] developed a facile molten salt synthetic route to synthesize La_1-x_Sr_x_MnO_3_ (*x* = 0, 0.3, 0.5, 0.7) nanoparticles, where the eutectic NaNO_3_–KNO_3_ mixture were used as molten salt and the nitrates of La, Mn, and Sr were used as reagents. The average grain sizes of the La_1-x_Sr_x_MnO_3_ (*x* = 0, 0.3, 0.5, 0.7) particles were about 20, 20, 19, and 25 nm, respectively. Later, by the same method, Tian et al. [21] also synthesized the La_0.67_Sr_0.33_MnO_2.91_ nanoparticles with particle sizes in the range of 20–60 nm. Xia et al. [22] also synthesized single-crystalline La_1-x_Ca_x_MnO_3_ (LCMO with *x* = 0.3 and 0.5) nanoparticles by MSS method, where the eutectic NaNO_3_–KNO_3_ mixture was used as the molten salt. By using NaNO_2_ as molten salt, Kačenka et al. [23] synthesized La_1-x_Sr_x_MnO_3_ (*x* = 0.18–0.37) nanoparticles, which were rather separated as compared with that synthesized by sol-gel route. Similarly, a series of single-phase La_1-x_Sr_x_MnO_3_ (*x* = 0.25–0.47) nanoparticles with an average size of ~ 50 nm were also synthesized [24].

#### Mechanochemical Processing

As an effective, economical, and versatile way to synthesizing ultrafine powders, mechanochemical processing (MCP) makes use of chemical reactions activated mechanically by high-energy ball milling. Muroi et al. [25] carried out the pioneering works on the synthesis of perovskite manganites by MCP, where the starting materials were LaCl_3_, CaCl_2_, MnCl_2_, and Na_2_CO_3_ was used as molten salt. They were mixed in an appropriate ratio via a chemical reaction to form La_0.7_Ca_0.3_MnO_3_ powders with particle sizes in the range of 20 nm–1.0 μm. Following a similar method, Spasojevic et al. [26] synthesized the La_0.7_Ca_0.3_MnO_3_ nanoparticles with an average size of 9 nm by high-energy ball milling in a single-step processing. By mechanical alloying method, Li et al. [27] also synthesized La_2/3_Ca_1/3_MnO_3_ powders with a grain size of ~ 18 nm. In another work, Manh’s group carried out a series of studies to synthesize La_0.7_Ca_0.3_MnO_3_ nanoparticles by reactive milling methods [28–32]. They found that the as-synthesized La_0.7_Ca_0.3_MnO_3_ nanoparticles exhibited super-paramagnetic behavior with a blocking temperature, which was reduced as increasing the milling time from 8 to 16 h [28]. Besides the La_0.7_Ca_0.3_MnO_3_ nanoparticles, La_0.7_Sr_0.3_MnO_3_ nanoparticles were also synthesized by reactive milling methods under different milling times [30, 31]. Recently, La_0.7_Ca_0.3_MnO_3_ nanoparticles with particle size of 21–43 nm were also synthesized by reactive milling and thermal processing methods [32].

### Wet chemical Routes

#### Sol-Gel Process

Sol-gel process is a popular method for the synthesis of multicomponent metal oxides such as perovskite oxide materials. This process involves the formation of a sol by dissolving the metal aloxide, metal-organic, or metal-inorganic salt precursors in a suitable solvent, subsequent drying the gel followed by calcination, and sintering at high temperatures to form perovskite oxide materials.

Ravi et al. [33] used a modified sol-gel method to synthesize LSMO nanoparticles, where oxalic acid was used as chelating agent, oleic acid as surfactant in poly acrylic acid matrix, and metal nitrates as starting materials. The xerogel was heated at 100 °C and dried in atmosphere to obtain powders. And then, these powders were grinded and annealed at temperatures from 500 to 800 °C for 4 h to obtain LSMO nanoparticles with different particle sizes. Similarly, Pr_1/2_Sr_1/2_MnO_3_ [34], La_0.6_Pb_0.4_MnO_3_ [35], Nd_0.5_Sr_0.5_MnO_3_ [36], La_1-x_Ca_x_MnO_3_ [37], Ln_0.67_Sr_0.33_MnO_3_ (Ln = La, Pr and Nd) [38], and Pr-doped La_0.67_Ca_0.33_MnO_3_ nanoparticles [39] were also synthesized by this method. Their particle sizes can be well controlled by the annealing temperatures. Sarkar et al. [40] adopted the sol-gel-based polymeric precursor polyol route to synthesize Pr_0.5_Ca_0.5_MnO_3_ nanoparticles with particle size down to 10 nm. In their work, the polymer ethylene glycol was used to form a close network of metal ions in the precursor solution, which assists the reaction and enables the phase formation at relatively low temperatures.

#### Co-precipitation Method

The co-precipitation process involves the separation of a solid containing various ionic species from a solution phase. It is a very rare situation where a quantitative and simultaneous precipitation of all the cations occurs without segregation of any particular constituents in the precipitates to form a completely mixed-metal precursor. That is resulted from the different solubilities between the various precipitating phases, especially in the case of the solution containing more than one metal ion. Normally, this problem can be modified by introducing the precipitating agents (such as oxalates, tartarates, and citrates) that render the cations insoluble. Dyakonov et al. [41] synthesized (La_0.7_Sr_0.3_)_0.9_Mn_1.1_O_3_ manganite nanoparticles by this method, where a mixture of stoichiometric amounts of high purity Mn_3_O_4_, La_2_O_3_, and SrCO_3_ powders was dissolved in diluted nitric acid. This solution was evaporated and dried, and then fired at 500 °C to decompose the nitrates. The dry remainder was thoroughly ground again and annealed at temperatures from 800 to 950 °C for 20 h in air, and then followed by slow cooling down to room temperature. The resulting product was repeatedly ground, and nanopowders with average particle sizes of 40, 75, and 100 nm were obtained. Pang et al. [42] also synthesized the La_0.7_Sr_0.3_MnO_3_ nanoparticles by a sonication-assisted co-precipitation method. Similarly, La_0.5_Ca_0.5_MnO_3_ nanopowders with different average sizes (13, 18, and 26 nm) were obtained after annealing at 700, 800, and 900 °C, respectively [43]. By using an improved chemical co-precipitation method, Zi et al. [44] synthesized La_0.7_Sr_0.3_MnO_3_ nanoparticles with particle sizes in the range of 50–200 nm.

#### (Microwave-) Hydrothermal Process

Hydrothermal process involves heating an aqueous suspension of insoluble salts in an autoclave at a moderate temperature and pressure so that the crystallization of a desired phase will take place. The hydrothermal synthesis is a powerful method for the preparation of very fine and homogeneous perovskite powders with a narrow size distribution and spherical morphology. Sin et al. [45] reported on the synthesis of single-crystalline La_1-x_Sr_x_MnO_3_ nanoparticles by a hydrothermal route in the presence of surfactant named as cetyltrimethylammonium bromide (CTAB). Analytical grade KMnO_4_, MnCl_2_·4H_2_O, LaCl_3_·7H_2_O, SrCl_2_·6H_2_O were used as starting materials. The chemical reactions were carried out in 10 ml Teflon-lined stainless steel autoclaves, where the added KOH maintained a proper alkalinity. Then, the CTAB powder was mixed with the above solution containing metal ions and agitated vigorously to obtain a homogeneous black solution. The reaction mixture was placed in the autoclaves and heated at 240 °C under the autogenously pressure for 1 day. The obtained product was filtered off and washed with ethanol and deionized water to remove the residual CTAB, potassium ions, and chloride ions. The final product was dried at 80 °C for 2 h to yield a small quantity of black powder. Urban et al. [46] also synthesized single-crystalline La_1-x_Ba_x_MnO_3_ (*x* = 0.3, 0.5, and 0.6) nanocubes with sizes of 50–100 nm. Deng et al. [47] reported the synthesis of La_1-*x*_Sr_*x*_MO_3-*δ*_ (M = Co, Mn; *x* = 0, 0.4) particles by using a modified strategy of citric acid coupled with hydrothermal treatment [48]. They found that Sr-doping led to a decrease in the amount of over stoichiometric oxygen and also caused the Mn^4+^ concentration to be increased, improving the redox ability of the catalysts consequently.

Microwave-hydrothermal (M-H) synthesis is a modified approach by involving the microwave heating techniques during the hydrothermal synthesis procedure. The microwave heating manner can largely increase the reaction and crystallization rate, and enhance fabrication efficiency. Recently, this method has been used to synthesize rare earth-doped perovskite manganite oxide nanostructures. Ifrah et al. [49] reported the microwave-assisted hydrothermal synthesis of La_0.8_Ag_0.2_MnO_3+δ_ nanoparticles, which were homogeneous with a crystallite size of 70 nm. Moreover, the La_0.8_Ag_0.2_MnO_3+δ_ nanoparticles were excellent in methane catalytic combustion. Anwar et al. [50] reported the microwave-assisted hydrothermal synthesis of La_0.67_Sr_0.33_MnO_3_ nanoparticles, which had a rod-like morphology with average crystallite size of 11 nm.

#### Pyrophoric Reaction Process

Pyrophoric reaction process involves thermolysis of aqueous precursor solutions of coordinated metal compounds of organic amines and acids via the formation of mesoporous carbon precursors and their calcination at high temperatures (800 °C). Its principle is to atomistically disperse the complex metal ions in the polymeric network provided by organic coordinating agent, i.e., triethanolamine, during the pyrolysis of excess reagents. During the pyrolysis of the precursor solution, the metal ions or their salts form nanoclusters, which are embedded in the resulting matrix of mesoporous carbon. Slow volatilization of mesoporous carbon in the precursor material through low temperature between 500 and 800 °C air oxidation, aided by the catalytic effect of in situ metal ions, favors the formation of metal-oxide nanocrystals. The advantages of this method in preparing oxide nanoparticles are the high purity of the products, small particles sizes with narrow particle size distribution, good compositional control, and chemical homogeneity of the final products.

Dey et al. [51] obtained La_0.7_Ca_0.3_MnO_3_ nanoparticles with average size of 17 nm via pyrophoric reaction process, where high-purity La_2_O_3_, Mn(CH_3_COO)_2_, and CaCO_3_ were used as starting materials. By the same method, Giri et al. obtained Sm_0.5_Ca_0.5_MnO_3_ and Sm_0.09_Ca_0.91_MnO_3_ nanoparticles [52–55]. These nanoparticles exhibit an exchange bias effect, which can be effectively tuned by the cooling field. Nagabhushana et al. [56] also synthesized the La_1-x_Sr_x_MnO_3+δ_ nanopowders, where lanthanum nitrate La(NO_3_)_3_·6H_2_O, strontium nitrate Sr(NO_2_)_3_·4H_2_O, and manganese nitrate Mn(NO_3_)_2_·4H_2_O were used as oxidizers and oxalyl hydrazine, C_2_H_6_N_4_O_2_ (ODH) as a fuel. Shinde et al. [57] reported on the synthesis of a series of Sr-doped lanthanum manganites by simple solution combustion technique. La_0.6_Sr_0.4_MnO_3_ nanoparticles with different particle sizes were also synthesized by the nitrate-complex auto-ignition method [58].

#### Thermal Decomposition Synthesis

Thermal decomposition synthesis is fast, simple, and cost-effective synthesis route for preparations of metal oxide and complex oxide nanoparticles. Monodisperse magnetic nanocrystals with smaller sizes can essentially be synthesized through the thermal decomposition of organometallic compounds in high-boiling organic solvents containing stabilizing surfactants. In principle, the ratios of the starting reagents including organometallic compounds, surfactant, and solvent are the decisive parameters for the control of the size and morphology of magnetic nanoparticles. The reaction temperature and time as well as the aging period may also be crucial for the precise control of size and morphology [59]. The method is simple and convenient in operation, low in cost and high in direct yield, all volatile components volatilize, and the problem of carbon impurities is solved.

Recently, Huang et al. [60] synthesized the La_0*.*7_Sr_0*.*3_MnO_3_ particles via the thermal decomposition of metal–complexes by using ethylenediaminetetraacetic acid as a complex agent. Daengsakul’s group [61–63] also synthesized La_1-x_Sr_x_MnO_3_ nanoparticles via thermal decomposition method by using acetate salts of La, Sr, and Mn as starting materials. To control the sizes of the La_1-x_Sr_x_MnO_3_ nanoparticles, thermal decomposition of the precursors was carried out at the different temperatures. Similarly, La_1-x_Sr_x_MnO_3_ nanoparticles (0 ≤ × ≤ 0.5) were synthesized via a simple thermal decomposition method by using acetate salts of La, Sr, and Mn as starting materials in aqueous solution [62]. All the prepared La_1-x_Sr_x_MnO_3_ (*x* ≤ 0.3) nanoparticles had a perovskite structure with transformation from cubic to rhombohedral as the thermal decomposition temperature was over 900 °C, while the others remained cubic structure.

#### Other Methods

Moradi et al. [64] reported on the synthesis of La_0.8_Sr_0.2_MnO_3_ nanoparticles with different particle sizes by the microwave irradiation process. Hintze et al. [65] prepared La_1-x_Sr_x_MnO_3_ nanoparticles via a reverse micelle microemulsion, which was based on CTAB used as a surfactant.

### Preparation Methods for 1D Rare Earth-Doped Perovskite Manganite Oxide Nanostructures

Recently, 1D perovskite manganite nanostructures such as nanowires, nanorods, nanotubes, nanofibers, and nanobelts have received much attention due to their unique features as compared with other low-dimensional systems such as 0D perovskite manganite nanostructures (or quantum dots) and 2D perovskite nanostructures (or quantum wells). The two-dimensional quantum confinement while one unconfined direction for the transport of carriers in the 1D perovskite manganite nanostructures, allows it to behave novel electrical transport and magnetic properties that are significantly different from their polycrystalline counterpart due to the nanosized dimensions. Besides, they also offer a good system to investigate the intrinsic size effects of physical properties. Understanding these behaviors at nanoscale dimension is of importance for developing new generation of revolutionary electronic nanodevices. However, there are numerous challenges on the fabrication and synthesis of these nanostructures with well-controlled dimensions, uniform sizes, phase purity, and homogenous chemical compositions. Since structural control is the key step in controlling properties and device performances, recently many physical techniques and chemical synthesis approaches are developed to understand and thereby control the nucleation and growth processes. In the past decade, significant progress has been made in the synthesis of 1D rare earth-doped perovskite manganite oxide nanostructures. The most commonly adopted techniques toward the realization of 1D rare earth-doped perovskite manganite oxide nanostructures are “bottom-up” routes (such as template-based synthesis, hydro/solvothermal synthesis, molten-salt synthesis, solution-based metal–organic decomposition, and electrospinning), and “top-down” approaches (such as focus ion beam (FIB) milling, and nanoimprint lithography (NIL) techniques). Basically, the synthesis routes to 1D rare earth-doped perovskite manganite oxide nanostructures can be divided into two different categories: (i) template-free synthesis, and (ii) template-assisted synthesis, which are briefly delineated in the following.

#### Template-Free Synthesis

Up to date, several template-free methods such as hydro/solvothermal synthesis, MSS method, electrospinning process have been used to synthesize 1D rare earth-doped perovskite manganite oxide nanostructures. For example, single-crystalline perovskite manganite La_0.5_Ca_0.5_MnO_3_ nanowires with an orthorhombic structure were synthesized by a hydrothermal method [66]. These nanowires grew along [100] direction and had uniform diameter (~ 80 nm) with lengths ranging from several to several tens of micrometers. Similarly, single-crystalline La_0.5_Sr_0.5_MnO_3_, La_0.5_Ba_0.5_MnO_3_, and Pr_0.5_Ca_0.5_MnO_3_ nanowires with a cubic structure were also synthesized by hydrothermal method [67–69]. In the Pr_0.5_Ca_0.5_MnO_3_ nanowires, the charge ordering transition was suppressed and a ferromagnetic phase was observed, whereas the antiferromagnetic transition disappeared [69]. Datta et al. [70] also synthesized the single crystalline La_0.5_Sr_0.5_MnO_3_ nanowires with a diameter of ~ 50 nm and a length up to 10.0 μm. It was found that these La_0.5_Sr_0.5_MnO_3_ nanowires had a FM–PM transition temperature (Curie temperature, *T*_C_) at around 325 K, close to the bulk value (~ 330 K) of the single crystal. That indicates that the functional behavior still retains even after the diameter size of the nanowires is reduced down to 45 nm. The electrical transport measurements from a single nanowire demonstrate that the nanowires exhibit an insulating behavior within the measured temperature range from 5 to 310 K, which is similar to the bulk system.

As a simple, one-step and effective method, electrospinning technique is also used to synthesize inorganic and hybrid compound nanofibers [71, 72]. In addition, the fiber sizes can be easily controlled by changing the electrospinning parameters, such as the applied potential, precursor concentrations, viscosity, and flow rate of the solution [73, 74]. The good examples are the La_0.67_Sr_0.33_MnO_3_ nanowires with diameters in a range of 80–300 nm and length of 200 μm synthesized Jugdersuren et al. [75] and the La_0.75_Sr_0.25_MnO_3_ nanofibers synthesized by Huang et al. [76] In addition, multicomponent La_x_Sr_1-x_Co_0.1_Mn_0.9_O_3-δ_ (0.3 ≤ × ≤ 1) and La_0.33_Pr_0.34_Ca_0.33_MnO_3_ nanofibers are also synthesized by electrospinning method, which can be used as cathode materials in the next-generation high-performance supercapacitors and phase separation nanodevices, respectively [77, 78].

Rare earth-doped perovskite manganite oxide nanorods are also synthesized by using template-free method such as hydrothermal synthesis. For example, La_0.65_Sr_0.3_MnO_3_ nanorods were successfully synthesized through a simple hydrothermal reaction followed by calcination at 850 °C for 2 h in air. Small nanorods having a diameter in the range of 80–120 nm tend to connect with each other forming long rods with length of a few hundred nm to a few micron [79]. Nano-sized La_0.7_Ca_0.3_MnO_3_ manganites with rod-like morphologies were also obtained via the hydrothermal method in the presence of two mineralizers of sodium hydroxide (NaOH) and potassium hydroxide (KOH) at different alkalinity conditions (10, 15, and 20 M) [80].

#### Template-Assisted Methods

The template-assisted method is to use the pre-existing 1D nanostructures (e.g., nanoporous silicon, polycarbonate membranes, anodic aluminium oxide (AAO) membranes) as templates, which are filled up with the suitable polymeric precursors. The solution contained within the template is heat treated to form perovskite manganite oxide materials, and subsequently removing the template by chemical etching or calcination. Synthesis of 1D perovskite manganite oxide nanostructures through template-assisted method offer the following advantages: (a) the structure of the nanoarrays is subject to the structure of the template, (b) the channels of the template control the dimension sizes of the materials, (c) pore walls of template prevent the aggregation of the material, and (d) a large amount of nanowires or nanotubes can be massively produced. Among the common used template-assisted methods, the sol-gel template method combined with AAO as template is the most popular one, which is widely used to fabricate highly ordered perovskite manganite oxide nanostructures such as La_0.8_Ca_0.2_MnO_3_ nanowires with nearly uniform diameter of about 30 nm [81], and the ordered array of La_0.67_Ca_0.33_MnO_3_ nanowires with diameter of 60–70 nm and tens of microns in length [82]. Following the success of this method, perovskite manganite oxide nanowires of La_0.6_Sr_0.4_CoO_3_ and La_0.825_Sr_0.175_MnO_3_ with a diameter of 50 nm and length up to tens of microns were also synthesized with a polycrystalline perovskite structure [83]. Ordered arrays of La_0.67_Sr_0.33_MnO_3_ nanowires with diameter of 60–70 nm and length up to tens of microns were prepared using a simple sol-gel process combining with nanoporous alumina as template [84].

Optical lithography is also used to fabricate (La_5/8-0.3_Pr_0.3_)Ca_3/8_MnO_3_ (LPCMO) wires starting from a single crystalline LPCMO film epitaxially grown on a LaAlO_3_(100) substrate [85]. As the width of the wires is decreased, the resistivity of the LPCMO wires exhibits giant and ultrasharp steps upon varying temperature and magnetic field in the vicinity of the M–I transition. The origin of the ultrasharp transitions can be ascribed to the effect of spatial confinement on the percolative transport in manganites. Han et al. [86] fabricated the MgO/La_0.67_Ca_0.33_MnO_3_ core-shell nanowires with the inner MgO core about 20 nm in diameter and the La_0.67_Ca_0.33_MnO_3_ shell layer around 10 nm in thickness. Here, the vertically aligned single-crystalline MgO nanowires act as excellent templates for epitaxial deposition of the desired transition metal oxides and lead to high-quality core-shell nanowires.

Besides the perovskite manganite oxide nanowires, perovskite manganite oxide nanotubes are also fabricated by using a sol-gel template-based method. Curiale et al. [87] synthesized the perovskite rare earth manganite oxide nanotubes such as La_0.67_Sr_0.33_MnO_3_, La_0.67_Ca_0.33_MnO_3_, and La_0.325_Pr_0.300_Ca_0.375_MnO_3_, by using a sol-gel template synthesis process. The typical length of the nanotubes was about 6 to 8 μm, and the average wall thickness was 45, 60, and 150 nm for the La_0.67_Sr_0.33_MnO_3_, La_0.67_Ca_0.33_MnO_3_, and La_0.325_Pr_0.300_Ca_0.375_MnO_3_, respectively. The walls of these nanotubes are composed of magnetic nanograins, and their sizes are less than the critical size for multidomain formation in manganites. As a consequence, each particle that constitutes of the nanotube walls is a single magnetic domain.

Highly ordered perovskite manganite La_2/3_Ca_1/3_MnO_3_ nanotube arrays (with uniform diameter of 80 nm) were also successfully synthesized by a simple and rapid process, combining AAO template-assisted synthesis with microwave irradiation [88]. This method offers a quick hands-on route to produce nanotube arrays at relative low temperatures. Rare earth manganese oxide nanotubes with nominal composition of La_0.325_Pr_0.30_Ca_0.375_MnO_3_ (800 nm external diameter, 4 μm length, and wall thickness below 100 nm) were synthesized by pore wetting of porous polycarbonate templates with the liquid precursor, and then followed by microwave irradiation and a further calcination at 800 °C (two-stage thermal treatment) [89]. The wall thickness of these nanotubes was found to be formed by small crystals of approximately 20 nm. Perovskite La_0.59_Ca_0.41_CoO_3_ nanotubes prepared by a sol-gel template method can be used as the catalysts in the air electrode for oxygen evolution, demonstrating superior catalytic activity and durability in comparison with that of the electrodes made by nanoparticles [90]. This indicates a promising application of La_0.59_Ca_0.41_CoO_3_ nanotubes as electrocatalysts of air electrodes in fuel cells and rechargeable metal–air batteries. Perovskite Sm_0.6_Sr_0.4_MnO_3_ nanotubes with diameter of 200 nm were also prepared by a sol-gel template method. Their walls are composed of nanoparticles with a diameter of 25 nm [91]. However, in these processes, the templates are usually dipped into the sols directly with the only driving force of capillary action. In the case of higher concentration sol, filling the pores become much difficult, especially for the templates with small pore diameters. While in the case of the sol with lower concentration, it usually results in serious shrinkage and cracking of porous templates during annealing process. Therefore, the synthesis of rare-doped perovskite manganite nanotubes with high crystallized quality by template-assisted method is still much challenging.

## Synthesis Methods for 2D Rare Earth-Doped Perovskite Manganite Oxide Nanostructures

2D rare earth-doped perovskite manganite oxide nanostructures include perovskite manganite oxide thin films, nanodot arrays, nanosheets, nanoplates, nanowalls, which exhibit interesting physical properties due to their complex interplays of spin, charge, orbital, and lattice degrees of freedom. They have promising applications in the fields of high-density memory and storage, sensors, and spintronic devices. Therefore, in the past few years, several methods have been developed to fabricate 2D rare earth-doped perovskite manganite oxide nanostructures [92–94]. For the reason of clarity, this section is divided into three subsections: current works on earth-doped perovskite manganite oxide thin films and/or multilayers, 2D earth-doped perovskite manganite oxide nanostructures based on planar structures, and rare earth-doped perovskite manganite oxide nanosheets.

### Rare Earth-Doped Perovskite Manganite Oxide Thin Films or Multilayers

The growths of rare earth-doped perovskite manganite oxide thin films or multilayers are the process of taking the starting materials to be turned into films or multilayers and producing from its atoms, molecules, or ions in a gaseous state, which are then deposited onto the surface of a clean substrate. The prepared methods used to convert the starting materials into atomic, molecular, or ionized states are also diverse, which include physical vapor deposition (PVD) methods such as pulsed laser deposition (PLD), vacuum vapor deposition, RF magnetron sputtering, and chemical methods such as chemical solution deposition (CSD), chemical vapor deposition (CVD), metalorganic chemical vapor deposition (MOCVD), and molecular beam epitaxy (MBE). In the following sections, the most widely used techniques, including PLD, CSD, CVD, and MOCVD, and MBE techniques will be shortly introduced.

### Pulsed Laser Deposition

PLD is a thin film deposition technique, in which thin film is grown by the ablation of one or more targets illuminated by a focused pulsed-laser beam [95]. In this method, a high power of pulsed laser beam is focused inside a vacuum chamber to strike a target of the material that is to be deposited. PLD process generally can be divided into the following four stages [96]: the laser radiation interaction with the target, dynamic of the ablation materials, decomposition of the ablation materials onto the substrate, nucleation and growth of a thin film on the substrate surface. PLD has several attractive features, including the stoichiometric transfer of material from the target, generation of energetic species, hyperthermal reaction between the ablated cations and molecular oxygen in the ablation plasma, and compatibility with background pressures ranging from ultra-high vacuum (UHV) to 100 Pa. Among them, the most feature characteristic of the PLD process is the ability to realize a stoichiometric transfer of the ablated material from a multi-cation target for many materials, achieving a composition of the film that is almost identical with that of the target, even though the target involves a complex stoichiometry. Moreover, the ability to easily vary the deposition rate is one of the principal features of PLD compared to other physical vapor deposition methods such as the sputtering technique. By controlling the growth conditions (e.g., the substrate temperature, chamber pressure, laser influence, target-to-substrate distance), many perovskite manganite oxide thin films or multilayers can be grown for high-performance electrical, magnetic, and optical devices. For example, Lawler et al. [97] grew the La_1-x_Ca_x_MnO_3_ thin films by PLD, which were ferromagnetic when 0.2 ≤ × ≤ 0.5 with *T*_C_ *≈* 250 K. Harzheim et al. [98] also grew the La_0.66_Ba_0.33_MnO_3_ films (with a thickness range of 5 to 250 nm) by PLD. Their CMR effects are dependent upon the thickness of epitaxial thin films deposited on MgO (100) and SrTiO_3_ (STO) (100). A giant magnetoresistance near room temperature was observed in the ferromagnetic films of La_1-x_Sr_x_MnO_3_ (0.16 ≤ × ≤ 0.33) grown on (100) SrTiO_3_ substrates by PLD [99]. Atomically defined epitaxy of the La_0.6_Sr_0.4_MnO_3_ thin films with MnO_2_ atomic layer as the terminating layer was also achieved by PLD method. The film as thin as 4 nm still shows a clear magnetic transition at *T*_C_ = 240 K, semimetallic conduction below *T*_C_, and a novel magnetoresistive behavior down to the lowest temperature. Other rare earth-doped perovskite manganite oxide thin films such as La_0.6_Pb_0.4_MnO_3_ [100], Nd_0.7_Sr_0.3_MnO_z_ [101], Sm_1-x_Sr_x_MnO_3_ [102], and Pr_0.5_Ca_0.5_MnO_3_ [103] were also in situ deposited at different temperatures and oxygen partial pressures by PLD process. To check effects of strains in the charge-ordered epitaxial Pr_1-x_Ca_x_MnO_3_ (*x* = 0.5, 0.6) thin films deposited on LaAlO_3_ (LAO) and SrTiO_3_ (STO) substrates, Haghiri-Gosnet et al. [104] carried out the Raman studies of the Pr_1-x_Ca_x_MnO_3_ films with different thickness. They found that the A_g_(2) mode (related to the tilting angle of the MnO_6_ octahedra) was highly sensitive to the local changes and distortions in the lattice caused by the variations in temperature, doping, and epitaxial strains. Dhakal et al. [105] performed the epitaxial growth of (La_1-*y*_Pr_*y*_)_0.67_Ca_0.33_MnO_3_ (LPCMO) (with *y* = 0.4, 0.5, and 0.6) thin films on NdGaO_3_ (NGO) (110) and STO (100) substrates by PLD, and the effect of spatial confinement on EPS in the La_0.325_Pr_0.3_Ca_0.375_MnO_3_ single-crystalline disks with diameters in the range of 500 nm–20 μm (fabricated from epitaxial LPCMO thin films by electron beam lithography) was investigated by Shao et al. [106]. It is found that the EPS state still remains to be the ground state in disks with the diameter of 800 nm or larger whereas vanishes in the 500-nm-diameter disks whose size is distinctly smaller than the characteristic length scale of the EPS domains. In the 500-nm-dameter disks, only the ferromagnetic phase was observed at all temperatures below Curie temperature Tc, indicating that the system was in a single-phase state rather than a EPS state. Kurij et al. [107] reported that all-oxide magnetic tunnel junctions with a semiconducting barrier, formed by the half-metallic ferromagnetic La_0.7_Sr_0.3_MnO_3_ and n-type semiconductor SrTi_0.8_Nb_0.2_O_3_, were designed. Multilayers with the compositions of La_0.7_Sr_0.3_MnO_3_ (30 nm)/Nb:STO (1.8–3.0 nm)/La_0.7_Sr_0.3_MnO_3_ (10 nm)/La_0.7_Sr_0.3_Mn_0.93_Ru_0.07_O_3_ (20 nm) were grown in situ by pulsed laser deposition on TiO_2_ single-terminated, (100)-oriented STO substrates. The Nb:STO layer thickness in the junctions varied from 1.8 to 3.0 nm, and the additional 10-nm-thick La_0.7_Sr_0.3_MnO_3_ layer helped to avoid Ru diffusion into the barrier. It is found that tunnel junctions with an Nb:STO barrier exhibit an enhanced quality with a reduced number of defects, resulting in improved reproducibility of results, large TMR ratios between 100 and 350% between 20 and 100 K, and also a three orders of magnitude improvement of the low-frequency noise level. These results open the way to all oxide sensors for magnetometry applications. Xu et al. [108] reported on the epitaxial of La_0.7_Sr_0.3_MnO_3_/SrRu_1-x_Ti_x_O_3_ (SR_1-x_T_x_O) superlattices on (001)-oriented (LaAlO_3_)_0.3_(SrAl_0.5_Ta_0.5_O_3_)_0.7_ (LSAT) and (001)-oriented NGO single crystal substrates by PLD. Good reviews on the epitaxial growth of perovskite oxide thin films and superlattices can be found in the literatures [92–94].

### Chemical Solution Deposition

CSD is also named as solution growth, controlled or arrested precipitation, etc. Chemical deposition of perovskite thin films results from moderately slow chemical reaction that leads to the formation of thin solid layer onto the immersed substrate surface at the expense of chemical reaction between the aqueous precursor solutions [109–111]. In this method, when cationic and anionic solutions are mixed together and if the ionic product exceeds or becomes equal to the solubility product, precipitation occurs as ions combine together on the substrate and in the solution to from nuclei. Perovskite manganite oxide thin films can be grown on either metallic or nonmetallic substrates by dipping them in appropriate solutions of metal salts without the application of any electric field. Deposition may occur by homogeneous chemical reaction, usually reduction of metal ions, in a solution by a reducing agent. The growth rate and the degree of crystallinity depend upon the temperature of the solution. This method has many advantages such as large area thin film depositions, deposition at low temperature, and avoiding oxidation or corrosion of the metallic substrates [112].

Up to date, many perovskite manganite oxide thin films or multilayers have been synthesized by CSD method. Hasenkox et al. [113] reported on a flexible CSD method for the preparation of magnetoresistive La_1-x_(Ca,Sr)_x_MnO_3_ thin films based completely on metal propionates. Tanaka et al. [114] also grew (La,Sr)MnO_3_ thin films on STO (100) single crystal substrates by CSD method. Solanki et al. [115] measured the transport and magnetotransport properties of the La_0.7_Pb_0.3_MnO_3_ thin films grown on single crystal LAO (100) substrates by CSD technique. The structural, surface, and electrical properties of the La_0.7_Ca_0.3_MnO_3_ and La_0.7_Sr_0.3_MnO_3_ thin films deposited on (100)*-*oriented LAO single crystal substrates by CSD technique were also investigated [116, 117]. The Pr-doped La_0.8-x_Pr_0.2_Sr_x_MnO_3_ (*x* = 0.1, 0.2, and 0.3) thin films were also grown on STO (100) single crystal substrate by CSD method [118]. Details about the growth of perovskite manganite oxide thin films by CSD method can found in good reviews contributed from Schwartz [111] and Zhang et al. [119].

### CVD and MOCVD

CVD is one of the most popular routes to synthesize perovskite oxide functional nanomaterials. It is often used to prepare high-quality, high-performance thin films on large area wafers or complex patterned substrates. The key difference from CSD is that instead of solutions as precursors, materials are prepared by CVD via the deposition of gaseous precursor onto the substrate. Thus, it requires high vapor pressure composition as the precursor and often the substrate must be heated to a particular temperature to facilitate the deposition reaction as well as the motion of adatoms [120]. In the CVD process, the film composition and structure are rather sensitive to the substrate temperature, the precursor delivery ratio, and the vaporizer temperature. CVD processes have the advantage of high deposition rate and low deposition temperature. As compared with the CSD process, they offer much better control over the morphology, crystal structure and orientations, and as a result are often used to prepare epitaxial perovskite oxide thin films [121–123]. Herrero et al. [124] reported on the growth of perovskite manganite La_1-x_A_x_MnO_3_ (A = Ca, Sr) thin films by a modified CVD process.

When metal-organic compounds are used as precursors, the process is generally referred to as MOCVD, which is a popular CVD method and commonly used in Si technologies and electronic device fabrication for the synthesis of thin films and coatings. This technique offers several potential advantages over other physical deposition processes such as (i) high degree of control in stoichiometry, crystallinity, and uniformity; (ii) a versatile composition control; and (iii) the ability to coat complex shapes and large areas. Depending upon the processing conditions, different MOCVD variants are available, for example, low-pressure MOCVD, atmospheric pressure MOCVD, direct liquid injection MOCVD, and plasma-enhanced MOCVD [125]. In the direct liquid injection MOCVD, microdroplets of precursor solution controlled by a the computer are injected into the evaporator system. These droplets are produced by a high-speed electro-valve. The frequency and the time of the injection can be well adjusted so as to achieve the appropriate growth rate for each deposited material. Therefore, the final film stoichiometry can be precisely controlled by adjusting the respective concentrations of the precursors in the precursor liquid source. Up to date, MOCVD has been successfully used for growths of perovskite manganite oxide thin films or multilayers such as La_1-x_Sr_x_MnO_3_ [126], Pr_1-x_Ca_x_MnO_3_ [127], and perovskite oxide superlattices such as (La_0.7_Sr_0.3_MnO_3_/SrTiO_3_)_15_ [128].

### Molecular Beam Epitaxy

The molecular beam epitaxy (MBE) growth of thin films may be thought of as atomic spray painting, in which alternately shuttered elemental sources are employed to control the cation stoichiometry precisely, thus producing perovskite oxide thin films of exceptional quality. The flux of spray from each atomic or molecular beam is controlled by the temperature (and thus vapor pressure) of the effusion cell in which each species is contained. The duration of spray is individually controlled for each beam by shutters, which control not only the open time (and thus dose) but also the sequence in which species reach the growth surface. By controlling the shutters and temperature of the evaporant (which control dose and flux, respectively), the layering sequence of the desired structure can be customized. This technique is the premiere synthesis technique for the synthesis of layered oxides with customized layering control down to the atomic layer level [94]. Reutler et al. [129] reported on the growth of La_2/3_Ca_1/3_MnO_3_ films by laser molecular beam epitaxy on (001)-oriented STO and NGO single-crystal substrates. The film thickness was 200 nm for the films on STO and 40 nm for the films on NGO. Werner et al. [130] reported that resistance versus magnetic field measurements for a La_0.65_Sr_0.35_MnO_3_/SrTiO_3_/La_0.65_Sr_0.35_MnO_3_ tunnel junction grown by MBE, which showed a large field window of extremely high TMR at low temperatures. Peng et al. [131] systematically studied the dead-layer behavior of La_0.67_Sr_0.33_MnO_3_ (LSMO)/STO heterostructures grown by ozone-assisted molecular beam epitaxy (OMBE). They found that the low kinetic energy of atomic beam could reduce extrinsic defects to the lowest level, and the composition was easily tuned at the single-atomic-layer level. Matou et al. [132] reported the reduction of the dead layer by growing La_0.67_Sr_0.33_MnO_3_ on a LaMnO_3_ layer, and they demonstrated the detection of TMR in the La_0.67_Sr_0.33_MnO_3_/LaMnO_3_/La_0.67_Sr_0.33_MnO_3_ heterostructures grown by MBE with a shuttered growth technique.

## 2D Earth-Doped Perovskite Manganite Oxide Nanostructures Based on Planar Structures

### Top-Down Methods

In recent years, 2D earth-doped perovskite manganite oxide nanostructures based on planar structures such as nanoplates [133] or lamella [134] and lateral arrays of nanodots [135] or nanowires [85] are fabricated. Different forms of “top-down” such as electron beam lithography (EBL) and NIL have been used for the geometrical patterning of 2D perovskite manganite nanostructures.

Singh-Bhalla et al. [136, 137] fabricated (La_0.5_Pr_0.5_)_0.67_Ca_0.33_MnO_3_ nanobridges and microbridges with a width ranging from 100 nm to 1 μm by a combination of photolithography and FIB. They first deposited single crystalline, epitaxial 30-nm-thick (La_0.5_Pr_0.5_)_0.67_Ca_0.33_MnO_3_ films on the NdGaO_3_ (110) substrates at 820 °C by PLD, and then a combination of photolithography and FIB was employed to fabricate the (La_0.5_Pr_0.5_)_0.67_Ca_0.33_MnO_3_ nanobridges and microbridges. Peña et al. [138] also fabricated La_2/3_Sr_1/3_MnO_3_ microbridges by standard photolithographic techniques. La_0.7_Sr_0.3_MnO_3_ nanobridges with dimensions of less than 20 nm were also fabricated by FIB from the corresponding epitaxial thin film [139].

EBL is another nanofabrication technique in rapid development. Guo et al. [140] grew La_0.67_Ca_0.33_MnO_3_ films with thickness of ~ 100 nm on STO (100) substrates by a PLD technique, and fabricated the La_0.67_Ca_0.33_MnO_3_ microbridges with different widths (e.g., 1.5 μm, 1 μm, and 0.50 μm) via EBL technology. Beekman et al. [141] also grew thin La_0.7_Ca_0.3_MnO_3_ films (with a thickness range of 20–70 nm) on STO (001) substrates by DC sputtering. And then, they fabricated the La_0.7_Ca_0.3_MnO_3_ microbridges with a width of 5 μm by using EBL technology and Ar^+^ etching.

### Bottom-Up Methods

Besides the top-down methods, bottom-up methods such as template-assisted synthesis are also used to fabricate 2D perovskite manganite oxide nanostructures based on lateral arrays of nanodots. In contrast, template-assisted “bottom-up” synthetic approaches provide a route to achieving 2D geometrical ordering of perovskite manganite nanostructures with narrow size distributions. Nanosphere lithography (NSL) has been demonstrated as a versatile template-based method for generating 2D perovskite manganite nanostructures [142]. In NSL, the spacing and size of the periodically arranged nanostructures can be readily controlled by using polymer spheres with different diameters, and/or by changing the amount of material deposited. For example, Liu et al. [143] prepared two-dimensional oxide nanoconstriction arrays via NSL. They dropped a drop of aqueous suspension of SiO_2_ microspheres, with a diameter of 1.5 μm, onto a STO (100) substrate. These microspheres could self-assemble during the drying process and finally turned into a hexagon-like ordered monolayer. Then, a reactive ion etching process was proceeded to reduce the sizes of the microspheres. Subsequently, the substrate was put into a PLD chamber for the deposition of La_0.67_Sr_0.33_MnO_3_, after that the sample was transferred into a furnace and annealed at 750 °C. After removing the microspheres, a La_0.67_Sr_0.33_MnO_3_ nanoconstriction dot array was obtained. Under the low oxygen pressure, the La_0.67_Sr_0.33_MnO_3_ film was deposited with the oxygen deficiency in La_0.67_Sr_0.33_MnO_3_ nanoconstriction; the sample had to be further annealed at 900 °C for 8 h in air. Finally, the La_0.67_Sr_0.33_MnO_3_ nanoconstriction dot arrays were obtained with sizes about 100 nm.

### Synthesis Methods for 3D Rare Earth-Doped Perovskite Manganite Oxide Nanostructures

**Fig. 1:**
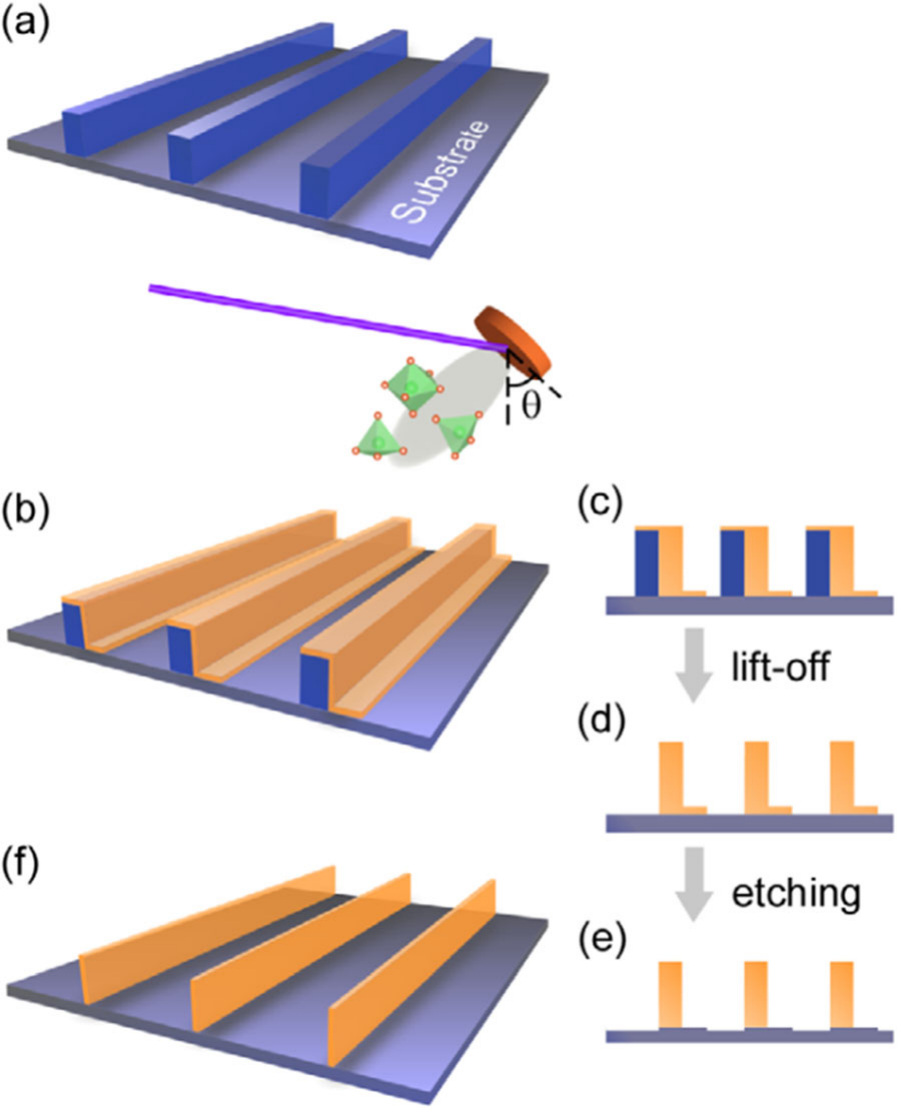
Schematic flowchart of the 3D nano-template PLD method for perovskite oxide nanostructure fabrication. **a** First, template wall structures are patterned onto substrate by NIL using an organic resist (blue region). **b** Functional perovskite oxides (orange region) is then deposited onto the side surface of the template patterns by PLD. **c** Cross-sectional image of (**b**). Cross-sectional images for nanowall-wire structure after **d** liftoff and **e** etching. **f** Finally, self-standing perovskite oxide nanowall-wire arrays are obtained. Reproduced with permission of [144]

**Fig. 2:**
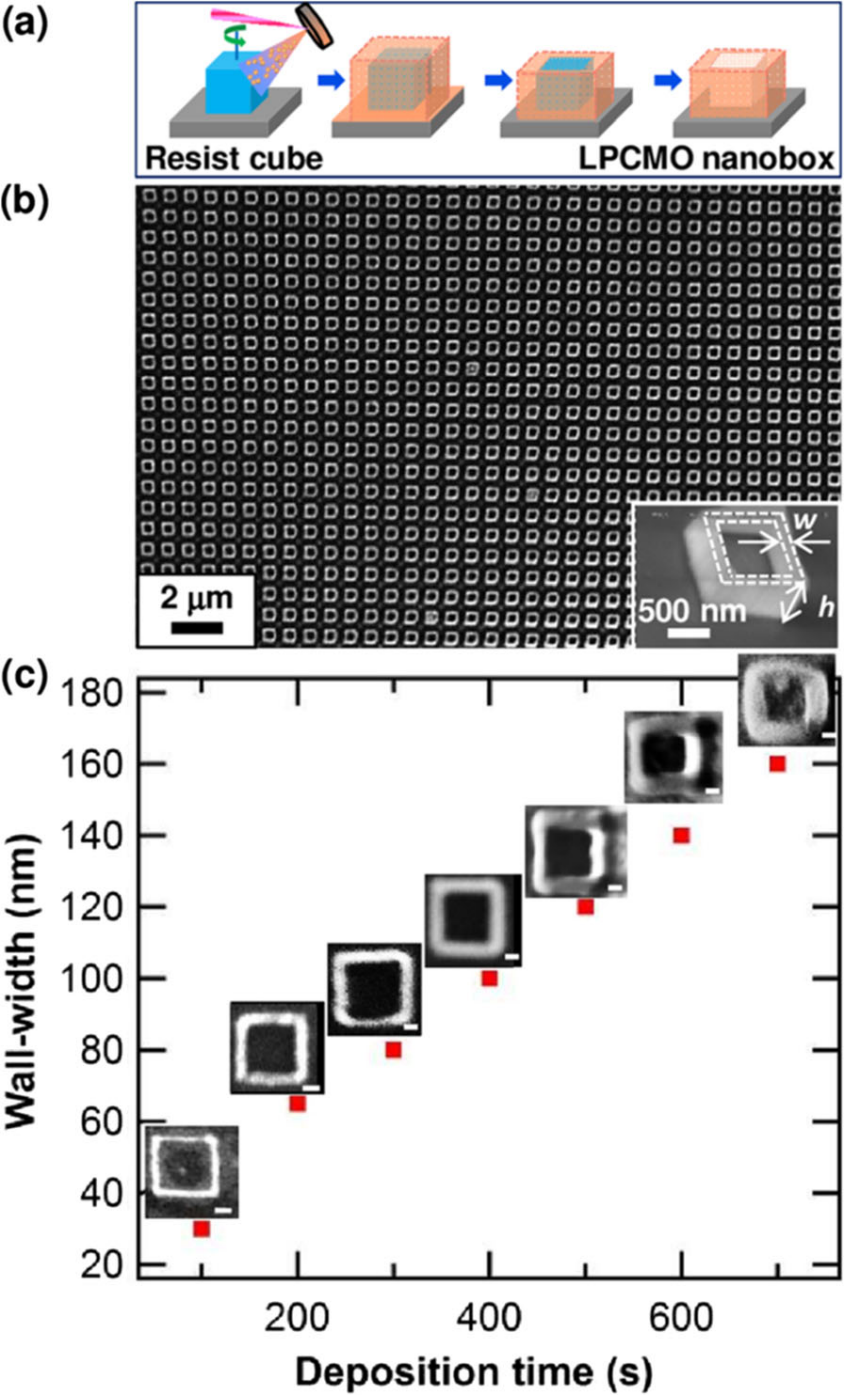
**a** Schematic image for the fabrication procedure of the (La_0.275_Pr_0.35_Ca_0.375_)MnO_3_ (LPCMO) nanoboxes. **b** Typical SEM image of (La_0.275_Pr_0.35_Ca_0.375_)MnO_3_ nanoboxes with 100 nm wall-width and 400 nm height. **c** The relationship between the wall-width of the (La_0.275_Pr_0.35_Ca_0.375_)MnO_3_ nanoboxes and the deposition time. Reproduced with permission of [145]

Basically, there are two approaches for fabricating 3D perovskite-type oxide nanostructures: “bottom-up” and “top-down.” “Bottom-up” processing refers to the synthesis of nanostructures starting at the atomic or molecular level. Solution-based routes (e.g., sol-gel based chemical solution deposition, templating, solution phase decomposition, and hydro/solvothermal synthesis) are the most commonly employed in the “bottom-up” approaches for synthesizing 3D perovskite-type oxide nanostructures (i.e., vertically aligned nanowires, rods or tubes). “Top-down” processing, e.g., FIB milling and some lithographical methods such as NIL, consists of carving away at a bulk material to create coherently and continuously ordered nanosized structures. Recently, 3D perovskite manganite oxide nanostructures are prepared by 3D nano-template PLD method. The basic concept of this method is an inclined substrate deposition onto the side surfaces of a 3D nano-patterned substrate, i.e., 3D nano-template is schematically shown in Fig. 1 [144]. At first, template wall structures are patterned on substrate by NIL technique using an organic resist (blue region) (Fig. 1a). Target material, i.e., metal oxide, is then deposited onto the side surface of the template patterns by PLD (Fig. 1b). After liftoff of templates and then etching for residual bottom film (Fig. 1c, d), self-standing metal oxide nano-wall wire arrays are obtained (Fig. 1e, f). Due to the right-angle side surface, the 3D nanotemplate acts as a shape and position reference point. The deposited material starts to grow at the side surface (interface) of the 3D nano-template while translating its shape. Therefore, the formation of nanostructures beyond the resolution limitations of top-down methods is realized. Recently, precisely size-controlled and crystalline (La_0.275_Pr_0.35_Ca_0.375_)MnO_3_ nanobox were fabricated on a MgO (001) substrate using the this method [145]. In this process (see Fig. 2a), the MgO(001) substrate was first patterned with the organic resist cubes by NIL technique. And then, the (La_0.275_Pr_0.35_Ca_0.375_)MnO_3_ was deposited by the PLD technique on the four side-surfaces of the resist cube at room temperature (RT). The (La_0.275_Pr_0.35_Ca_0.375_)MnO_3_ nanoboxes were obtained in a large area (~ 20 mm^2^) after the (La_0.275_Pr_0.35_Ca_0.375_)MnO_3_ top layer, and the inner core resist were removed. To improve the crystallinity, the post-annealing process was carried out at 1270 K under the oxygen pressure of 1 Pa. The typical SEM image of the (La_0.275_Pr_0.35_Ca_0.375_)MnO_3_ nanoboxes is shown in Fig. 2b. The wall-width of nanoboxes were successfully controlled in a range from 160 nm down to 30 nm by changing the deposition time, as shown in Fig. 2c. These (La_0.275_Pr_0.35_Ca_0.375_)MnO_3_ nanoboxes exhibited the insulator–metal transition at the higher temperature than that in the corresponding film. This indicates that the well-aligned and reliably prepared, highly integrated CMR manganite 3D nanoboxes can provide a way to tune the physical properties of the CMR oxides. 3D nanotemplate PLD technique can be used to fabricate various perovskite manganite oxide nanostructures.

## Structural Characterization of Rare Earth-Doped Perovskite Manganite Oxide Nanostructures

### Introduction

The structural characterizations of rare earth-doped perovskite manganite oxide nanostructures are conducted to investigate their crystal structures, chemical compositions, and morphologies. The crystal structures are usually characterized by X-ray diffraction (XRD), Raman spectrum, Fourier-transform infrared spectroscopy (FTIR), field-emission scanning electron microscopy (FE-SEM), transmission electron microscopy (TEM), high-resolution TEM (HRTEM), and selected area electron diffraction (SAED). The chemical compositions are usually examined by energy dispersive X-ray spectroscopy (EDS), electronic energy loss spectroscopy (EELS), and X-ray photoelectron spectroscopy (XPS). The chemical bonding and chemical structure of the prepared rare earth-doped perovskite manganite oxide nanostructures can be examined by XPS, EELS, FTIR, and Raman spectra. The morphologies are usually characterized by atomic force microscopy (AFM), scanning electron microscopy (SEM), and TEM. In this section, the structural characterizations of rare earth-doped perovskite manganite oxide nanostructures are described to provide a brief review of the microstructural characterizations of rare earth-doped perovskite manganite oxide nanostructures.

### Rare Earth-Doped Perovskite Manganite Oxide Nanoparticles

**Fig. 3:**
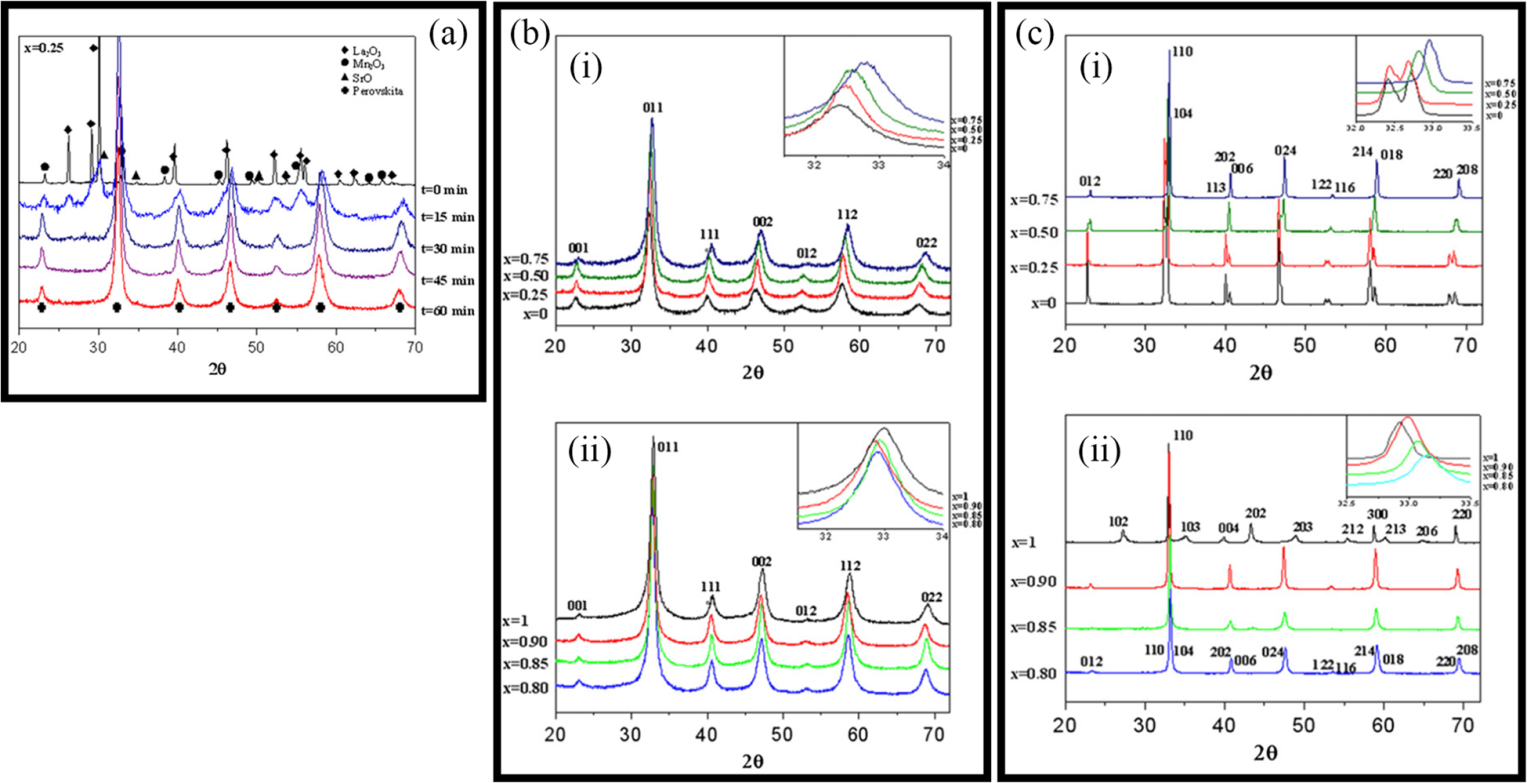
**a** XRD patterns of the La_1-x_Sr_x_MnO_3±δ_ (*x* = 0.25) nanoparticles synthesized by mechanochemistry method under different milling time. **b** XRD patterns of the La_1-x_Sr_x_MnO_3±δ_ (0 ≤ × ≤ 1) nanoparticles with different substitutions of La by Sr modifiers. **c** XRD patterns of the La_1-x_Sr_x_MnO_3±δ_ (0 ≤ × ≤ 1) nanoparticles heat treated at 1000^o^C under static air. (i) *x* = 0, 0.25, 0.50, and 0.75, and (ii) *x* = 0.80, 0.85, 0.90, and 1.0. The inset shows an enlargement of the highest maxima. Reproduced with permission of [147]

**Fig. 4:**
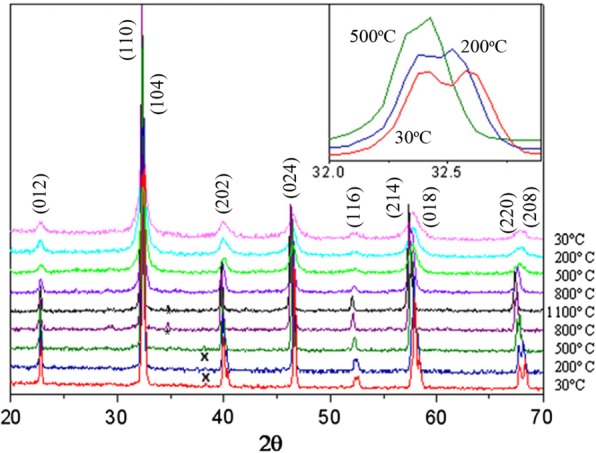
Thermal evolution of the XRD patterns of the La_0.75_Sr_0.25_MnO_3±δ_ sample as a function of the temperature from 30 to 1100 °C (up and down) performed under air atmosphere. The inset shows an enlargement of the highest maxima. Reproduced with permission of [147]

**Fig. 5:**
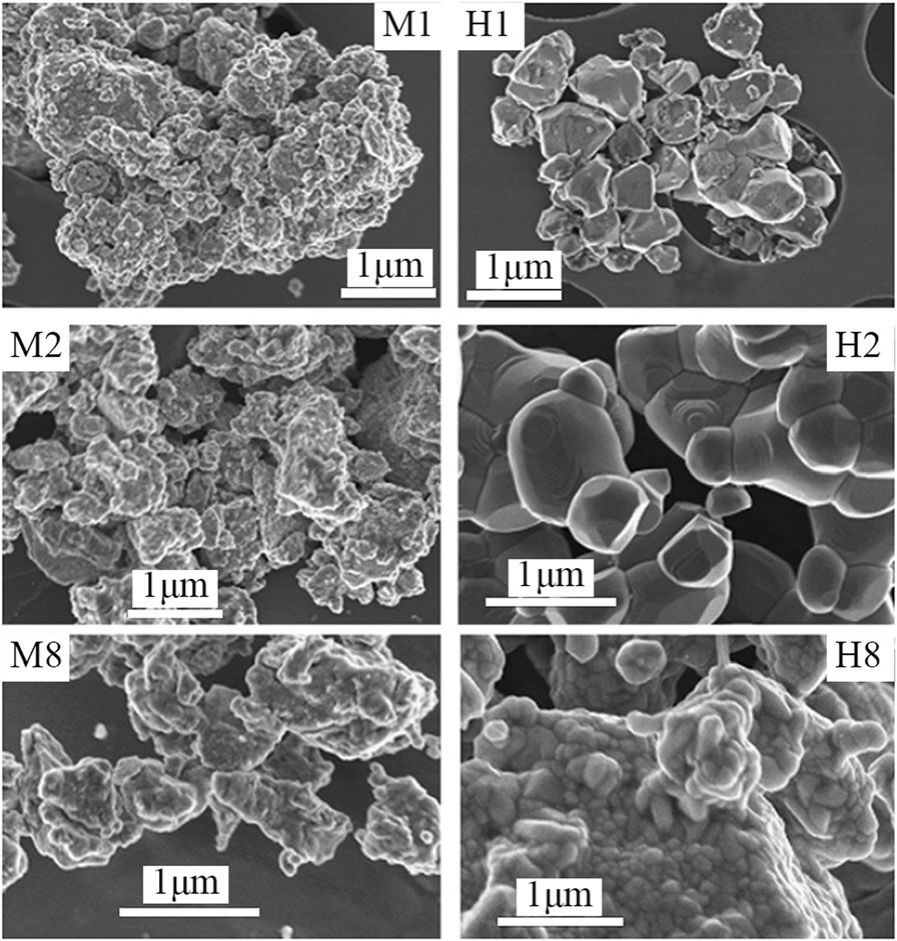
SEM images of the corresponding to samples M1 and H1 (*x* = 0), M2 and H2 (*x* = 0.25), and M8 and H8 (*x* = 1.0). The samples in the La_1-x_Sr_x_MnO_3±δ_ (0 ≤ × ≤ 1) system with the first step after milling refer to M samples and after heated at 1100 °C under air atmosphere during 12 h refer to H samples. Reproduced with permission of [147]

**Fig. 6:**
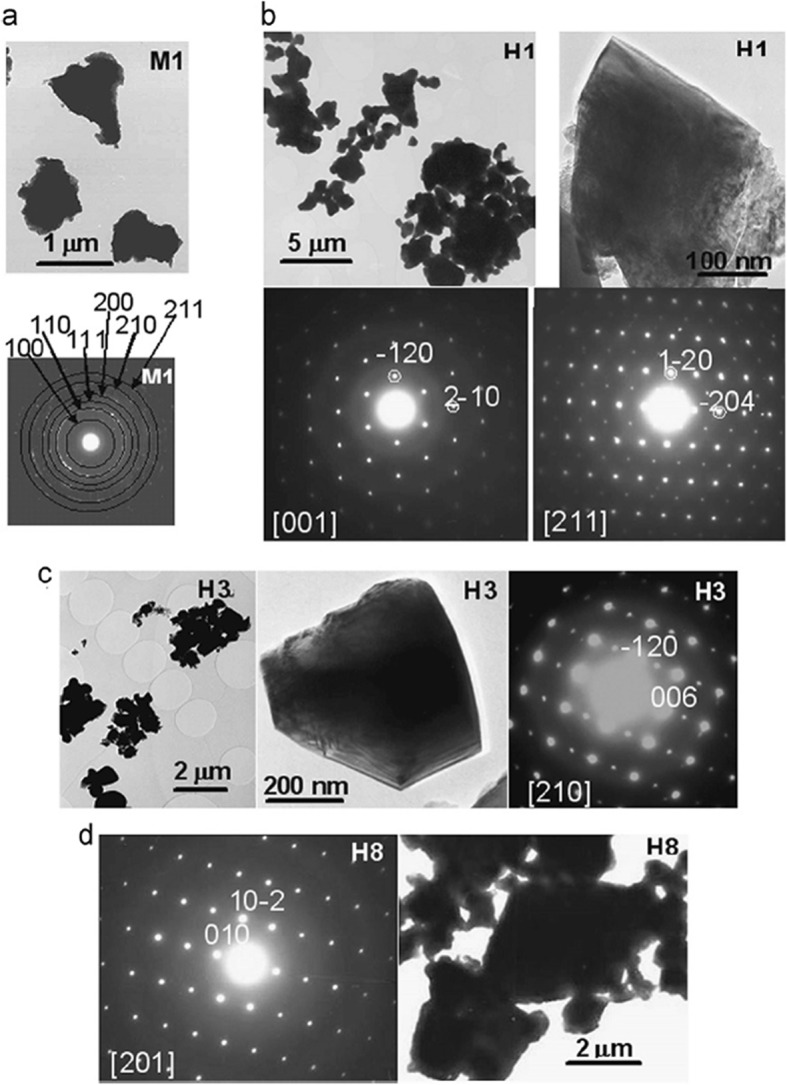
TEM images and SAED patterns corresponding to **a** M1 (*x* = 0), **b** H1 (*x* = 0), **c** H3 (*x* = 0.50), and **d** H8 (*x* = 1.0) samples. The samples in the La_1-x_Sr_x_MnO_3±δ_ (0 ≤ × ≤ 1) system with the first step after milling refer to M samples and after heated at 1100 °C under air atmosphere during 12 h refer to H samples. Reproduced with permission of [147]

Up to date, many rare earth-doped perovskite manganite oxide nanoparticles have been synthesized by physical or chemical methods. Their physical and chemical properties are dependent upon the phase structures, morphologies, chemical compositions, and the grain size distributions of the nanoparticles as well as their thermal history during the synthesized process [146]. XRD is often used to identify the phase structure and the relative percents of different phases of the prepared nanomaterials. In addition, some structural parameters such as particle size, lattice parameters (a, b, and c), lattice volume, and theoretical density can be derived from the XRD data. Also, XRD is also used to optimize the preparation conditions of rare earth-doped perovskite manganite oxide nanoparticles [147–149]. For example, Sayague’s et al. [147] synthesized the La_1-x_Sr_x_MnO_3±δ_ (0 ≤ × ≤ 1) nanoparticles by mechanochemistry synthesis method under different conditions (e.g., different substitutions of La by Sr modifiers; various milling time; heat treatment at 1000 °C under static air), and the XRD patterns of these samples are shown in Fig. 3. Figure 3a shows the XRD patterns of the La_1-x_Sr_x_MnO_3±δ_ (*x* = 0.25) nanoparticles synthesized at different milling time. It was clearly observed that the solid state reaction during mechanochemistry synthesis process progressed significantly after 15 min milling and after 30 min it was almost finished. After only 45 min, no reactant peaks were detected and the solid-state reaction seemed to be complete. To ensure the full conversion, the mechanochemical synthesis of the nanoparticles was then carried out by 60 min ball milling. Figure 3b demonstrates the XRD patterns of the La_1-x_Sr_x_MnO_3±δ_ (0 ≤ × ≤ 1) nanoparticles with different substitutions of La by Sr modifiers obtained by mechanochemistry synthesis. All the nanoparticles crystallized in a single phase with pseudo-cubic symmetry and perovskite structure. The right-shift of the XRD reflections in 2θ was ascribed to the substitution of La by Sr modifier. Figure 3c displays the XRD patterns of the La_1-x_Sr_x_MnO_3±δ_ (0 ≤ × ≤ 1) nanoparticles heat treated at 1000 °C under static air. Higher crystallinity and well-defined symmetry were clearly observed. Similarly, the XRD reflections are shifted to smaller d-spacing as increasing the La substitution from *x* = 0.0 to *x* = 0.75 (see the inset). In the samples with *x* = 0.0 and *x* = 0.25, the maxima XRD reflections were clearly split demonstrating a structure very similar to La_0.95_Mn_0.95_O_3_ (JCPDS No. 01085-1838) with rhombohedral cell (*R3c* space group) calculated by Van Roosmalen et al. [150]. However, in the samples with *x* = 0.50, 0.75, 0.80, 0.85, and 0.90, the splitting of the maxima XRD reflections was not observed, which could be ascribed to different symmetries or different lattice parameters and same symmetry. The structural parameters of the synthesized samples in the La_1-x_Sr_x_MnO_3±δ_ (0 ≤ × ≤ 1) system were calculated by assuming a rhombohedral symmetry or cubic structure. The results showed a better fit when rhombohedral symmetry (*R3c* space group) was used for samples with 0 ≤ × ≤ 0.90. However, when the *x* value is equal to 1.0 (SrMnO_3_), another perovskite structure with hexagonal symmetry and *P6*_*3*_/*mmc* space group (194) was observed. It was found that the volume of the unit cell was decreased as increasing the *x* value, which was due to the formation of Mn^4+^ at the same time that La^3+^ (136 pm) is substitute by Sr^2+^ (144 pm) in the cationic subcell for keeping electronic neutrality. This is consistent with the ionic radius of Mn^4+^ (53 pm) being smaller than that of Mn^3+^ (65 pm), and indicates that the manganese ionic radius is actually the determinant of the unit cell volume. Moreover, it is also noticed that the appearance of Mn^4+^ ion and its content was increased with increasing the strontium content, will reduce the John–Teller effect that was favored by the Mn^3+^ cation. Therefore, the absence of the splitting of XRD peaks when the *x* values increase can be easily understood due to a higher symmetry of the structure. In order to investigate the changes of the crystallization and symmetry in milled samples (with pseudo-cubic symmetry) after annealing process (rhombohedral symmetry), XRD measurements as a function of the temperature from 30 to 1100 °C (up and down) under air atmosphere were performed. The results are shown in Fig. 4. With increasing the temperature, the crystallization process can be observed and at 1100 °C, a small diffraction peak at 2θ≈35 °C (marked with an asterisk) appear, which could be due to the formation of an orthorhombic phase [151]. As the temperature is lowered down to 800 °C, the small peak still exists and below this temperature it disappears. Below 500 °C, some reflections start to be split (see the inset) and a small peak appears before 2θ = 40 °C (marked with a cross), indicating the formation of the rhombohedral phase. The above results demonstrate that the rhombohedral phases are stable at low temperature, which can be explained in terms of oxygen composition. The orthorhombic phase is stable at high temperature (1100 °C) and its ability to accommodate the oxygen in the structure is smaller than that of the rhombohedral one, which stabilizes below 500 °C with an oxygen composition of La_0.75_Sr_0.25_MnO_3.11_. The average crystallite size (D) was calculated from X-ray line broadening of the (110) diffraction peak using the Scherrer equation, which was about 20 nm close to the data obtained from SEM and TEM images. The preparation conditions (e.g., annealing temperature and time, and synthesis methods) affect greatly the morphology and surface characteristics of rare-earth doped perovskite manganite oxide nanoparticles, as revealed by SEM and TEM [19, 61, 147, 152]. Figure 5 shows the representative SEM images of some milled and heated samples. It was observed that all the milling samples with pseudo-cubic perovskite structure had a similar microstructure characterized by aggregates of small particles. As expected, the heated samples were composed of larger faceted particles, being very similar in shape as can be seen in the H1 and H2 samples with same rhombohedral symmetry; however, the H8 sample with a hexagonal symmetry exhibited very round particles and smaller in size. The representative TEM and SAED results of the milled and heated samples are shown in Fig. 6. The TEM image of M1 sample (*x* = 0.0) (shown in Fig. 6a) had quite large particles formed in fact by agglomerated small crystallites in the nanometer size range as evidenced by the presence of rings in the SAED pattern. All the rings can be indexed in the pseudo-cubic structure (*Pm*-*3m*). TEM images of the H1 sample (Fig. 6b) and the H3 sample (Fig. 6c) also showed the presence of aggregates but formed by sub-micrometric crystallites of several hundred nanometers as observed in the enlargements of two of these crystals. The corresponding SAED patterns were taken along the [001], [211], and [210] zone axis. All the diffraction spots can be indexed in the rhombohedral structure (*R*-*3c*). The TEM image of the H8 sample (*x* = 1.0) shown in Fig. 6d displays the crystals with different sizes, and its SAED pattern taken from the [201] zone axe can be indexed in the hexagonal structure (*P6*_3_/*mmc*), matching well with the XRD data. Tian et al. [20] also synthesized a series of crystalline La_1-x_Sr_x_MnO_3_ nanoparticles with an average particle size of ~ 20 nm and good dispersion by MSS method. These La_1-x_Sr_x_MnO_3_ nanoparticles are well dispersed in water to form a clear solution and do not deposit even after standing for several weeks, exhibiting a good dispersion.

The chemical bonding and structural information of the rare earth-doped perovskite manganite oxide nanoparticles can be revealed via FTIR and Raman spectra. For example, the main absorption band around 524 cm^−1^ observed in the FTIR spectra of the La_0.7_Sr_0.3_MnO_3_ nanoparticles synthesized by a modified sol-gel route can be ascribed to the stretching vibration mode of the metal-oxygen bond in the perovskite, which involves the internal motion of a change in Mn–O–Mn bond length in MnO_6_ octahedral [153]. The strong absorption peak around 1381 cm^−1^ in La_0.7_Sr_0.3_MnO_3_ particles (annealed at 500 °C) reveals that the stretching vibration of carbonyl group (COO–) in carbonate, which diminishes with increasing calcination temperature. The La_0.7_Sr_0.3_MnO_3_ particles annealed at 800 °C have a doublet in the main absorption band around 520 cm^−1^, which should belong to stretching (3ν) and bending (4ν) modes of the internal phonon modes of MnO_6_ octahedral. The stretching mode is related to the change of Mn–O–Mn bond length and the bending mode involves the change of Mn–O–Mn bond angle. The appearance of the stretching and bending modes at transmission spectra indicates that the perovskite structure of LSMO has been formed. In the Raman spectra of Pr-doped La_0.67_Ca_0.33_MnO_3_ nanoparticles synthesized via sol-gel route, three Raman peaks around 224 cm^−1^, 425 cm^−1^, and 680 cm^−1^ are observed, respectively [39]. The Raman peak around 224 cm^−1^ can be assigned as A_g_(2), which is related to the tilting of MnO_6_ octahedron, whereas the Raman peak around 425 cm^−1^ is related to the Jahn-Teller type modes of the MnO_6_ octahedron [154]. The Raman peak around 680 cm^−1^ can be assigned as B_2g_(1), which is related to the symmetric stretching vibration mode of oxygen in MnO_6_ octahedron [154]. With increasing the Pr-doping concentration (*x*) up to *x* = 0.4, the Raman peak around 680 cm^−1^ became disappeared. That was ascribed to the increased orthorhombic distortion in the LPCMO nanoparticles with high Pr-doping concentrations, leading to the much weak symmetric stretching vibration of oxygen in MnO_6_ octahedron [39].

**Fig. 7:**
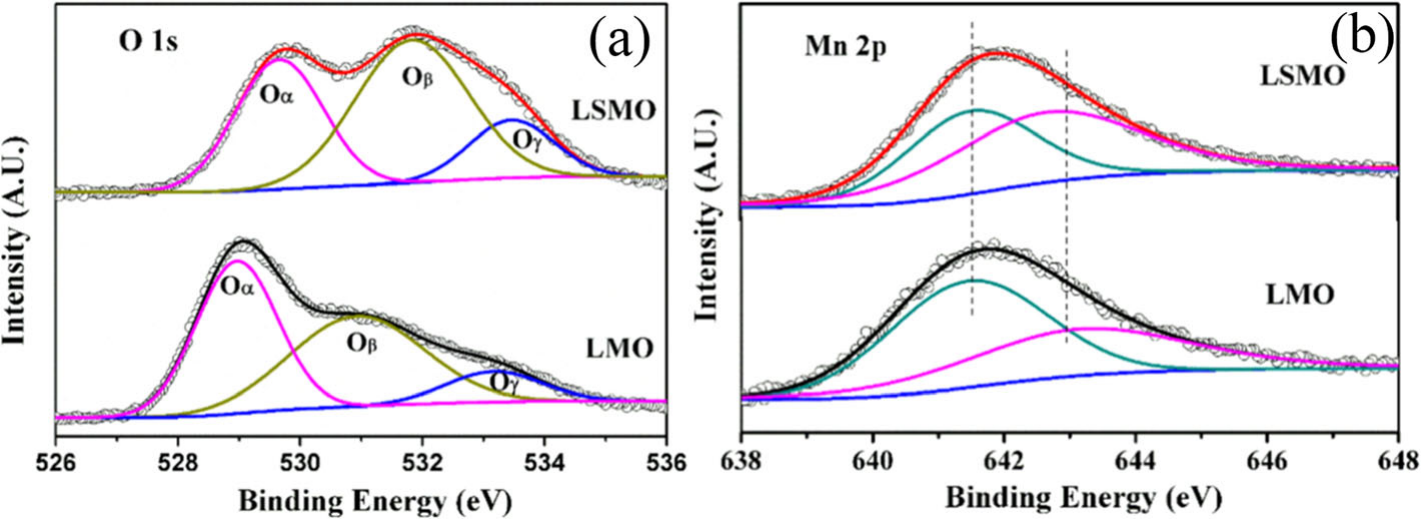
**a** Mn 2p3/2 and **b** O 1s XPS spectra of LaMnO_3.15_ (LMO) and La_0.67_Sr_0.33_MnO_2.91_ (LSMO) nanoparticles. Reproduced with permission of [21]

XPS is a surface-sensitive technique, which provides the information of the surface elemental compositions and surface chemistry of a material. The surface compositions of rare earth-doped perovskite manganite oxide nanoparticles can be identified via XPS [21, 39, 154]. For example, Fig. 7 shows the Mn 2p3/2 and O 1s XPS spectra of LaMnO_3.15_ (LMO) and La_0.67_Sr_0.33_MnO_2.91_ (LSMO) nanoparticles synthesized by MSS method, which are effective catalysts for volatile organic compounds combustion [21]. It is observed in Fig. 7a that, for each sample, an asymmetrical Mn 2p3/2 peak located at 642.2 eV could be resolved into two components with a binding energy of 641.5 eV and 642.9 eV, respectively. The former component can be assigned to the Mn^3+^ ions, whereas the latter one is assigned to the Mn^4+^ ions, indicating that the dual (Mn^4+^ and Mn^3+^) ions coexist in both samples. Quantitative analysis of the molar ratio of Mn^4+^ to Mn^3+^ on the surface of LaMnO_3.15_ was 0.72, while that of La_0.67_Sr_0.33_MnO_2.91_ was 1.33. That is to say, the average oxidation states of manganese were 3.42 and 3.57 on the surface of LaMnO_3.15_ and La_0.67_Sr_0.33_MnO_2.91_ samples, respectively. In addition, it is also noticed that the peak area of Mn^4+^ ion in the La_0.67_Sr_0.33_MnO_2.91_ sample is 35% more than that of Mn^3+^, whereas the peak area of Mn^4+^ in the LaMnO_3.15_ sample is less than that of Mn^3+^. It can be concluded that the Sr enrichment on the surface makes the Mn^3+^ ion on the surface of La_0.67_Sr_0.33_MnO_2.91_ easy to be oxidized, increasing the surface concentration of Mn^4+^. As demonstrated in Fig. 7b, the O 1s XPS peak could be decomposed into three components at binding energy (BE) equal to 529.5, 531.6, and 533.2 eV, which were ascribed to the surface lattice oxygen (O_α_), adsorbed oxygen (O_β_, such as O^−^, O^2−^, or ${\mathrm{O}}_2^{2-}$), and hydrated oxide species (O_γ_), respectively [155, 156]. Obviously, after the partial substitution of Sr^2+^ for La^3+^, the intensities of the signals of O_α_ and O_γ_ were decreased whereas the signal for O_β_ was increased, indicating an enhancement in the amount of adsorbed oxygen species. Therefore, more structural defects such as oxygen vacancies contribute to the enhanced catalytic performance of the La_0.67_Sr_0.33_MnO_2.91_ nanoparticles for toluene combustion.

### 1D Rare Earth-Doped Perovskite Manganite Oxide Nanostructures

**Fig. 8:**
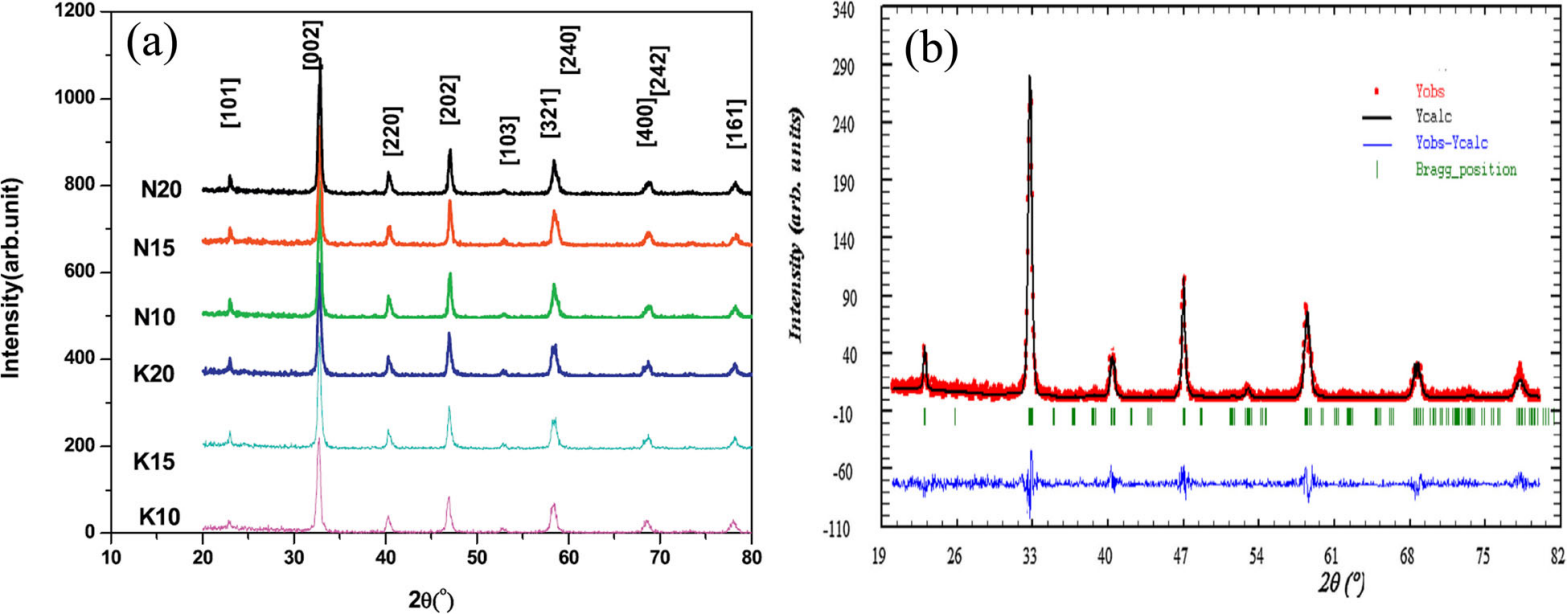
**a** Room temperature XRD patterns of La_0.7_Ca_0.3_MnO_3_ manganite nanorods synthesized via the hydrothermal method with two mineralizers namely sodium hydroxide (NaOH) and potassium hydroxide (KOH) in different alkalinity conditions (10, 15, and 20 M). **b** Room temperature XRD pattern (red symbol) and Rietveld profile (black line) for the sample N10. N (or K) means the NaOH (or KOH) mineralizer, 10 for the NaOH (or KOH) concentration. Reproduced with permission of [80]

The exciting developments in 1D perovskite manganite nanostructures must be effectively supported by a variety of structural characterization tools because the characterization provides invaluable information on the various microstructural, crystallographic, and atomic features, which can shed light on the unique properties exhibited by these fascinating materials. XRD is used for crystal structure analysis in which some structural parameters can be obtained. For example, Arabi et al. [80] synthesized the La_0.7_Ca_0.3_MnO_3_ nanorods by hydrothermal method under different conditions (e.g., different mineralization agents such as KOH and NaOH, various alkalinity conditions (10, 15, and 20 M)). Figure 8a shows the XRD patterns of the La_0.7_Ca_0.3_MnO_3_ nanorods synthesized in the presence of two different mineralization agents (KOH and NaOH) with various concentration, namely K10, K15, K20, N10, N15, and N20, respectively. It was found that all the six samples crystallized in orthorhombic structure with space group *Pnma* according to the diffraction peaks. A typical Rietveld refinement analysis of the sample N10 is displayed in Fig. 8b, indicating a good agreement between the observed and calculated profiles and no detectable secondary phase present. The FE-SEM micrographs confirmed the rod-like morphology of all the obtained samples.

**Fig. 9:**
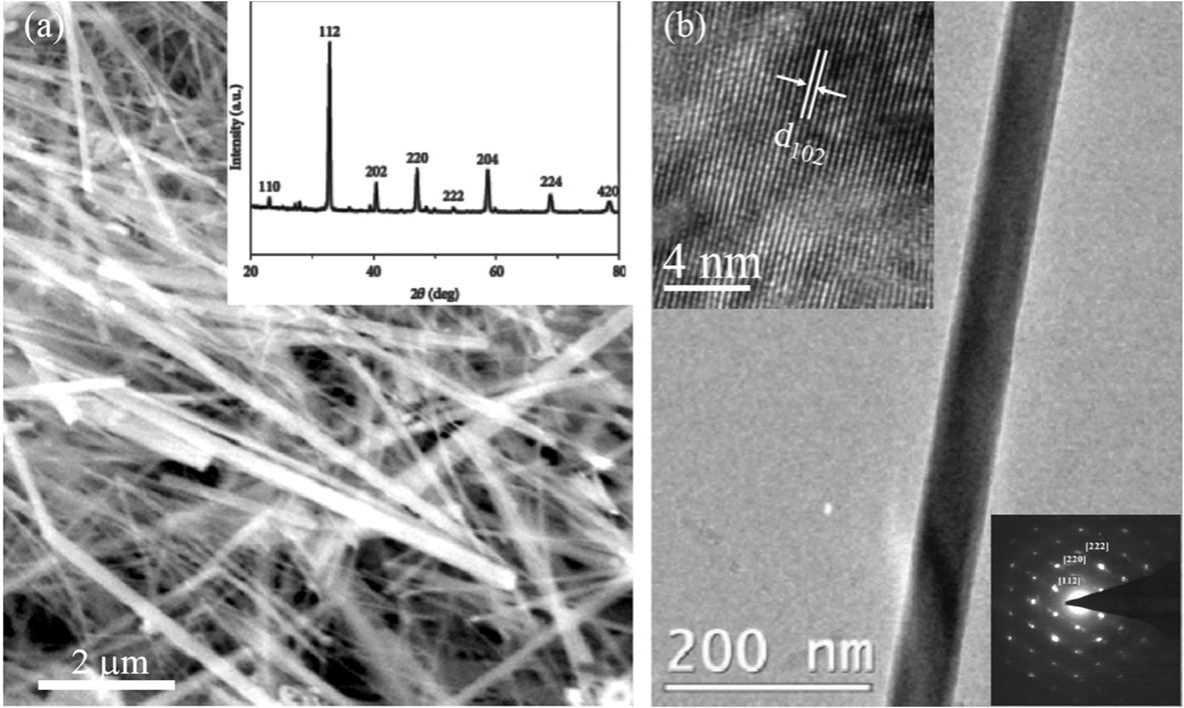
**a** SEM and **b** TEM images of the La_0.5_Sr_0.5_MnO_3_ (LSMO) nanowires synthesized by hydrothermal method. Inset in (**a**) is the XRD pattern of the LSMO nanowires, confirming the phase formation and phase purity. Insets in (**b**) are the selected area diffraction pattern and HRTEM image taken from a single LSMO nanowire, revealing the single crystalline nature of the LSMO nanowire. Reproduced with permission of [70]

Datta et al. [70] reported the template free synthesis of single-crystalline La_0.5_Sr_0.5_MnO_3_ nanowires by hydrothermal method. XRD pattern (see inset in Fig. 9a) demonstrated that these nanowires crystallized in a tetragonal structure with the space group *I*4*/mcm*. The diameter and length of these nanowires were about 20–50 nm and 1–10 μm, as revealed by SEM image (Fig. 9a) and TEM image of a single nanowire (Fig. 9b). Single-crystalline nature of the nanowires was confirmed by the SAED pattern and HRTEM image (see insets in Fig. 9b). The lattice fringes with spacing of 0.311 nm were clearly resolved in the HRTEM image, corresponding the planar distance of (102) planes. The EDS data collected from the nanowire demonstrated that the atomic percentage ratio (La:Sr):Mn:O was approximately 1:1:3, close to the desired composition. The valence state of Mn in the nanowires was also quantitatively determined by EELS, which was about 3.5, very close to its bulk value. Similar work was also carried out to determine the Mn valence in the La_0.7_Ca_0.3_MnO_3_, La_0.5_Ca_0.5_MnO_3_, and La_0.7_Sr_0.3_MnO_3_ nanowires synthesized by hydrothermal method [157]. In addition, single-crystalline perovskite manganite La_0.5_Ba_0.5_MnO_3_ and La_0.5_Sr_0.5_MnO_3_ nanowires were also synthesized by a hydrothermal method at low temperature [158]. They have a uniform width along the entire length, and their typical widths are in the range of 30–150 nm for La_0.5_Ba_0.5_MnO_3_ and 50–400 nm for La_0.5_Sr_0.5_MnO_3_. These nanowires grow along the [110] direction and their surfaces are clean without any sheathed amorphous phase. By the composite-hydroxide-mediated method, Wang et al. [159] synthesized the BaMnO_3_ nanorods with diameters of 20–50 nm and lengths of 150–250 nm, which belong to a hexagonal structure with lattice parameters of *a* = 0.5699 nm and *c* = 0.4817 nm. By template-assisted method, Li et al. [160] also synthesized the La_0.33_Pr_0.34_Ca_0.33_MnO_3_/MgO core-shell nanowires with diameters about tens of nanometers in two steps.

**Fig. 10:**
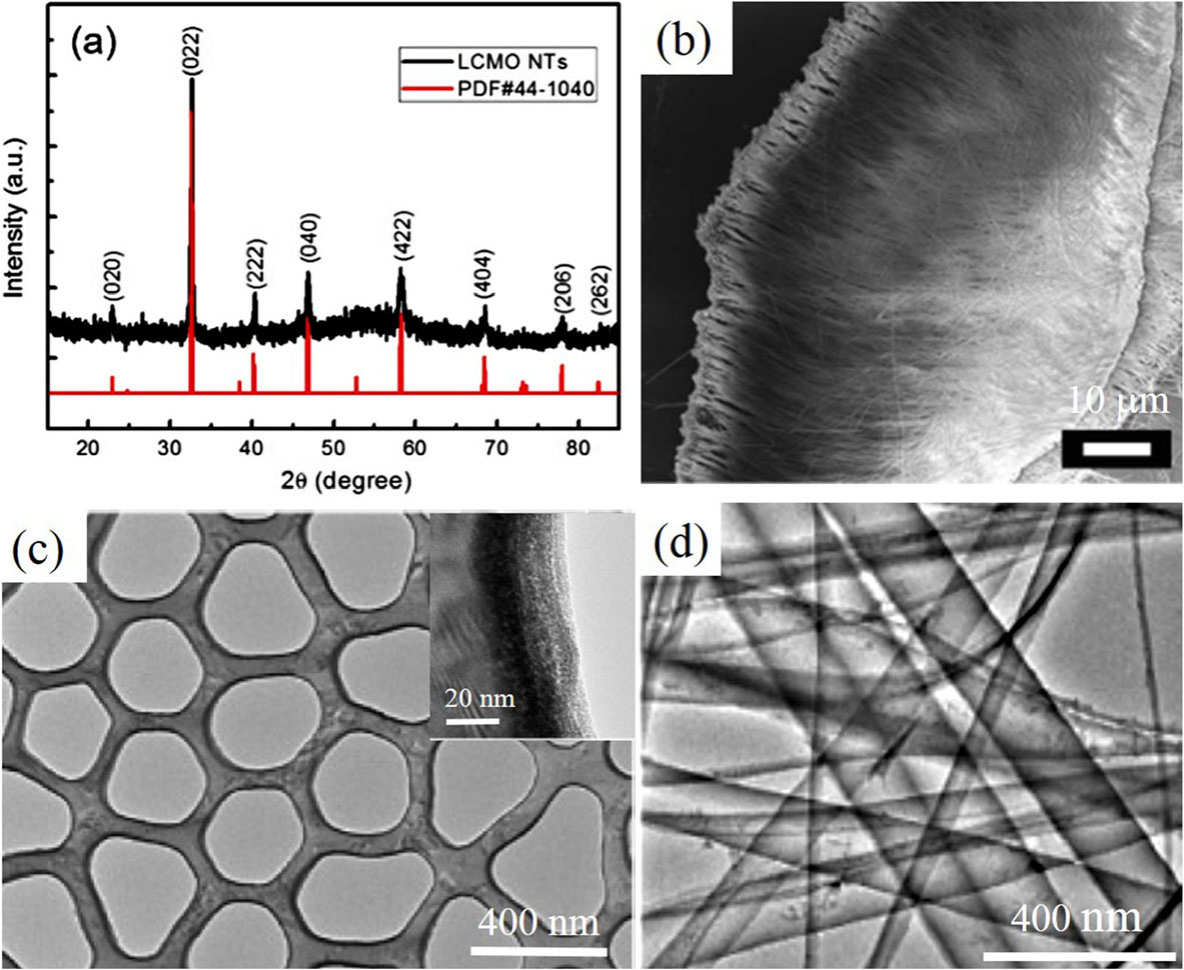
**a** XRD pattern of La_0.75_Ca_0.25_MnO_3_ (LCMO) nanotubes (NTs) prepared by template-assisted PLD method and its corresponding peak positions and intensity in JCPDS card no. 44-1040 of La_0.8_Ca_0.2_MnO_3_. **b** SEM image of the as-prepared LCMO nanotubes, and **c** cross-sectional TEM image of the LCMO nanotubes. **d** TEM images of the as-prepared LCMO nanotubes. Inset in (**c**) is enlarged local TEM image of nanotube, revealing the maximum observed wall thickness of about 20 nm. Reproduced with permission of [161]

Similarly, by using AAO membranes (pore size ~ 300 nm, thickness ~ 100 mm) as the templates, perovskite manganite La_0.75_Ca_0.25_MnO_3_ nanotubes with the average diameter of 160 nm and lengths up to tens of micrometers were fabricated by laser induced plasma filling [161]. The XRD pattern of the synthesized La_0.75_Ca_0.25_MnO_3_ nanotubes is shown in Fig. 10a, where all the diffraction peaks can be indexed perfectly to the standard monoclinic perovskite structure of bulk La_0.8_Ca_0.2_MnO_3_ (JCPDS no. 44-1040), and no second phase was detectable. That indicated well-crystallized perovskite-type phase was successfully transferred from the target to the nanotubes via the PLD method. The composition of the as-prepared La_0.75_Ca_0.25_MnO_3_ nanotubes was determined by EDS analysis technique, and the result matches well with the target. A representative SEM image of the La_0.75_Ca_0.25_MnO_3_ nanotubes array is shown in Fig. 10b, which reveals uniform fluffy feature with an average length of 50 μm. The cross-sectional TEM image of the La_0.75_Ca_0.25_MnO_3_ nanotubes is shown in Fig. 10c, where the maximum wall thickness was observed about 20 nm. This thin-walled feature determines the poor mechanical strength of the nanotubes, hence the ultrasonic processing was avoided during the nanotubes dispersion. This indicates that the length of the nanotubes can be controlled by the amount of deposition from several to tens of micrometers. It is also noticed that a nanowire-like structure with the diameter of ca. 10 nm is observed in TEM image (Fig. 10d), which may originate from either the broken walls of nanotubes or the curl of nanotubes during the annealing process. The uniformly distribution of the elements in the wall of individual nanotube was also confirmed by EDS element mapping.

### 2D Rare Earth-Doped Perovskite Manganite Oxide Nanostructures

**Fig. 11:**
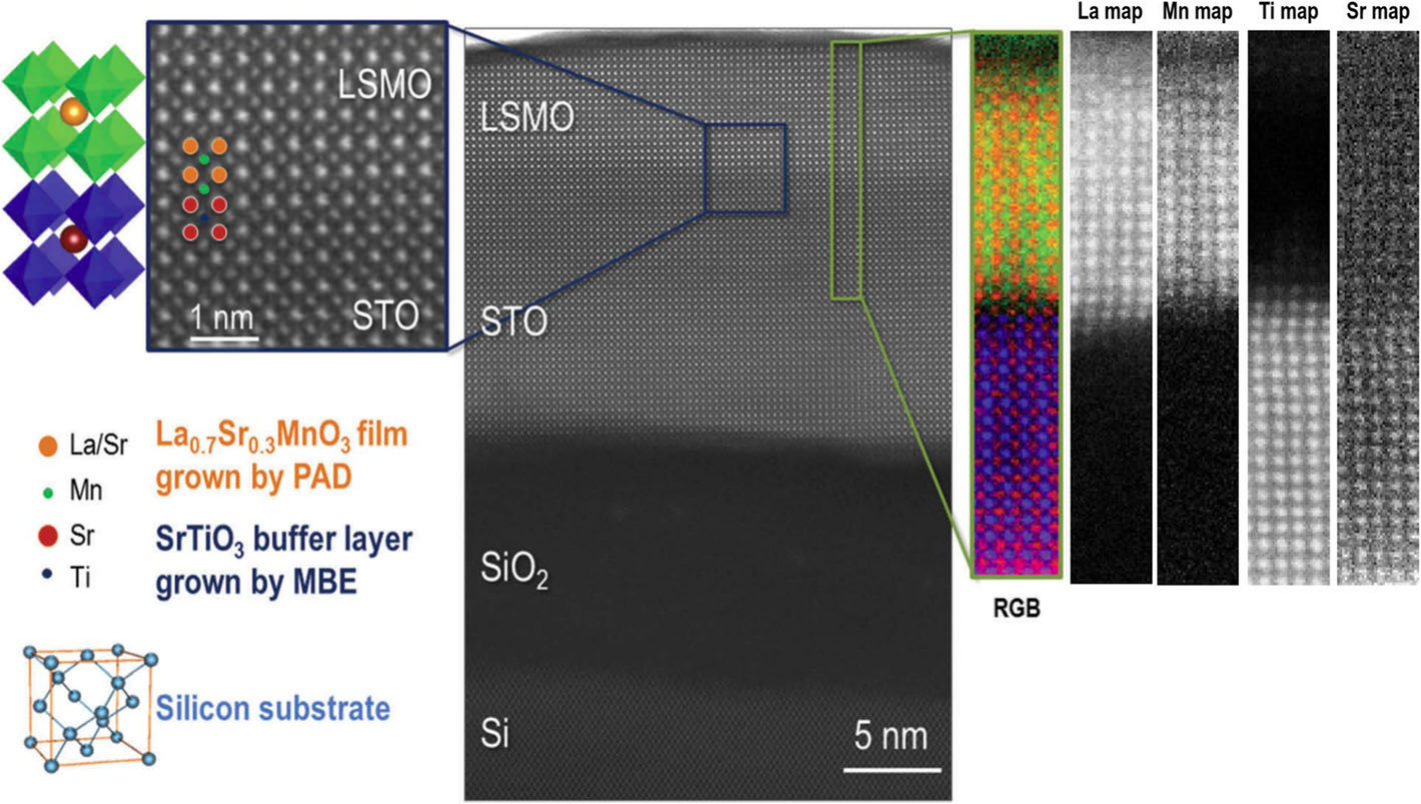
Atomic resolution Z-contrast image of epitaxial La_0.7_Sr_0.3_MnO_3_ (LSMO)_PAD_/SrTiO_3_(STO)_MBE_/Si heterostructure viewed along the [100] direction. Detail of the Z-contrast image showing the coherent interface between the LSMO and the STO/Si buffer layers (left image). EELS image (right): color elemental mapping produced by overlaying the Mn L2,3 (green), Ti L2,3 (blue), Sr M4,5 (red), and La M4,5 (orange) elemental maps, displaying a high-quality chemical interface between the STO and LSMO layer, the structure of which is sketched in the left of the image, respectively. Reproduced with permission of [183]

Perovskite manganite oxides have been a particularly appealing hunting ground for both condensed matter physics and practical device applications due to their physical properties, such as the high degree of spin polarization, CMR, spontaneous charge spin–orbital orderings, and so on [13, 162–166]. In thin films of rare earth perovskite manganites, RE_1-x_M_x_MnO_3_ (RE = rare earth, M = Ca, Sr, Ba) with mixed-valence perovskite structure, their transport properties are highly dependent upon the deposition techniques, processing conditions, and the substrate used. Among all perovskite manganite thin films, La_1-x_Sr_x_MnO_3_ has been the most widely investigated system due to its intrinsic magnetoresistance properties, electric-field tunable M-I transitions, half-metallic band structure, and the highest Currie temperature (T_c_ = 369 K for *x* = 0.33). Up to date, several deposition methods have used for their growth. For example, PLD and CVD are versatile techniques that can be used both for the growth of epitaxial and polycrystalline films [167, 168], and RF magnetron sputtering and wet chemical processes are principally for polycrystalline films [169–172]. In contrast, MBE and atomic layer deposition (ALD) are mainly used for epitaxial films and superlattice structures. For example, polycrystalline perovskite manganite La_1-x_Sr_x_MnO_3_ films with *x* = 0.15, 0.33, and 0.40 were deposited onto silicon (100) substrates by PLD in an 80/20 (Ar/O_2_) atmosphere at room temperature [173]. After deposition, the films were air annealed at 900 °C for 1 h to obtain the desired crystalline phase. Several groups have epitaxially grown the La_0.67_Sr_0.33_MnO_3_ thin films on different single-crystal substrates including STO (cubic), LAO (pseudocubic), NGO (orthorhombic), and MgO (cubic) [174–177]. Due to the small lattice mismatch between the La_0.67_Sr_0.33_MnO_3_ and these substrates (except for MgO), the La_0.67_Sr_0.33_MnO_3_ films exhibit single crystalline in a perfect epitaxy between the film and the substrate. Therefore, neither interfacial dislocations nor secondary phases’ inclusions are observed at the film/substrate interface, as it can be further identified by the cross-sectional HRTEM image [176]. Since the lattice strain is not released easily, therefore, the La_0.67_Sr_0.33_MnO_3_ films (4–60 nm) exhibit a strong perpendicular magnetic anisotropy on LAO substrate due to the biaxial compressive strain. On the other hand, the La_0.67_Sr_0.33_MnO_3_ films show the in-plane biaxial magnetic anisotropy on STO substrate by the tensile strain. The MnO_6_ octahedra of the strained La_0.67_Sr_0.33_MnO_3_ films are distorted and the hopping probabilities of e_g_ electrons are restricted. Localization of e_g_ electrons reduces the ferromagnetic interactions, enhancing the electrical resistivity of the La_0.67_Sr_0.33_MnO_3_ thin films. As for the films thicker than 60 nm, different microstructures are reported. For example, the films are still single crystalline without dislocations or intermediate layer in the whole thickness up to 120–130 nm [176, 178] or the films are divided into two regions with a strained bottom part and a relaxed top layer that are separated by an intrinsic interface containing a dislocation network [179]. These microstructures can be ascribed to strongly different growth parameters such as deposited temperature, the target-to-substrate distance, and oxygen pressure, laser influence. To make the full use of functionalities of the epitaxial La_0.67_Sr_0.33_MnO_3_ thin films into the future devices such as sensors, data storage media, and IR detectors, the integration of the functional oxides into conventional semiconductor substrates is highly essential [180, 181]. However, the direct epitaxial growth of La_0.67_Sr_0.33_MnO_3_ thin films and functional complex oxides on the Si substrates needs to be further developed due to the dissimilarities of these materials in chemical reactivity, structural parameters, and thermal stability [182]. Vila-Fungueiriño et al. [183] reported the high-quality epitaxial growth of the La_0.67_Sr_0.33_MnO_3_ thin films on Si substrates with epitaxial STO thin buffer layer by a combination of CSD and MBE methods. Figure 11 displays the STEM image of atomic and chemical structure of the epitaxial La_0.67_Sr_0.33_MnO_3_ (LSMO_PAD_)/STO_MBE_/Si heterostructure. Atomic-resolution Z-contrast images of the LSMO/STO/Si interface confirm an optimal epitaxial growth of LSMO ultra-thin films with a perfect crystalline coherence onto the STO/Si buffer layer. EELS measurements with atomic resolution (Figure 11 right) show that cationic intermixing is restricted to the first two unit cells, in agreement with the sharp contrast observed in the Z-contrast image.

**Fig. 12:**
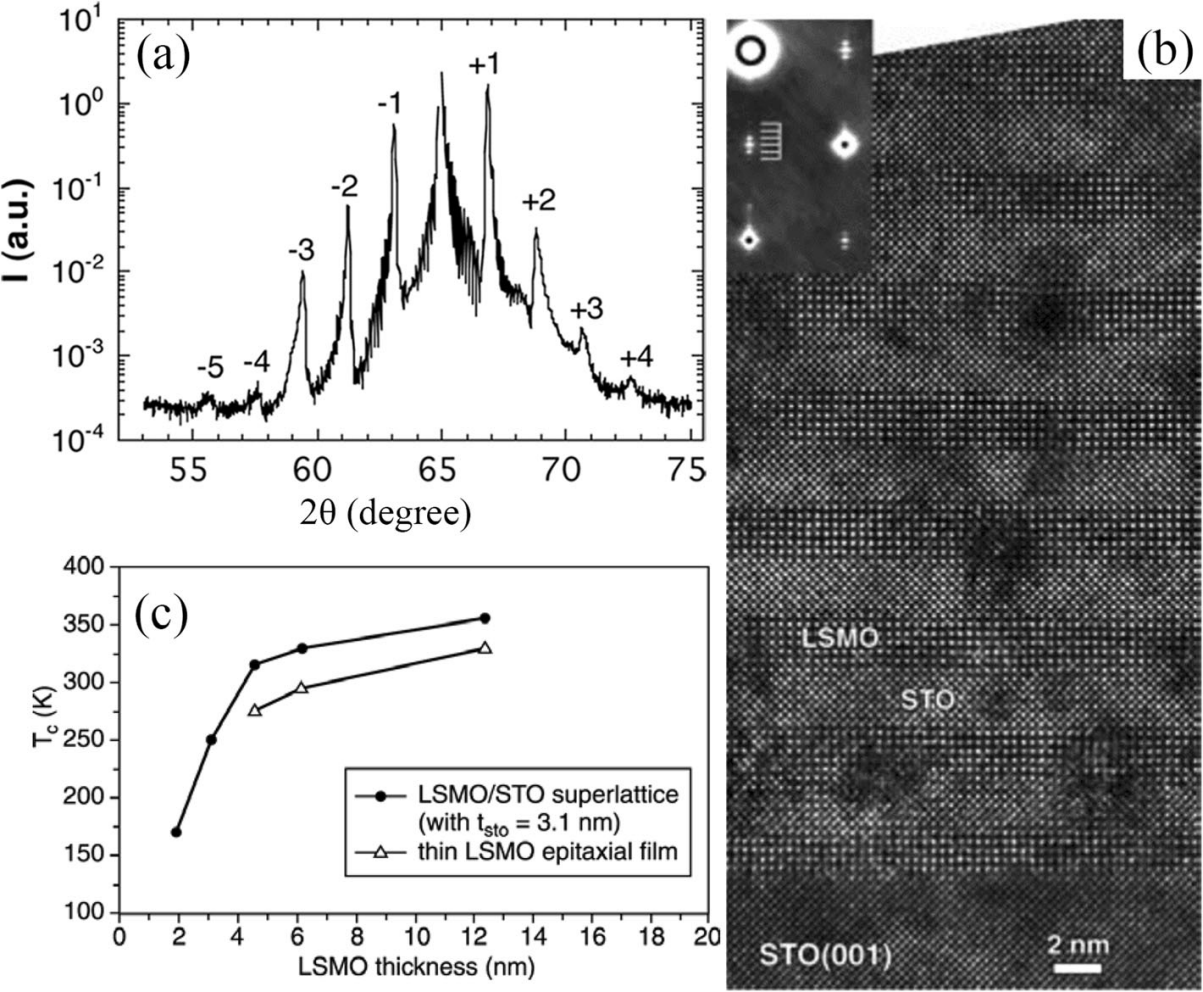
**a** XRD pattern (synchrotron) of the [(La_0.7_Sr_0.3_MnO_3_)_5_(SrTiO_3_)_8_]_15_ superlattice. **b** Cross-sectional HRTEM image of the [(La_0.7_Sr_0.3_MnO_3_)_5_(SrTiO_3_)_8_]_15_ superlattice. Reproduced with permissio n[184]. Copyright 2018, Taylor & Francis Group. **c** Dependence of Curie temperature (Tc) as a function of the thickness of the LSMO layer in [(La_0.7_Sr_0.3_MnO_3_)_m_(SrTiO_3_)_8_]_15_ superlattices grown by MOCVD. Reproduced with permission of [163]

**Fig. 13:**
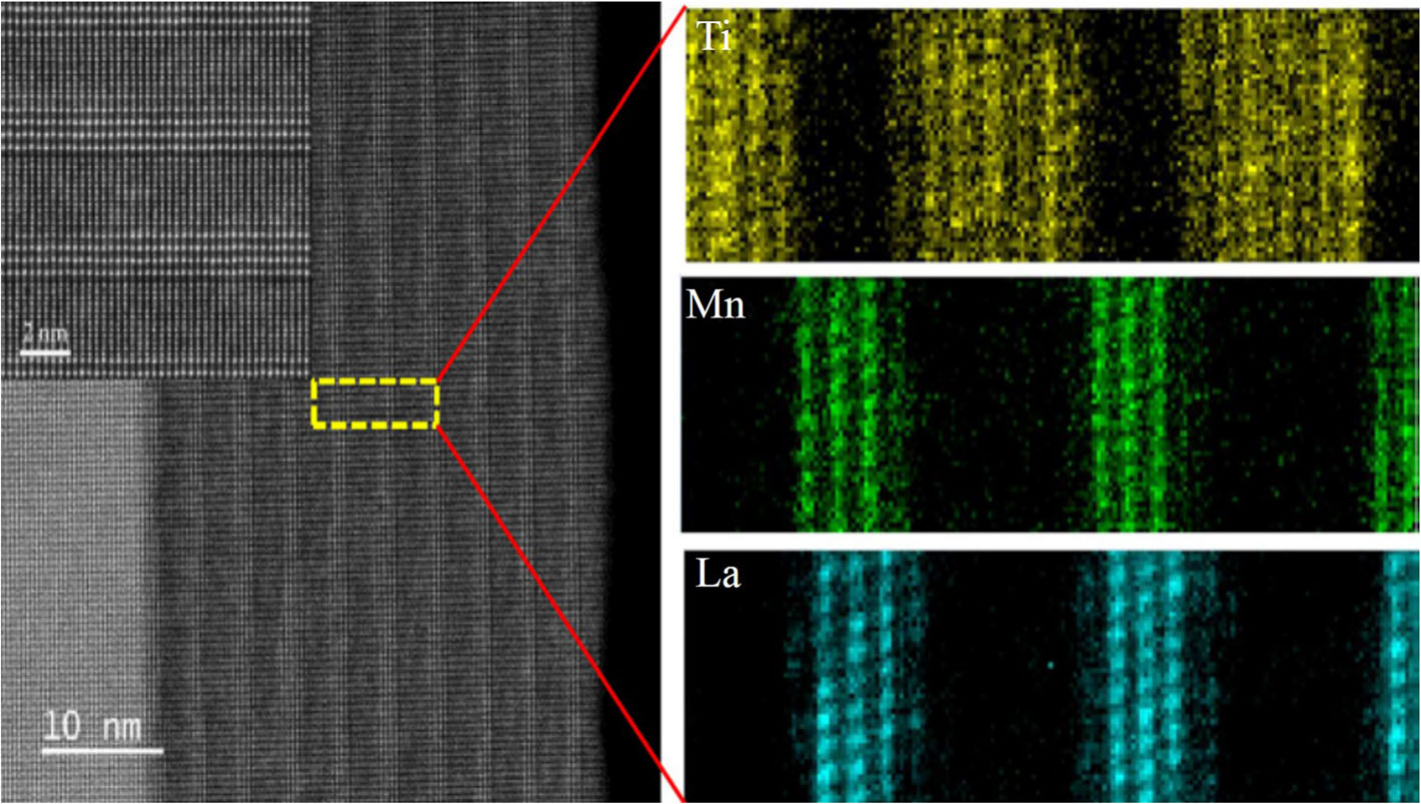
STEM image of the cross-sectional elemental mapping measured by spatially resolved electron energy loss spectroscopy for the La_0.7_Sr_0.3_MnO_3_/SrRu_0.8_Ti_0.2_O_3_ superlattice of [1.2/2.4]_10_, grown on NdGaO_3_(NGO)(001). The sample written as [m/n]_N_, where the numbers of m and n in square brackets denoting the thicknesses of the LSMO and SRTO layers in nanometers, respectively, and the subscript N representing the repeated number of LSMO layers. Reproduced with permission of [108]

To modulate the magnetic and transport properties of epitaxial La_1-x_Sr_x_MnO_3_ thin films, superlattice structures such as (LSMO/STO)_n_ were grown on LAO substrates by MOCVD [184]. The XRD pattern (synchrotron) of the (LSMO/STO)_n_ superlattice is shown in Fig. 12a, which demonstrates well-resolved satellite peaks characteristic of the superlattice period. That indicates a good coherence over the stacking. Figure 12b shows a cross-sectional HRTEM image of the (LSMO/STO)n superlattice, revealing sharp interfaces between individual layers. The perfect interfaces between adjacent layers extend on a very large scale. It is observed that the Tc value is decreased sharply when the thickness of the LSMO layer is decreased below 4 nm (see Fig. 12c) [163]. The dependence of Tc as a function of the LSMO thickness can be understood from the 2D scaling law proposed by Fisher and Barber [185], which gives a typical two-dimensional thickness of about four monolayers (*t*_2D_∼1*.*5–2 nm). Besides the (LSMO/STO)_n_ superlattice, the (La_0.7_Sr_0.3_MnO_3_ (LSMO))_m_/(SrRu_1-x_Ti_x_O_3_ (SRTO))_n_ (*x* < 0.3) superlattices was also reported by Xu et al. [108]. For clarity, the sample is written as [*m*/*n*]_N_ (the numbers of *m* and *n* in square brackets denote the thicknesses of LSMO and SRTO in nanometers, respectively, and the subscript N denotes the periods, i.e., the repeat numbers of LSMO). Figure 13 shows a STEM image of the [1.2/2.4]_10_ sample grown on NGO substrates, indicating the high quality of superlattices grown on NGO.

**Fig. 14:**
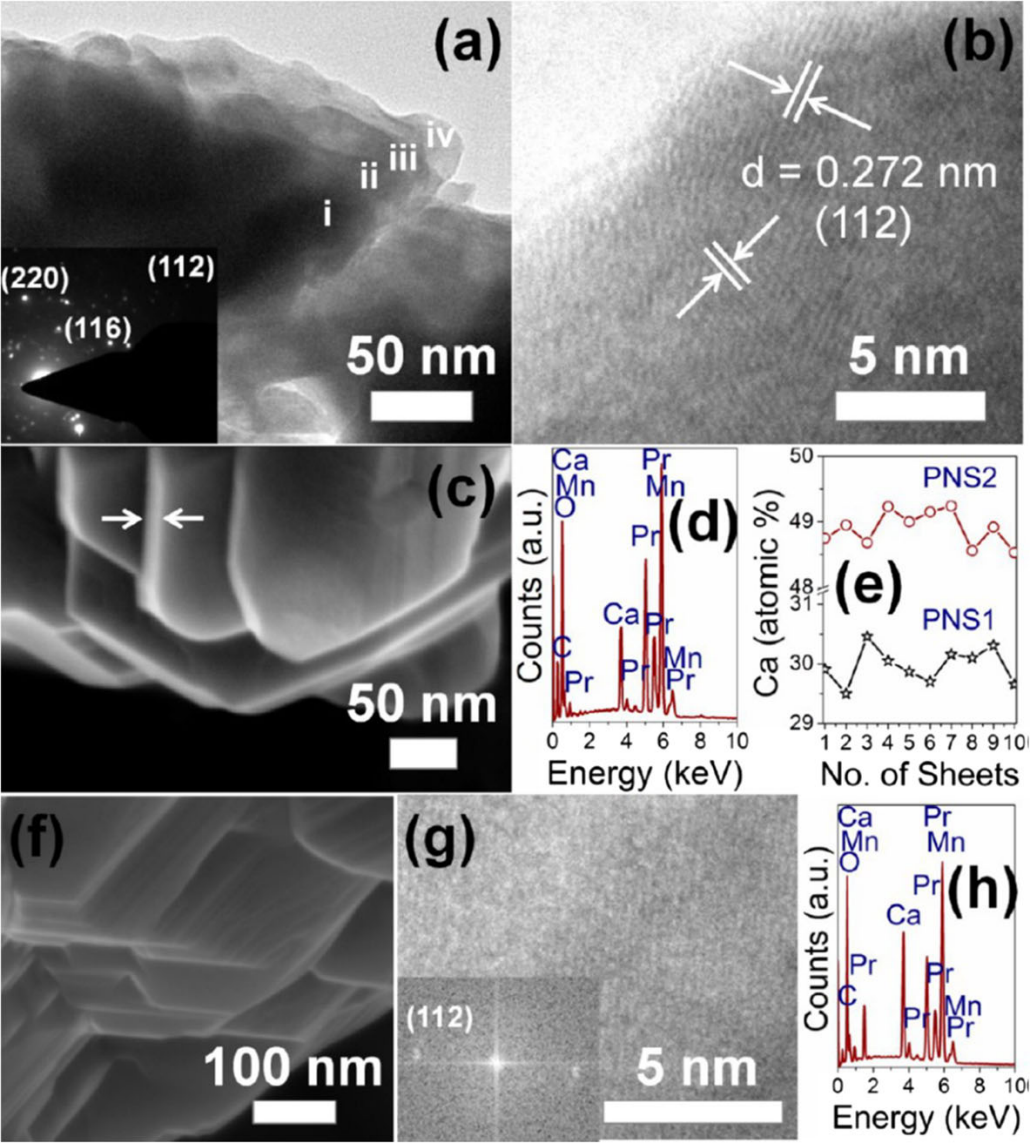
**a** TEM image of Pr_0.7_Ca_0.3_MnO_3_ nanosheet (PNS-1 sample). i–iv represent four stacked nanosheets. Inset is the SAED pattern. **b** HRTEM image of two nanosheets oriented in different directions. **c** FE-SEM image of PNS-1. Arrows indicate the thickness of the nanosheet. **d** EDAX pattern of PNS-1. **e** Homogeneity profile of Ca^2+^ doping on ten nanosheets in PNS1 and PNS2 samples. **f** FE-SEM, **g** TEM (inset: FFT), and **h** EDAX pattern of Pr_0.51_Ca_0.49_MnO_3_ nanosheet (PNS-2 sample). Reproduced with permission of [186]

The 2D morphology of perovskite manganite nanosheets have the advantage of direct implementation as potential building blocks for next-generation nanodevices due to their extended 2D network with rich electronic and magnetic properties. Recently, Sadhu and Bhattacharyya [186] synthesized pure-phase perovskite Pr_0.7_Ca_0.3_MnO_3_ manganite nanosheets (PNS1) and Pr_0.51_Ca_0.49_MnO_3_ manganite nanosheets (PNS2) via a “beakerless” pressure synthesis route. XRD Rietveld refinement patterns revealed that the PNS1 and PNS2 perovskite manganite nanosheets crystallized in the orthorhombic phase with the *Pnma* space group. The TEM image of four stacked sheets (i–iv) of the representative PNS1 sample is shown in Fig. 14a, and the SAED pattern is shown as inset in Fig. 14a, indicating the characteristic reflections of the orthorhombic phase. Lattice fringes with spacing of 0.272 nm are observed in the HRTEM image (Fig. 14b), corresponding to the (112) plane reflection. Figure 14(c) displays the FE-SEM image of PNS1 nanosheets with thickness of 10–14 nm. In addition, the nanosheet surface spans over 500–600 nm, and on average 10–12 nanosheets remain stacked together. EDS data on ten different nanosheets provided the homogeneity profile of the samples (Fig. 14d, e). The nanosheet morphology (Fig. 14f), lattice fringes with FFT (Fig. 14g), and the EDS pattern (Fig. 14h) of the PNS2 samples were found similar to that for PNS1. The EDS results matched well with that of ICP-MS as Ca^2+^/Pr^3+^ atomic ratio which was 30.0 ± 0.5 for PNS1 and 49.0 ± 0.5 atom % for PNS2, respectively.

**Fig. 15:**
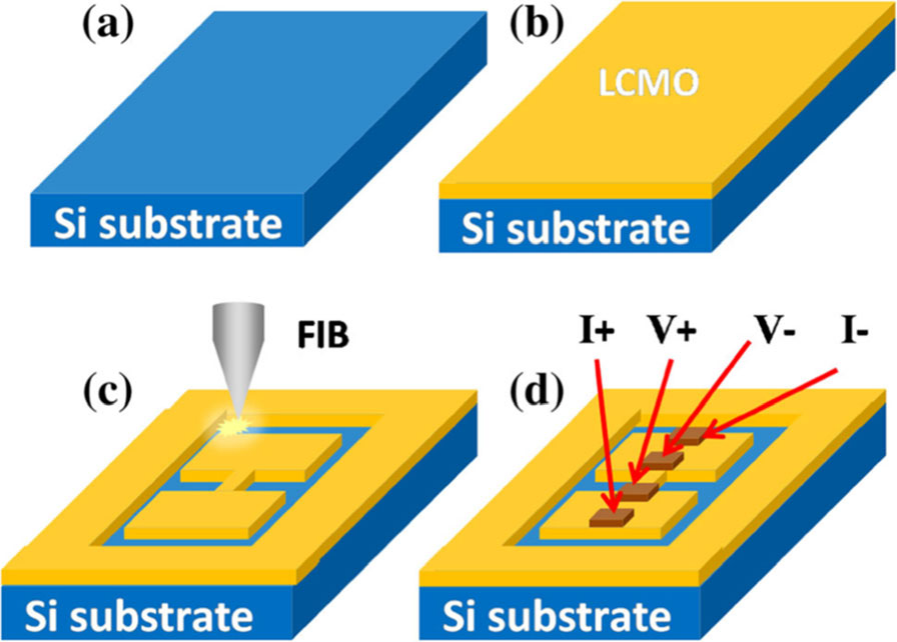
Schematic diagram for fabrication process of the La_0.67_Ca_0.33_MnO_3_ (LCMO) nanobridges. **a** Substrate, **b** LCMO film deposition, **c** nanobridge fabrication by FIB, and **d** four-electrode test configure. Reproduced with permission of [187]

2D rare earth-doped perovskite manganite oxide nanostructures such as La_0.67_Ca_0.33_MnO_3_ nanobridges are also fabricated by FIB method from the epitaxial La_0.67_Ca_0.33_MnO_3_ thin films [187]. Figure 15 shows schematic diagrams for the fabrication process of La_0.67_Ca_0.33_MnO_3_ nanobridges, which involves epitaxial growth of La_0.67_Ca_0.33_MnO_3_ film by PLD method or MOCVD, nanobridge fabrication by FIB (or EBL), and four-electrode construction for physical measurement. For physical property measurements, the electric connection between the sample and instrument can be achieved in different ways such as wire bonding, indium and silver paint [188].

### 3D Rare Earth-Doped Perovskite Manganite Oxide Nanostructures

**Fig. 16:**
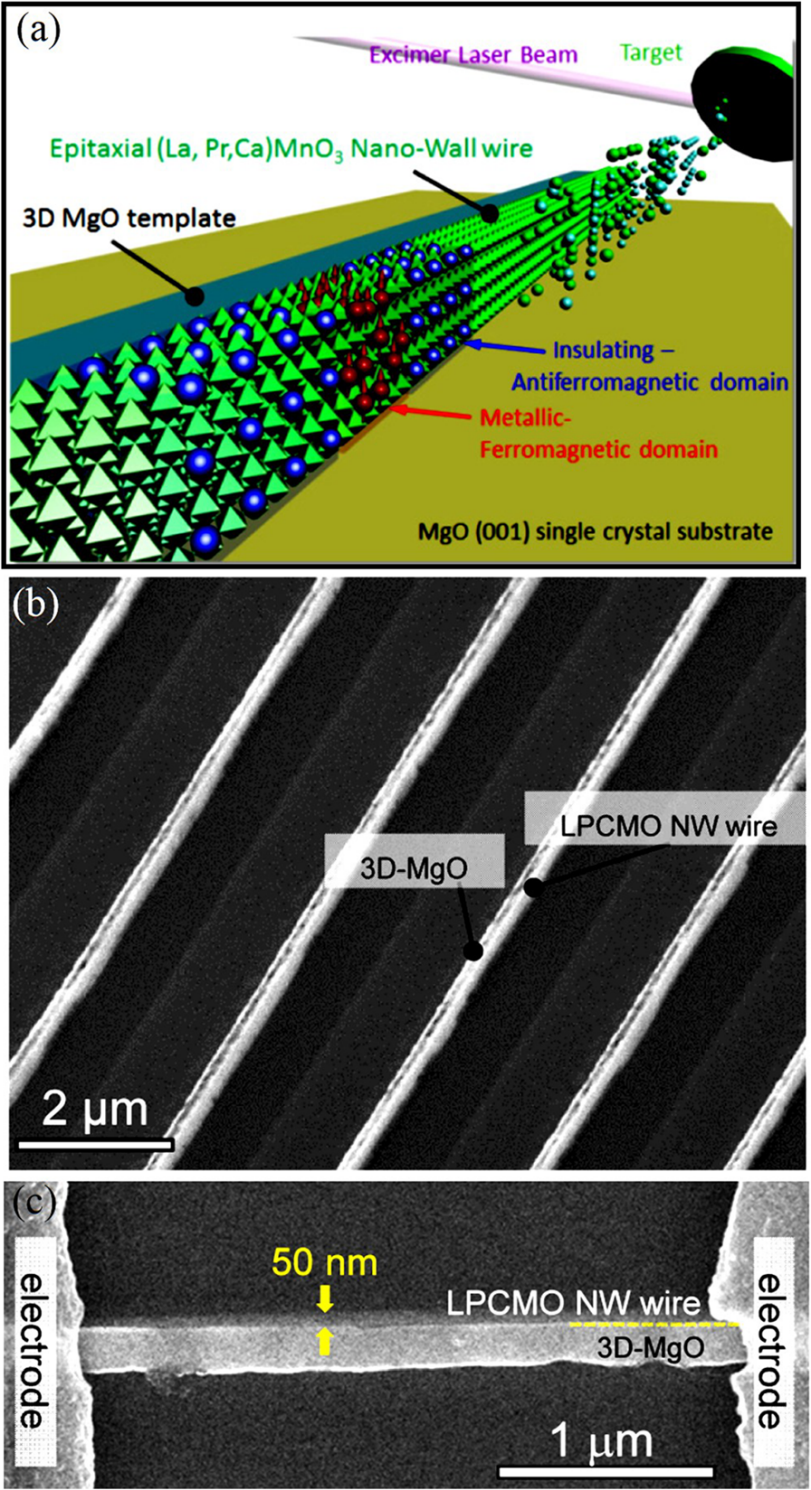
**a** Concept of an original nanofabrication technique, 3D nanotemplate PLD, where (La_0.275_Pr_0.35_Ca_0.375_)MnO_3_ (LPCMO) is deposited onto the side-surface of 3D MgO using inclined PLD. **b** SEM image of the LPCMO nanowires with 50 nm width on a MgO (001) single crystal substrate. **c** Top-view SEM image of an LPCMO nanowire structure bridging electrodes with a 2 μm gap. Reproduced with permission of [192]

Generally, 3D nanostructures can be fabricated via “top-down” and “bottom-up” approaches. For example, well-defined 3D epitaxial perovskite manganite oxide nanowall wires are prepared by using a unique nanofabrication technique combining of top-down nanolithography and bottom-up epitaxial thin-film growth [189–191]. A schematic diagram for growing such 3D nanostructures is shown in Fig. 16a, where (La_0.275_Pr_0.35_Ca_0.375_)MnO_3_ is deposited onto the side-surface of 3D MgO using inclined PLD [192]. Figure 16b demonstrates a SEM image of (La_0.275_Pr_0.35_Ca_0.375_)MnO_3_ nanowall wires with 50 nm width on a MgO(001) single crystal substrate, where well-defined (La_0.275_Pr_0.35_Ca_0.375_)MnO_3_ nanowall wires with homogeneous lateral interfaces between each nanowire and the side-surface of the 3D-MgO nanotemplate, are realized. A single (La_0.275_Pr_0.35_Ca_0.375_)MnO_3_ nano-wall wire bridging two electrodes fabricated as a two probe device using photolithography is shown in Fig. 16c. The realization of nanowall-wire samples of strongly correlated perovskite manganite materials enables ones to capture single electronic domains when the width of the nanowall-wire approaching to a single electronic domain and to identify their M–I transition characteristics at the single domain scale. In addition, (La_0.275_Pr_0.35_Ca_0.375_)MnO_3_ nanowall-box are also grown by the same method [145].

**Fig. 17:**
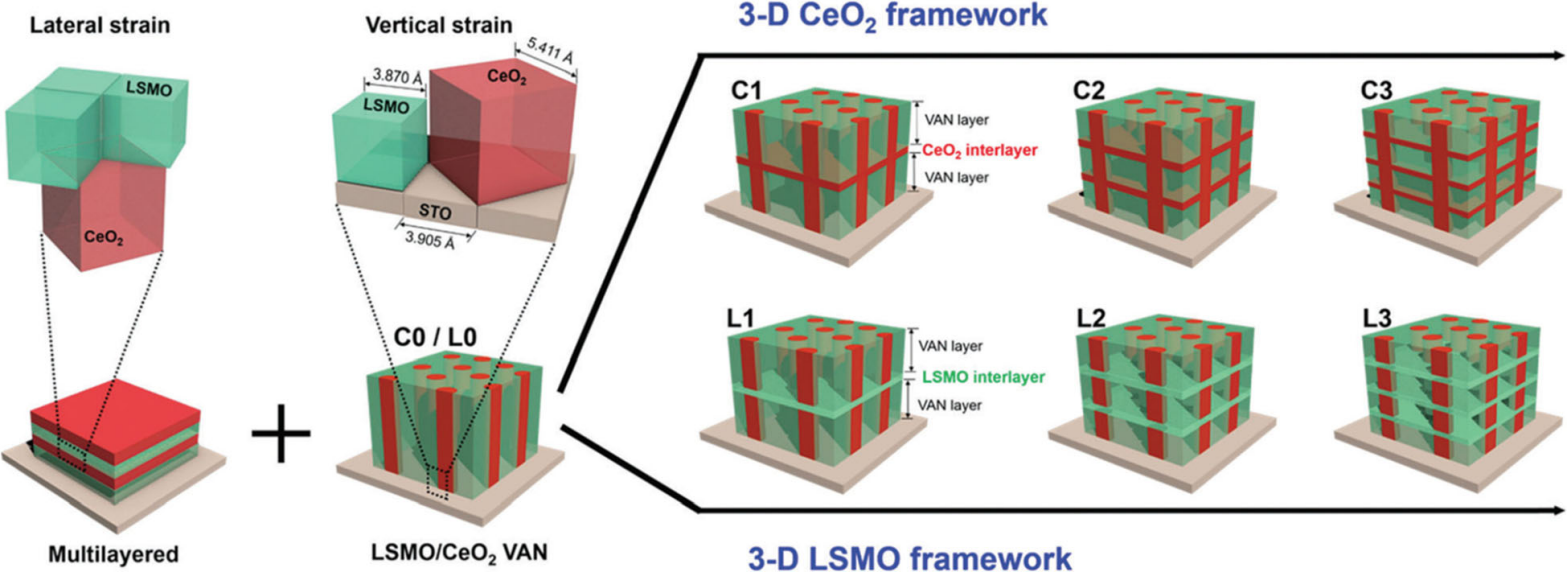
Schematic illustration of 2-phase heterogeneous microstructure evolution of the thin films: from vertical aligned nanocomposite (VAN) C0/L0 to 3D CeO_2_ framed thin films C1–C3 and 3D La_0.7_Sr_0.3_MnO_3_ (LSMO) framed thin films L1–L3. The 3D framed microstructure is achieved by alternative growth of the single phase and the VANs in multilayered fashion. This design combines the lateral strain introduced from multilayered thin film and the vertical strain from interfacial coupling in VANs, creates 3D interconnected CeO_2_ or LSMO framework microstructures within the thin films, and provides a versatile tool to achieve 3D strain tuning. The unit cells and phase of LSMO are in green, and the unit cells and phase of CeO_2_ are in red. The single layer LSMO–CeO_2_ VAN thin films are named as C0 or L0, without LSMO or CeO_2_ as the interlayers. 3D CeO2 interlayered thin films with 1, 2, and 3 interlayers inserted in VAN structures are named as samples C1, C2, and C3, respectively. Similarly, 3D LSMO interlayered thin films with 1, 2, and 3 interlayers inserted in VAN are named as sample L1, L2, and L3, respectively. Reproduced with permission of [198]

**Fig. 18:**
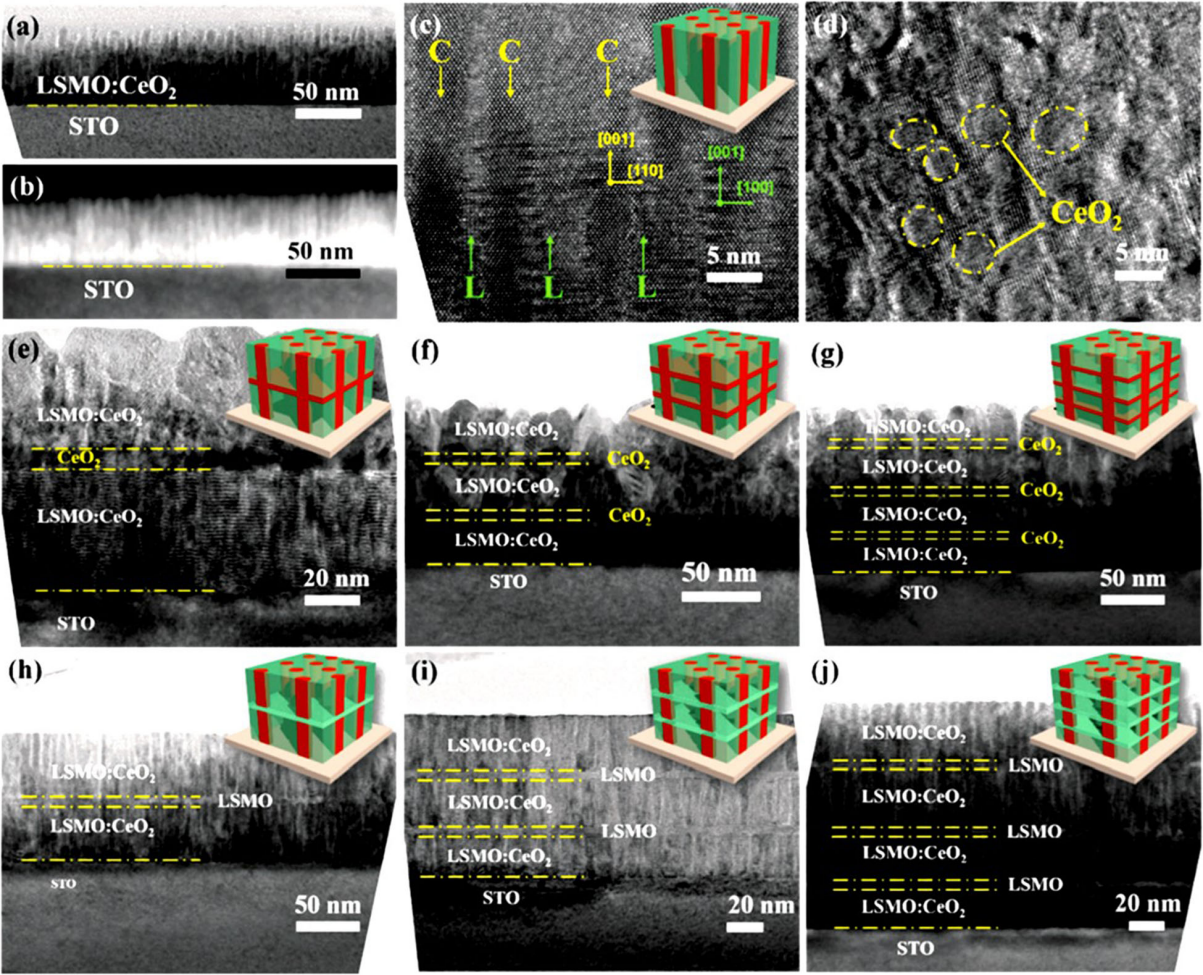
**a** Cross-sectional TEM image of the VAN thin film C0 and **b** its corresponding STEM image at low magnification. **c** Cross-sectional and **d** plan-view HRTEM images of sample C0. In the HRTEM image of (**c**), “C” in yellow points out the CeO_2_ nanopillars and “L” in green points out the La_0.7_Sr_0.3_MnO_3_ (LSMO) matrix. Clearly, those CeO_2_ nanopillars with a large aspect ratio are vertically aligned and well distributed in the LSMO matrix and the sharp phase boundaries suggest the well separated growth of the two phases. Cross-sectional TEM images of the thin films **e**–**g** C1–C3 and **h**–**j** L1–L3, showing the microstructures of 3D interconnected CeO_2_ and LSMO frames embedded within the thin films respectively. Reproduced with permission of [198]

3D nanostructured perovskite manganite materials such as branched nanorods or nanoforests have attracted extensive research attentions due to their unique 3D nature. By making full use of the vertical and horizontal dimensions, perovskite manganite oxide 3D nanostructures exhibit many fascinating physical and chemical properties due to their highly enhanced interfacial area and stability, as compared to the one-dimensional (1D) nanowire arrays [193–197]. Recently, 3D nanostructures have been successfully constructed by interlayering La_0.67_Ca_0.33_MnO_3_ (LCMO)–CeO_2_-based epitaxial vertically aligned nanocomposite thin films with pure CeO_2_ (or LSMO) layers, which were epitaxial grown on SrTiO_3_ (001) substrates via a PLD method [198]. This 3D strained framework structures combine both the lateral strain by the layered structures and the vertical strain in the vertically aligned nanocomposite and thus achieve the maximized strain tuning in LSMO. The schematic diagram illustrating the design of such 3D nanostructures is shown in Fig. 17, which creates 3D interconnected CeO_2_ or LSMO framework microstructures within the thin films, and provides versatile tool to achieve 3D strain tuning. The structural characterizations of this 3D nanostructures are shown in Fig. 18 [198]. Clearly, the CeO_2_ nanopillars with a large aspect ratio are vertically aligned and well distributed in the LSMO matrix and the sharp phase boundaries suggest the well separated growth of the two phases. Thus, a well-defined 3D interconnected LSMO frame is clearly achieved within the dense films. More importantly, by varying the types of the interlayers (e.g., CeO_2_ or LSMO) and the number of interlayers from 1 to 3 layers, such 3D framework nanostructures effectively tune the electrical transport properties of LSMO, e.g., from a 3D insulating CeO_2_ framework with integrated magnetic tunnel junction structures, to a 3D conducting LSMO framework, where the MR peak values have been tuned systematically to a record high of 66% at 56 K and enhanced MR properties at temperatures above room temperature (~ 325 K). This new 3D-framed design provides a novel approach in maximizing film strain, enhancing strain-driven functionalities, and manipulating the electrical transport properties effectively.

## Physical Properties of Rare Earth-Doped Perovskite Manganite Oxide Nanostructures

### Rare Earth-Doped Perovskite Manganite Oxide Nanoparticles

#### Magnetic Properties

Superconducting quantum interference device (SQUID) magnetometer is the most powerful, sensitive, and widely used instrument for magnetic characterization in material science. This device works on the principal of quantum interference produced using Josephson junctions. This measurement system is used for d.c. magnetization and M vs. H measurements of the samples. For d.c. magnetization, a small external field is applied and χ is measured as a function of temperature at constant applied field. For M-H measurements, magnetization is measured at a constant temperature while magnetic field is varied up to a certain value of positive and negative applied field. The most common units for the magnetic moment is emu. The natural unit of the magnetization is thus emu/g or emu/cm^3^. If one can estimate the number of atom in the sample, then one can also calculated the magnetic moment per atom in μ_B_.

**Fig. 19:**
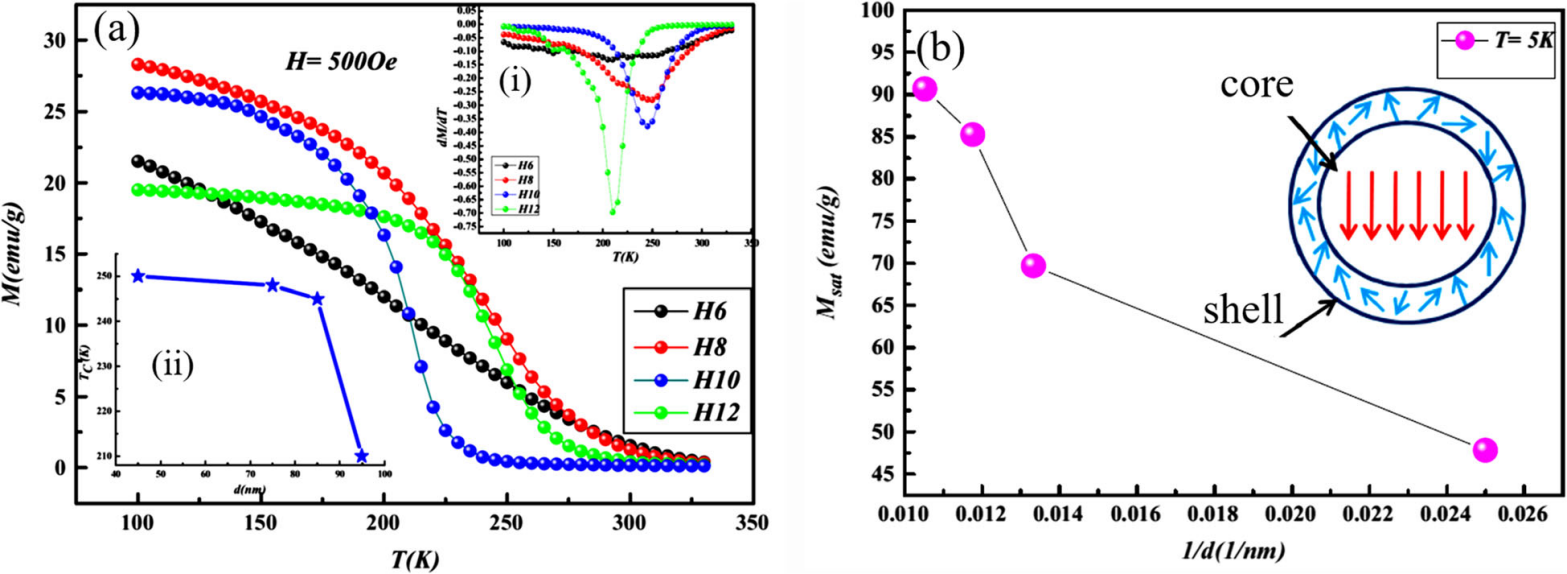
**a** Temperature dependence of magnetization in the La_0.9_Sr_0.1_MnO_3_ samples. Insets: (i) the dM/dT vs. T plots, and (ii) dependence of T_C_ on the average of grain size (*d*). **b** Saturation magnetization M_sat_ values versus the inverse of the crystallite sizes (1/*d*). Insert in (**b**) is a schematic diagram of the core-shell model for ferromagnetic nanoparticle. Reproduced with permission [148]. Copyright 2014, Elsevier Ltd and Techna Group S.r.l

The perovskite manganite oxide nanoparticles of La_1-x_Sr_x_MnO_3_ are one of the most attractive rare earth-doped perovskite manganites, which exhibit a metallic nature, large bandwidth, and the Curie temperature (*T*_C_) as high as 300–370 K [199]. Their magnetic properties are influenced by many factors; the key ones include chemical compositions, the type and the degree of defectiveness of the crystal lattice, the particle size and morphology, the interactions between the particle, and the surrounding matrix and/or the neighboring particles. By changing the nanoparticle size, shape, composition ,and structure, one can control to an extent the magnetic characteristics of the nanoparticles. For example, Baaziz et al. [148] synthesized La_0.9_Sr_0.1_MnO_3_ nanoparticles by the citrate-gel method and annealed them at 600 °C (H6), 800 °C (H8), 1000 °C (H10), and 1200 °C (H12), respectively. Their magnetization (M) versus temperature (T) curves measured under the applied magnetic field of 500 Oe are shown in Fig. 19a. The *T*_C_ was obtained from the inflection points in dM/dT as a function of temperature for all samples in the inset (Fig. 19i)). As shown in M–T curve, all samples exhibit a PM to FM transition at *T*_C_ upon cooling. The *T*_C_ dependent upon the particle sizes (*d*) is shown as an inset (Fig. 19ii) in Fig. 19a, where it is clearly observed that *T*_C_ decreases from 250 to 210 K with increasing the particle size from 45 to 95 nm. The decrease of the Curie temperature with increasing the particle size can be ascribed to the strain effects of grains induced by the distortion at grain boundaries and the orthorhombic strains caused by the strong J–T coupling. It was also found that the saturation magnetizations (M_sat_) of the La_0.9_Sr_0.1_MnO_3_ nanoparticles were increased with increasing the particle sizes, as shown in Fig. 19b. The reduction of M_sat_ in the small particles may be attributed to the loss of long-range ferromagnetic (FM) order in the smaller particle sized samples since the surface contribution is larger in this case. This can be explained in terms of a core-shell model developed for nanoparticles [58, 200, 201], where ideally the core part retains the bulk-like physical properties, but the outer shell (with thickness *t*) can be considered as a disordered magnetic system whose magnetization may be considered to be zero in the absence of the magnetic field (see the inset of Fig. 19b). This shell is named as the dead layer, which does not have any spontaneous magnetization. As the particle size decreases, the shell thickness *t* increases, which enhances the inter-core separation between two neighboring particles, resulting in a decrease in the magnetic exchange energy. That is the reason why the reduction in the saturation magnetization with decreasing the particle size. In order to confirm this, M_sat_ value versus the inverse of the crystallite sizes (1/*d*) is plotted in Fig. 19b, which reveals a quasi-linear relationship between the M_sat_ and 1/*d*. Similarly, the particle size effect on the magnetic properties of La_0.8_Sr_0.2_MnO_3_ nanoparticles synthesized by the microwave irradiation process was also observed [64].

**Fig. 20:**
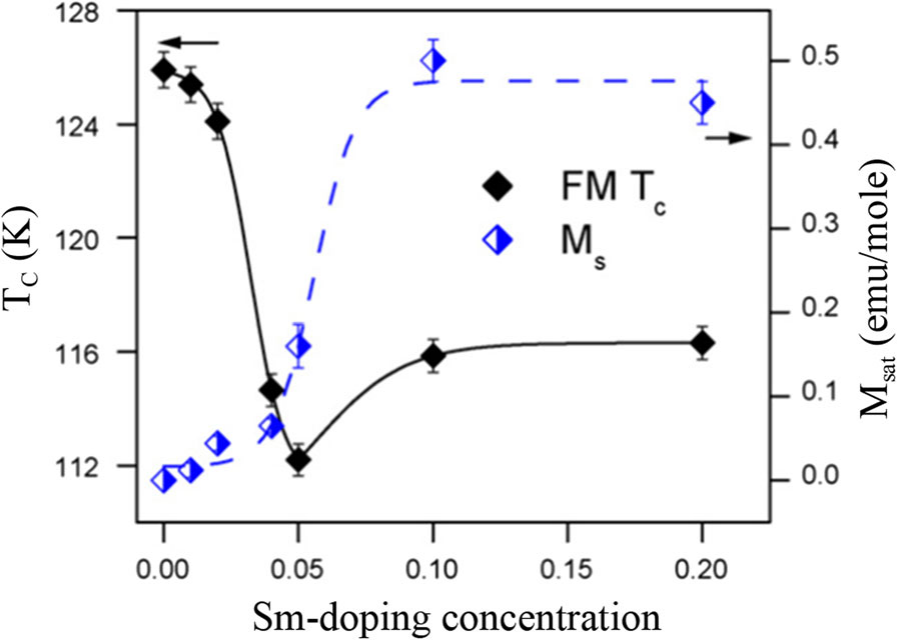
Variation in saturation magnetization (*M*_sat_) and magnetic transition temperature (*T*_C_) of the Ca_1-*x*_Sm_*x*_MnO_3_ nanopowders as a function of the Sm-doping concentrations. Solid and broken lines just show the trends. Reproduced with permission of [202]

Beside the particle size and the synthesis process, the magnetic properties of La_1-x_Sr_x_MnO_3_ nanoparticles are also dependent upon the Sr-doping level. Tian et al. [20] synthesized La_1-x_Sr_x_MnO_3_ (*x* = 0, 0.3, 0.5, 0.7) nanoparticles by a facile molten salt synthetic route. Their magnetic properties were modified by the Sr-doping levels. The Curie temperatures (Tc) deduced from the magnetization (*M*)-temperature (*T*) curves were 208, 252, 257, and 275K for *x* = 0, 0.3, 0.5, and 0.7, indicating that the Tc values were increased with the Sr-doping levels. Recently, Xia et al. [39] synthesized (La_1-x_Pr_x_)_0.67_Ca_0.33_MnO_3_ (LPCMO, *x* = 0.0–0.5) nanoparticles via sol-gel process. Their *T*_C_ values were measured to be 233, 228, 180, and 171 K for the LPCMO samples (*x* = 0.1, 0.2, 0.3, and 0.4), respectively, which were decreased with increasing the Pr-doping concentration. That is ascribed to that the double-exchange interactions in the LPCMO nanoparticles became weakened due to the narrower bandwidth and the reduced mobility of e_g_ electrons. Similarly, in the La_1-x_Ba_x_MnO_3_ (*x* = 0.3, 0.5, and 0.6) nanocubes synthesized via hydrothermal methods, the low-temperature saturation magnetization was also decreased with increasing the Ba-doped content [46]. However, in the Ca_1-x_Sm_x_MnO_3_ (CSM, *x* = 0.0–0.20) nanoparticles, the *T*_C_ value was first abruptly decreased with increasing the Sm-doping concentration up to 0.05, but with a further increase in the doping level, it has monotonically augmented and approached a plateau above *x* = 0.1, as shown in Fig. 20 [202]. The decrease in magnetic transition temperature demonstrates that the strength of the super-exchange interaction is reduced due to the dilution of the Mn^4+^ lattice by Mn^3+^ spins. While at moderate and larger doping regime, the magnetic behavior of nanograins are dominant by double exchange Mn^3+^–O–Mn^4+^ interactions and the strong inter/intragrain coupling. The M_sat_ was also increased linearly from 0 to 0.03, and then it was increased abruptly in the 0.03–0.05 doping level, approaching a plateau above *x* = 0.1 (seen in Fig. 20). Besides, as a popular system, Ca-doped lanthanum manganite, especially the magnetic properties of Ca-doped lanthanum manganite nanoparticles with different Ca doping levels were investigated by several groups [203–206]. The reduction of the M_sat_ due to the particle size reduction is attributed to the increase of magnetic dead layers. With decreasing the particle size, the finite-size effect causes the decrease of *T*_C_.

**Fig. 21:**
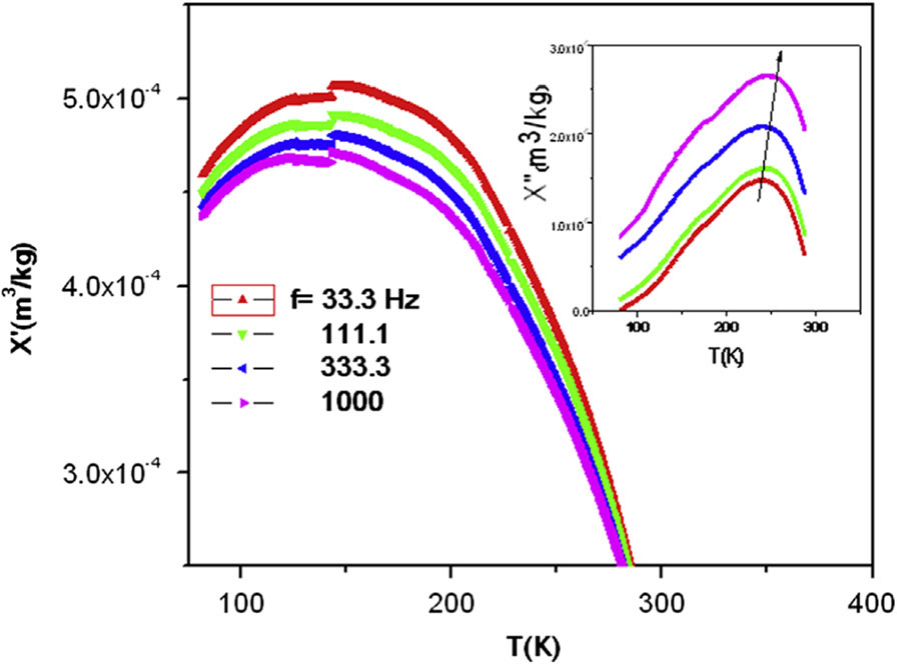
Ac susceptibility versus temperature for the La_0.8_Sr_0.2_MnO_3_ sample (particle size of 20 nm) at different frequencies. Inset is the imaginary part as a function of the temperature at different frequencies. Reproduced with permission of [64]

To obtain information on the dynamical properties of magnetic nanoparticles, ac magnetic susceptibility is measured on cooling or heating the nanoparticle samples. The ac susceptibility (χ_ac_) has two components: one is in-phase (χ') with the excitation while the other is a dissipative out of phase (χ") component. Figure 21 shows the ac susceptibility of the La_0.8_Sr_0.2_MnO_3_ nanoparticles (with particle size of 20 nm) versus temperature at an applied magnetic field of 1 mT and different frequencies (33.3, 111, 333.3, and 1000 Hz) [64]. In χ′(T) and χ″(T), a frequency-dependent peak near T_b_ = 237 K (blocking/freezing temperature) was observed, which shifted to a higher temperature as increasing the frequency. The frequency dependence of the ac magnetic susceptibility and appearance of the irreversibility temperature in the field cooling (FC) and zero-field cooling (ZFC) magnetization patterns are the signature for super-paramagnetic/spin glass (SPM/SG) regime in both the interacting and non-interacting nanoparticles [207, 208]. Similar phenomenon was also reported for other perovskite manganite nanoparticles such as spin glass or super-spin glass behavior in La_0.67_Sr_0.33_MnO_3_ [209] and La_0.6_Sr_0.4_MnO_3_ nanoparticles [210], and SPM behavior in La_2/3_Sr_1/3_MnO_3_ nanoparticles [211]. To reveal the dynamic behavior of magnetic nanoparticles and the nature of the T_b_ peak (SPM or SG) in of the La_0.8_Sr_0.2_MnO_3_ nanoparticles calcined at 600 °C for 3 h, three well-known phenomenological models (e.g., Neel-Brown model, Vogel-Fulcher model, and critical slowing down model) have been used to fit the experimental data of ac susceptibility of the sample. The best fitting results from the critical slowing down model indicate that there exists a strong interaction between the LSMO magnetic nanoparticles. However, in the La_0.67_Sr_0.33_MnO_3_ nanoparticles (with average particle size of 16 nm) prepared by sol-gel method, Rostamnejadi et al. [212] found that the experimental data of ac susceptibility was best fitted by the Vogel-Fulcher model, whereas the fitting the experimental data with Neel-Brown model and critical slowing down model give out unphysical value for the relaxation time. In addition, the unusually large value for the dynamic critical exponent and smaller value for relaxation time constant obtained from the fitting of data by critical slowing down model indicate that the spin-glass phase transition does not take place in this system of nanoparticles.

#### Magnetocaloric properties

Recently, the large magnetocaloric effect (MCE) in perovskite manganites has been widely studied [213, 214]. MCE originates from the heating or the cooling of magnetic material due to the application of magnetic field, which is characterized by the magnetic entropy change. The magnetic entropy change (ΔS) can be estimated from the M(H) curves and the use of the Maxwell’s relation

1 $$ \Delta  {\mathrm{S}}_M={\int}_0^H{\left(\frac{\partial M\left(T,H\right)}{\partial T}\right)}_H\  dH $$

where *M* is the magnetization, *H* is the magnetic field, and *T* is the temperature. The relative cooling power (RCP) proposed by Gschneidner et al. [215] is also an important parameter for selecting potential substances for magnetic refrigeration, which is described as the refrigeration capacity of magnetic refrigerant for magnetic refrigeration. It is evaluated using the relation

2 $$ \mathrm{RCP}=\left|{\left(\Delta  {S}_M\right)}^{max}\right|\times \delta {T}_{FWHM} $$

where δT is the full width at half maximum of a -ΔS(T) curve.

**Fig. 22:**
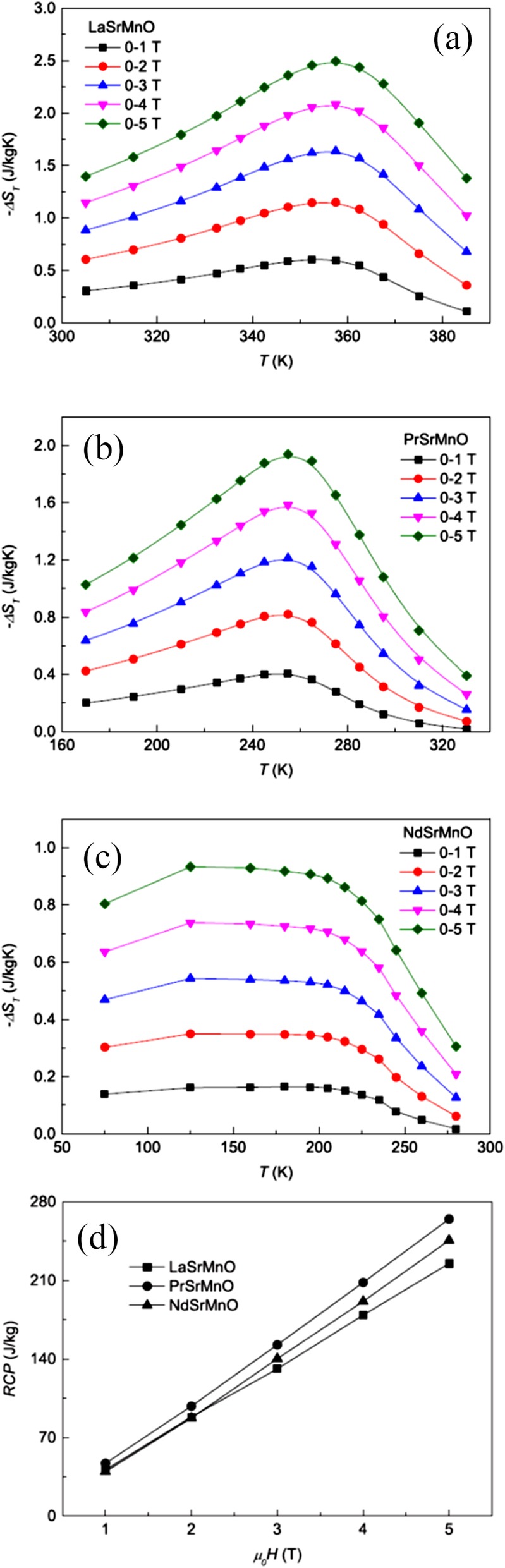
Isothermal entropy changes as a function of temperature with field changes of 1, 2, 3, 4, and 5 T, **a** for LaSrMnO_3_, **b** for PrSrMnO_3_, and **c** for NdSrMnO_3_, respectively. **d** Relative cooling power (RCP) as a function of magnetic field for the Ln_0.67_Sr_0.33_MnO_3_ (Ln = La, Pr, and Nd) nanocrystalline samples. Reproduced with permission of [38]

Wang et al. [38] investigated the magnetocaloric effect in the Ln_0.67_Sr_0.33_MnO_3_ (Ln = La, Pr and Nd) nanoparticles prepared by using the sol-gel method. Figure 22a–c shows the temperature dependence of −∆S(T) under different changes of applied field from 1 to 5 T for LaSrMnO_3_, PrSrMnO_3_, and NdSrMnO_3_ nanoparticles, respectively. Under a field changing from 0 to 5 T, the maximum values of isothermal entropy change are found to be 2.49, 1.94, and 0.93 J/kg K for the samples with Ln = La, Pr, and Nd, respectively, and the corresponding values of RCP reach 225, 265, and 246 J/kg. The RCP as a function of magnetic field is presented in Fig. 22d. It is seen that the RCP increases in almost a linear way as the field increases. These results suggest that those nanoparticles could be useful for magnetic refrigeration in a broad temperature range.

#### Transport Properties

**Fig. 23:**
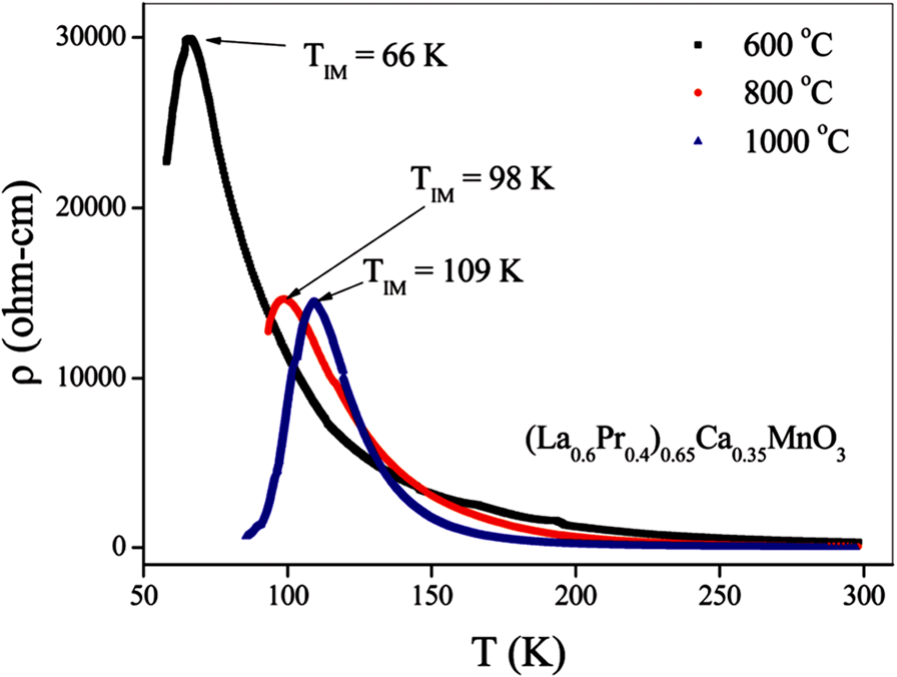
Variation of resistivity with the temperature for the (La0.6Pr0.4)0.65Ca0.35MnO3 nanoparticles sintered at 600 °C, 800 °C, and 1000 °C, respectively. Reproduced with permission of [154]

Transport property measurements of the manganite materials were carried out using standard four-terminal method on a Quantum Design PPMS system. At a constant applied field, resistance was measured as a function of temperature. Kumar et al. [154] synthesized the (La0.6Pr0.4)0.65Ca0.35MnO3 nanoparticles via a sol-gel route at different sintering temperatures, and measured their electrical transport properties. The electrical resistivities (*ρ*) as a function of temperature for the (La_0.6_Pr_0.4_)_0.65_Ca_0.35_MnO_3_ nanoparticles sintered at 600 °C, 800 °C, and 1000 °C was shown in Fig. 23. It was observed that the (La_0.6_Pr_0.4_)_0.65_Ca_0.35_MnO_3_ system shows insulator type behavior at higher temperatures due to the development of charge-ordered states in the nanocrystalline system, and starts to behave as a metal at lower temperatures because double exchange interaction plays a dominant role in the transport behavior of the system. The insulator-metal transition temperature (*T*_IM_) and resistivity (*ρ*) of nanoparticles are dependent upon the sintering temperature of the system (or the particle sizes). With increasing the sintering temperature, the particle (grain) size is increased; therefore, the effect of grain boundary is reduced and consequently the charge carriers in the nanocrystalline system face less scattering from the grain boundaries. This factor also improves the double exchange interaction mechanism and the system starts to show M–I transition at higher temperatures, and the resistivity of the system is also decreased significantly.

**Fig. 24:**
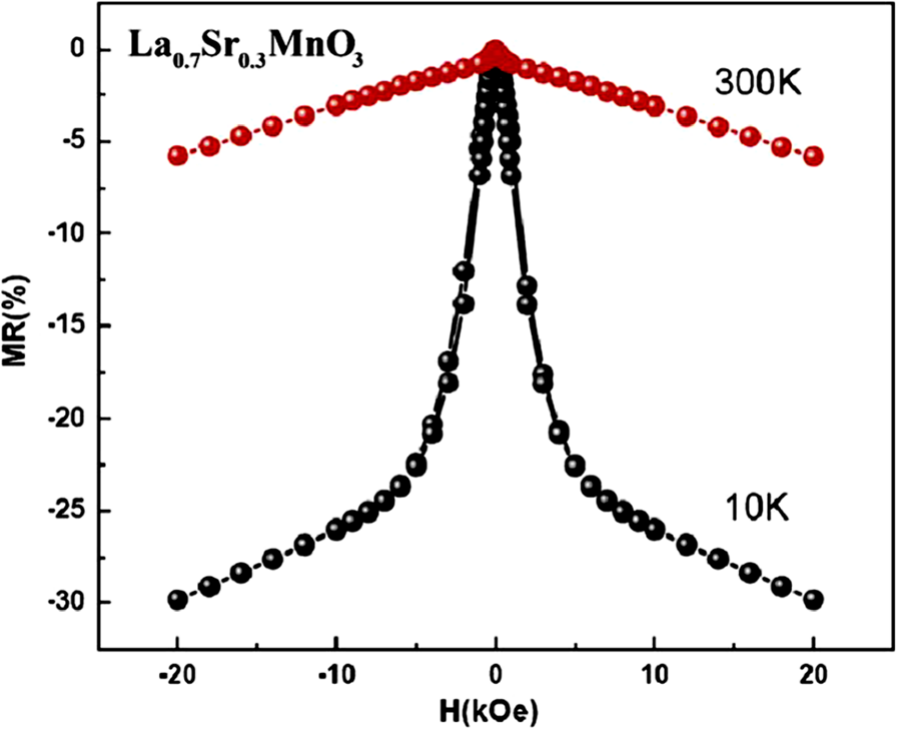
Magnetic field dependence of the MR curves from − 20 to 20 kOe at 10 K and 300 K for the La_0.7_Sr_0.3_MnO_3_ nanoparticles. Reproduced with permission of [44]

Zi et al. [44] prepared La_0.7_Sr_0.3_MnO_3_ nanoparticles by a simple chemical co-precipitation route. To study the magnetoresistance (MR) effect, the magnetic field dependence of MR ratio at 10 K and 300 K by sweeping the applied magnetic field from − 20 to 20 kOe, was shown in Fig. 24. MR is defined as

3 $$ \mathrm{MR}=\frac{\rho_H-{\rho}_0}{\rho_0}\times 100\% $$

where *ρ*_*H*_ and *ρ*_0_ refer to the resistivity under the applied and zero field, respectively. It can be seen that MR drops abruptly with the increasing field in low-field region, which is called as low-field magnetoresistance (LFMR). LFMR values at 10 and 300 K are 22.3% and 2.9% at 5 kOe, respectively. Because of the small coercive field, the alignment of the magnetization in each LSMO grain to the applied magnetic field occurs in the low-field region. At a comparatively high-field region above 5 kOe, MR decreases linearly with the applied field, but with a much reduced slope. High-field MR (HFMR) ratios at 10 and 300 K are 29.2% and 6.5% at 20 kOe, respectively. HFMR can be attributed to the non-collinear spins at LSMO boundaries.

**Fig. 25:**
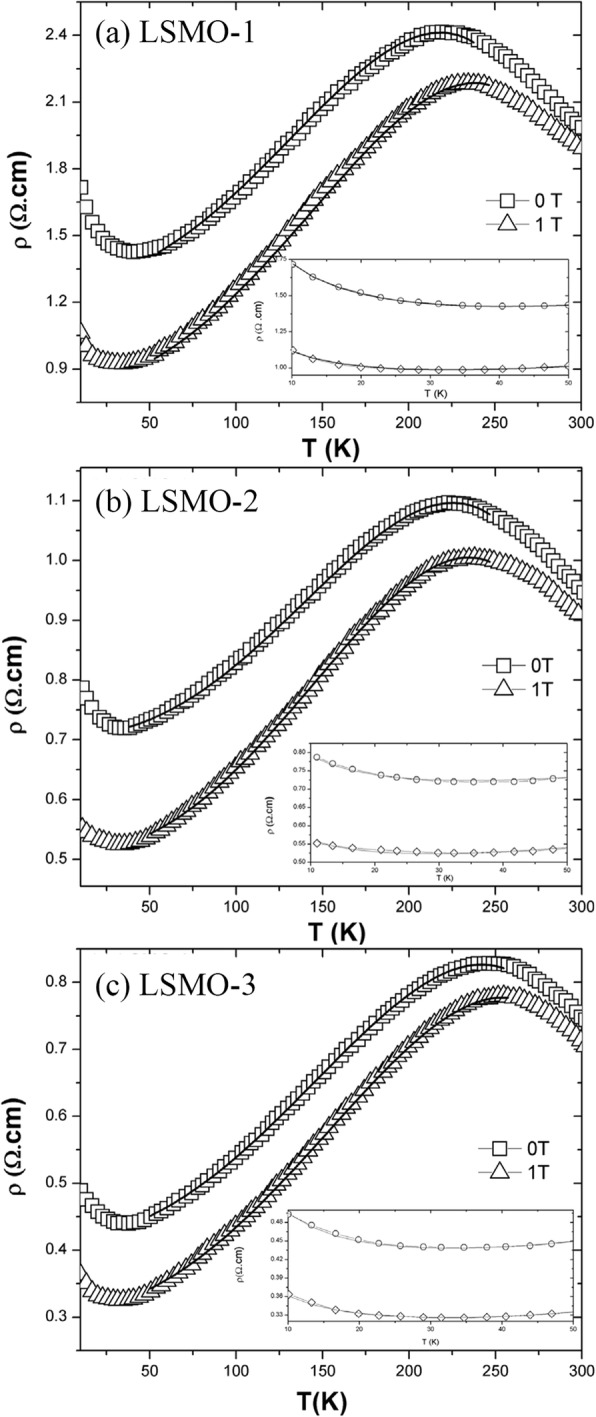
Temperature dependence resistivity of the La_0.7_Sr_0.3_MnO_3_ (LSMO) nanoparticles with zero field and 1 T fitted by using *ρ*(*T*) = *ρ*_0_ + *ρ*_2_*T*^2^ + *ρ*_4.5_*T*^4.5^. **a** LSMO-1 particles (average size of 20 nm), **b** LSMO-2 particles (average size of 23 nm), and **c** LSMO-3 particles (average size of 26 nm). Inset shows the fitting of the resistivity data in the low temperature region by using ${\rho}_L={\rho}_0-{\rho}_s\ln T+{\rho}_e{T}^{\frac{1}{2}}+{\rho}_p{T}^5$. Reproduced with permission of [216]

**Fig. 26:**
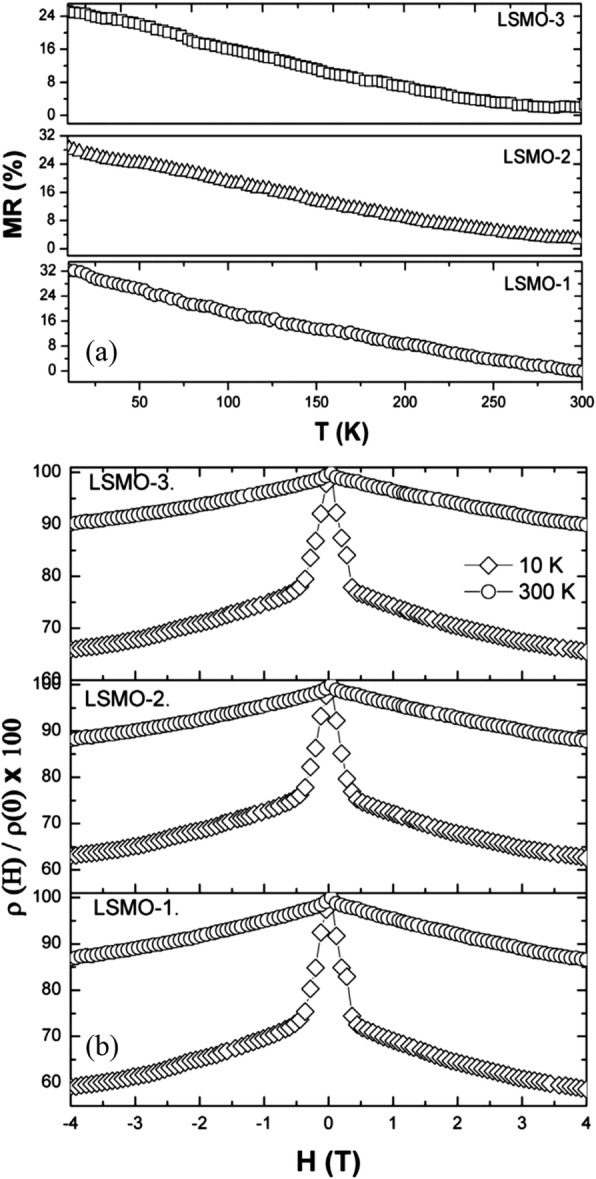
**a** Temperature dependence of the magnetoresistance (MR %) at 1 T. **b** Normalized resistivity as a function of the applied field at 10 K and 300 K of the La_0.7_Sr_0.3_MnO_3_ (LSMO) nanoparticles. LSMO-1, -2, and -3 particles with average particle sizes of 20 nm, 23 nm, and 26 nm, respectively. Reproduced with permission of [216]

To investigate the particle size effect on the transport properties of La_0.7_Sr_0.3_MnO_3_ (LSMO) nanoparticles, Navin and Kurchania [216] synthesized the LSMO nanoparticles with particle sizes of 20 nm (LSMO-1), 23 nm (LSMO-2), and 26 nm (LSMO-3), respectively. Figure 25 shows their temperature dependence of resistivity measured under (H = 1 T) and without magnetic field in the temperature range of 10–300 K. It was found that the resistivity values of the LSMO-1 nanoparticles (Fig. 25a) were higher than that of the LSMO-2 (Fig. 25b) and LSMO-3 (Fig. 25c) nanoparticles. That is ascribed to the smaller particle size in the LSMO-1 sample. As the particle sizes become smaller, more grain boundaries in the samples acts as scattering centers to the charge carriers, resulting in the larger resistivity. Besides, it was found that the value of resistivity of all samples decrease under an external magnetic field of 1 T. The applied magnetic field gives rise to the increasing of spin ordering and the decreasing of the localization of the charge, which result in the reduction of resistivity. The LFMR property is related to the spin dependent scattering or spin dependent tunneling of the conduction electrons near the interfaces and grain boundaries. Figure 26a shows the temperature dependence of the MR of the samples LSMO-1, LSMO-2, and LSMO-3 at an applied magnetic field of 1 T. It was found that their MR values increase monotonically with decreasing the temperature. The LFMR at 1 T and 10 K for the samples LSMO-1, LSMO-2, and LSMO-3 was obtained as 32.3%, 28.4%, and 25.1% respectively. Obviously, the LFMR enhanced with decreasing the particle sizes. Figure 26b shows the magnetic field dependence of the normalized resistivity (ρ_H_/ρ_o_) with applied magnetic field at temperatures 10 K and 300 K, where ρ_0_ and ρ_H_ are the resistivity without and with magnetic field respectively. A sharp drop in the value of resistivity in low magnetic field region was also observed and the resistivity does not saturate up to a magnetic field of 4 T.

**Fig. 27:**
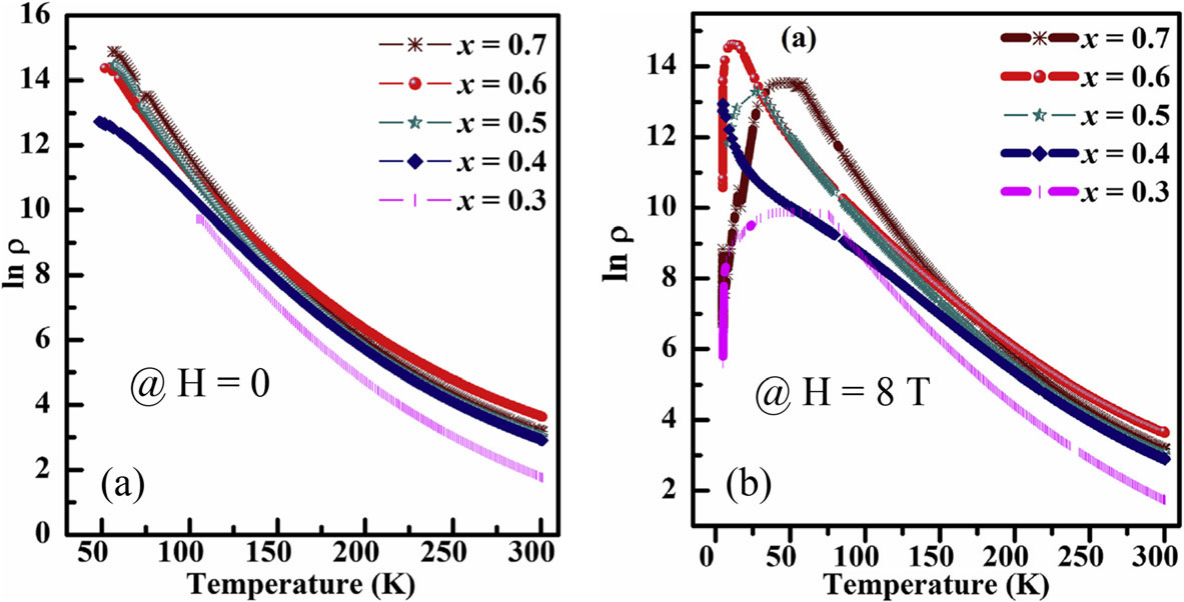
Temperature dependent resistivity for the Nd_1-x_Sr_x_MnO_3-δ_ nanoparticles under different external magnetic fields. **a** H = 0 and **b** H = 8 T. Reproduced with permission of [217]

The effects of doping levels on the electrical transport properties of perovskite manganite nanoparticles were also investigated. Thombare et al. [217] reported the electrical properties of Nd_1-x_Sr_x_MnO_3-δ_ (NSMO, 0.3 ≤ × ≤ 7) nanoparticles synthesized by glycine assisted auto combustion method. Figure 27a shows the resistivity for NSMO in the temperature range 5–300 K at zero magnetic field. It is found that all plots show high resistivity. The resistivity values slightly increases with the Sr concentration up to 100 K, whereas below that the rise in resistivity is steeper. The M–I transition is not observed in the present NSMO nanoparticles without applied magnetic field. However, under applied magnetic field (H) of 8 T, the M–I transition temperature (T_P_) was clearly observed around 12–48 K, as shown in Fig. 27b. These transition temperatures are lower than that in bulk counterpart. That may be due to the formation of small ferromagnetic clusters which are suffice for magnetic contribution but forbids conduction [218].

#### Optical Properties

**Fig. 28:**
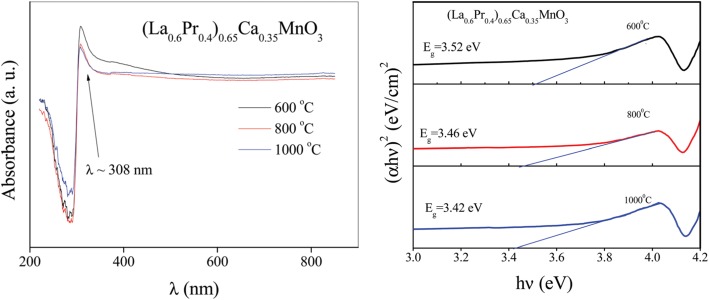
**a** Ultraviolet-visible spectra of the (La_0.6_Pr_0.4_)_0.65_Ca_0.35_MnO_3_ nanoparticles sintered at 600 °C, 800 °C, and 1000 °C. **b** Variation of (*αhν*)^2^ versus photon energy *hν* plot for the (La_0.6_Pr_0.4_)_0.65_Ca_0.35_MnO_3_ nanoparticles sintered at 600 °C, 800 °C, and 1000 °C. Reproduced with permission of [154]

The optical study of perovskite manganites has interestingly shown that they are controlled by the electronic structure of perovskites. Kumar et al. [154] synthesized (La_0.6_Pr_0.4_)_0.65_Ca_0.35_MnO_3_ nanoparticles by sol-gel method and post-annealed at 600 °C, 800 °C, and 1000 °C. To investigate the optical absorbance and evaluate the optical band gap of (La_0.6_Pr_0.4_)_0.65_Ca_0.35_MnO_3_, ultraviolet-visible (UV-Vis) spectroscopy measurements are carried out and the obtained UV-Vis spectra are shown in Fig. 28a. Obviously, there is a sharp absorption edge around 308 nm in ultraviolet region. The optical absorption edges can be analyzed as follows [219]:

4 $$ \alpha hv\propto {\left( hv-{E}_g\right)}^{\mathrm{n}} $$

where *E*_g_ is the band gap energy, *hν* is the photon energy, and *α* is the absorption coefficient, depending upon the optical absorbance (A) and thickness (d). *n* can be equal to 1/2 (for direct transition process) or 2 (for indirect transition process). The variations of (*αhν*)^2^ versus photon energy (*hν*) for the (La_0.6_Pr_0.4_)_0.65_Ca_0.35_MnO_3_ nanoparticles post-annealed at 600 °C, 800 °C, and 1000 °C are plotted in Fig. 28b. It is observed that (αhν)^2^ varies linearly for a very wide range of photon energy (*hν*), indicating a direct type of transitions in these systems. The intercepts of these plots on the energy axis give the energy band gaps of the systems, which were determined to be 3.52, 3.46, and 3.42 eV, respectively, for the (La_0.6_Pr_0.4_)_0.65_Ca_0.35_MnO_3_ nanoparticles post-annealed at 600 °C, 800 °C, and 1000 °C. These direct band gaps fall into the range of wide band gap semiconductors. The decrease of the band gap (red-shift) with increasing post-annealing temperature can be attributed to the increased particle sizes. Negi et al. [220] also investigated the optical properties of GdMnO_3_ nanoparticles synthesized by the modified sol-gel route. The room temperature optical absorption spectrum of the GdMnO_3_ nanoparticles measured in the range of 200–600 nm clearly shows that the absorbance is less in the range of 380–600 nm. The low absorbance in the entire visible region is an essential condition for nonlinear optical applications [221]. An extrapolation of the linear region of a plot of *(αhν)*^2^ on the *y*-axis versus photon energy (*hν*) on the *x*-axis gives the optical band gap ∼ 2.9 eV.

### 1D Rare Earth-Doped Perovskite Manganite Oxide Nanostructures

#### Magnetic Properties

Chandra et al. [222] synthesized La_0.67_Ca_0.33_MnO_3_ crystalline nanowires with the average diameter of 70 nm by using porous templates of anodized alumina combined with CSD technique. Their temperature dependence of magnetization (M) measured at ZFC and FC modes and under a magnetic field of 100 Oe demonstrates that these nanowires undergo a PM to FM transition at *T*_C_ = 245 K, which is defined by the minimum in *dM*/*dT*. Similar feature was also reported for the La_0.67_Ca_0.33_MnO_3_ nanotubes [87]. Datta et al. [157] also synthesized La_1-x_A_x_MnO_3_ (where A = Ca, Sr; *x* = 0.3 and 0.5) nanowires by hydrothermal method. All the nanowires undergo FM–PM phase transitions with increasing the temperature. Their *T*_C_ values are dependent upon the crystal structure as well as the Mn valence and oxygen stoichiometry.

**Fig. 29:**
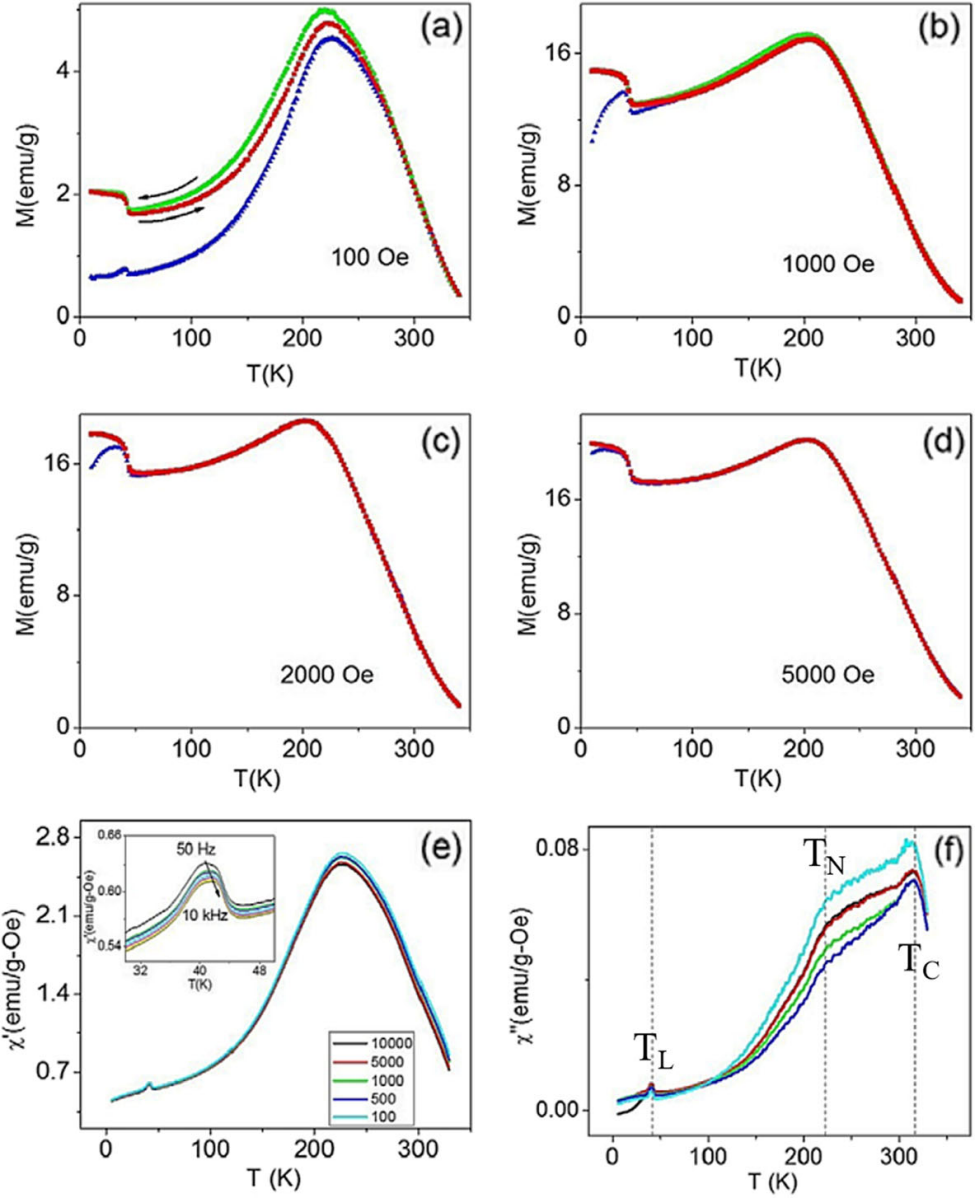
**a**, **b** Zero-field-cooled (ZFC) (blue), field-cooled-cooling (FCC) (green), and field-cooled-warming (FCW) (red) magnetization curves of the La_0.5_Sr_0.5_MnO_3_ nanowires measured under applied fields of **a** 100 Oe and **b** 1000 Oe. **c**, **d** ZFC and FCW magnetization curves obtained under applied magnetic field of **c** 2000 Oe and **d** 5000 Oe. **e** Real and **f** imaginary parts of linear ac susceptibility *versus* temperature plots for different frequencies. The inset of (**e**) shows a magnified view of *χ’(T*) for more frequencies. Reproduced with permission of [224]

Wang and Fan [223] reported on the magnetic properties of electron-doped Ca_0.82_La_0.18_MnO_3_ nanowires and nanoparticles, and compared them with their bulk counterpart. It is found that the Ca_0.82_La_0.18_MnO_3_ bulk exhibits a strong charge ordering (CO) peak at *T*_CO_ = 132 K followed by an AFM ground state, whereas the CO peak becomes weak in the nanowires (*T*_CO_ = 124 K), and disappeared in the nanoparticles which exhibits a ferromagnetism with *T*_C_ = 165 K. Chandra et al. [224] also reported on the magnetic properties of the single-crystalline La_0.5_Sr_0.5_MnO_3_ nanowires with diameter of 20–50 nm and length of 1–10 μm synthesized by the hydrothermal technique. Figure 29a–d shows their temperature dependence of dc magnetization *M*(T) measured under different applied magnetic fields. As the temperature is lowered from 340 K, the nanowires exhibit a PM to FM transition at *T*_C_∼315 K followed by a peak at *T*_N_∼210 K, which is associated with the onset of the FM–AFM transition. As the applied magnetic field is increased, the irreversible temperature shifts to lower temperatures as shown in Fig. 29b–d. Figure 29e, f demonstrates the temperature dependences of the real (*χ*′) and imaginary (*χ*′′) parts of ac susceptibility in the temperature range of 10–340 K, respectively. The *χ*′ (*T*) curves show a maximum at *T*_N_ with no frequency dependence and a kink at *T*_L_. In addition, the *χ*′′ (*T*) curves, which reveal insight into magnetic loss behavior, showing a peak at *T*_C_, a broad shoulder at *T*_N_, and a kink at *T*_L_. The *χ*′ (*T*) peak shifts to a higher temperature as the frequency is increased, which is consistent with the results reported previously [225, 226].

#### Magnetocaloric properties

**Fig. 30:**
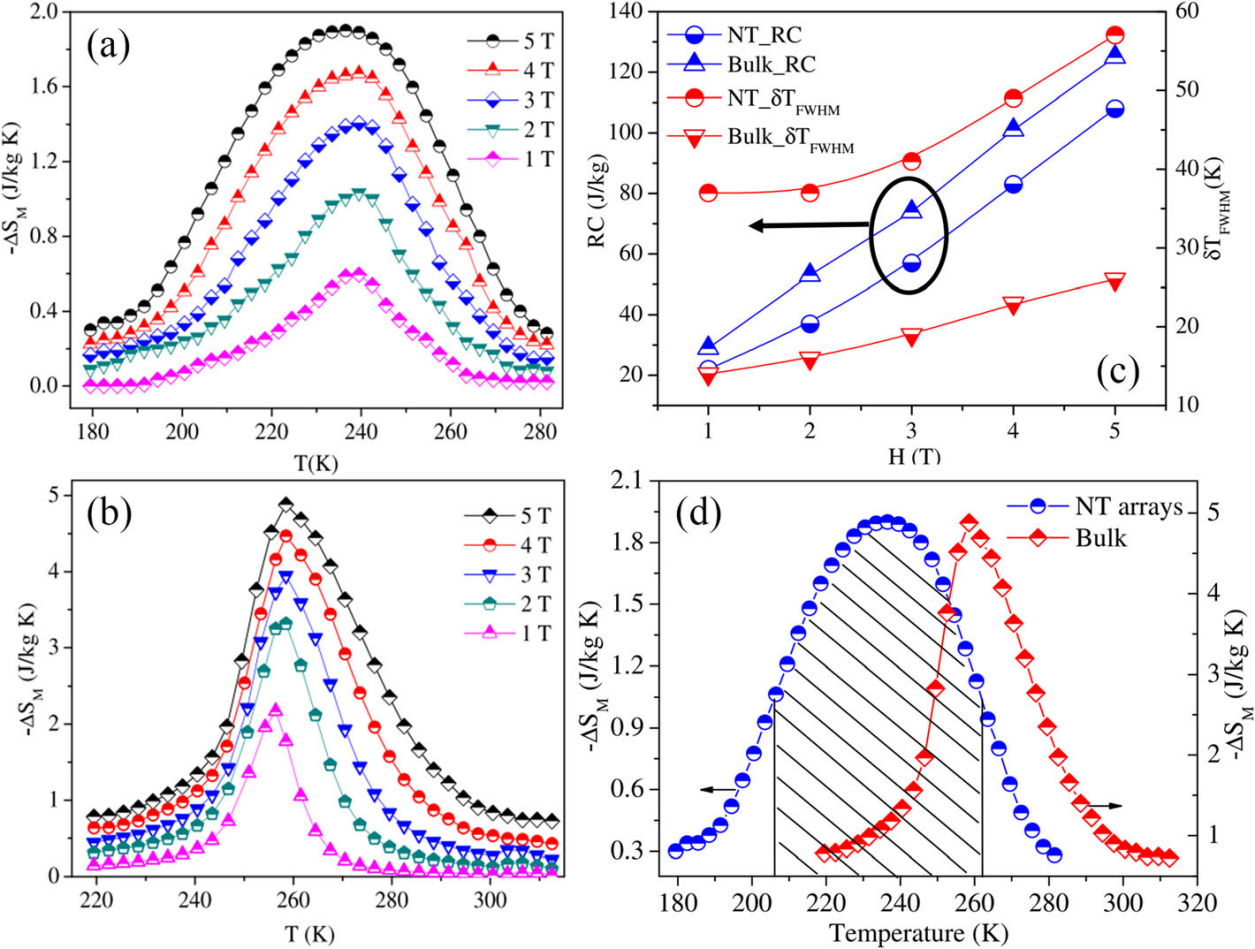
Temperature dependence of −*∆S*_*M*_ curves for **a** La_0.7_Ca_0.3_MnO_3_(LCMO) nanotube (NT) arrays and **b** LCMO bulk at different fields. **c** RC and *δ*T_FWHM_ with respect to the field of the LCMO NT arrays and LCMO bulk sample. **d** Temperature dependence of −*∆S*_*M*_ curves of the LCMO NT arrays and bulk at 5 T. Reproduced with permission of [227]

Kumaresavanji et al. [227] reported on the MCE in La_0.7_Ca_0.3_MnO_3_ nanotube arrays, which were synthesized by template-assisted sol-gel method in temperatures ranging from 179 to 293 K and under magnetic fields up to 5 T. Their temperature dependence of −*∆S*_*M*_ at different fields for nanotube arrays and bulk is plotted in Fig. 30a, b. When compared with the bulk counterpart (4.8 J/kg K), the magnitude of the *∆S*_*M*_ (1.9 J/kg K) is smaller for nanotube arrays. In addition, the temperature dependence of −*∆S*_*M*_ curves for bulk sample show a narrow peak at 258 K which become broader and shift to lower temperature for nanotube arrays. The refrigerant capacitance (RC), is also an important parameter for selecting potential substances for magnetic refrigeration, which is described as the amount of heat transferred between the hot and cold sinks in one ideal refrigeration cycle. It is evaluated using the relation

5 $$ RC(H)={\int}_{T_1}^{T_2}\Delta  {S}_M\left(T,H\right) dT $$

where *T*_1_ and *T*_2_ are the temperatures of cold and hot reservoirs, respectively, which correspond to the full width at half maximum (δT_FWHM_) of the *∆S*_*M*_ curves. The calculated δT_FWHM_ and RC values for nanotube arrays and bulk samples are depicted in Fig. 30c. The RC values vary linearly with H in both cases. Moreover, the RC value is reasonably large for bulk sample compared to nanotube arrays. However, the δT_FWHM_ values of nanotube arrays are nearly 54% larger than the observed one for bulk sample. The temperature dependence of *∆S*_*M*_ curves of nanotube arrays and bulk at a field of 5 T are comparatively shown in Fig. 30d. The shadow part represents the δT_FWHM_ of nanotube arrays which is nearly three times larger than their bulk counterpart. From this figure, one can understand how the nanotube arrays provide an expanded working temperature range compared to the bulk one. Even though the nanotube arrays present a broader *∆S*_*M*_ curve, the *∆S*_*M*_ value is lower compared to the bulk sample. However, the higher surface to volume ratio together with the hollow structure and broader peaks of *∆S*_*M*_ indicate that the manganite nanotubes could be a suitable material for magnetic refrigeration in nano-electromechanical systems. The magnetocaloric properties of La_0.6_Ca_0.4_MnO_3_ nanotubes with diameter of 280 nm and wall thickness of 10 nm were also reported by Andrade et al. [228]. It is found that the decrease of *∆S* is commonly accompanied by a broadening in the *∆S* curve. The RCP of nanoparticles is decreased with decreasing the particle size, but they still possess a larger cooling power than the nanotubes of the same compound, due to the broadening of the magnetic transition observed in these samples. In this way, it is important to notice that the reduced maximum value of *∆S* observed for nanosystems is often accompanied by a broad magnetic entropy change.

#### Transport Properties

**Fig. 31:**
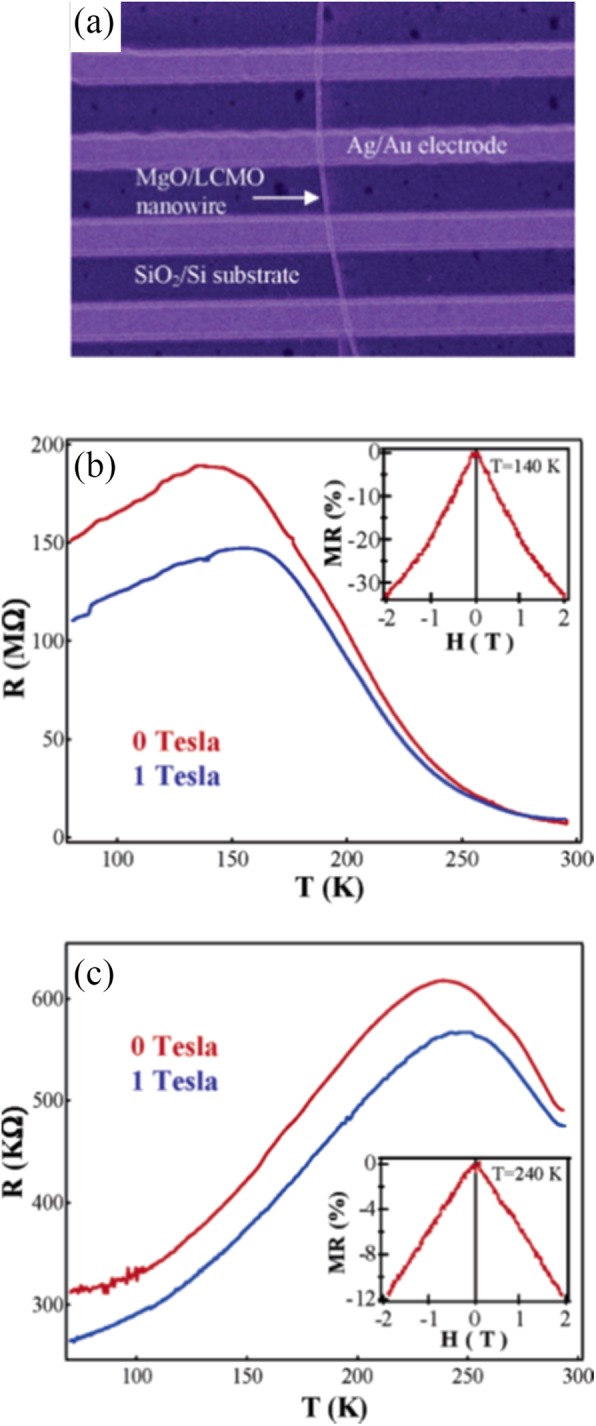
**a** Top view SEM image of a core-shell nanowire device showing four Ag/Au contact electrodes with even spacing. **b**, **c** Four-probe resistance (*R*) vs. temperature (*T*) curves measured under two different magnetic fields *B* = 0 T (red) and *B* = 1 T (blue), with **b** MgO/LaCaMnO_3_(LCMO) nanowire and **c** MgO/LaSrMnO_3_ (LSMO) nanowire. Insets of panels **b**, **c** magnetoresistance (MR) recorded at temperature *T* = 140 K for panel **b** and *T* = 240 K for panel **c**, respectively. Reproduced with permission of [229]

Lei et al. [229] successfully synthesized single-crystalline MgO/La_0.67_Ca_0.33_MnO_3_ core-shell nanowires (MgO core is ∼ 20 nm in diameter and the La_0.67_Ca_0.33_MnO_3_ shell layer is ∼ 10 nm in thickness) by depositing epitaxial La_0.67_Ca_0.33_MnO_3_ sheaths onto MgO nanowire templates through the PLD technique. Transport investigations were carried out by measuring the four-probe resistance of individual core-shell nanowires, as shown in Fig. 31. The SEM image of a typical device with a 5-μm-long nanowire and four uniformly distributed electrodes is shown in Fig. 31a. The four-probe resistance of an MgO/La_0.67_Ca_0.33_MnO_3_ nanowire was recorded as a function of temperature under two different magnetic fields (0 and 1 T), as shown in Fig. 31b. This M–I transition occurred at ∼ 140 K under zero magnetic field, and the transition temperature shifted to ∼ 160 K when a magnetic field of 1 T was applied normal to the device substrate. This M–I transition and transition temperature shifting effect induced by magnetic fields strongly suggest a correlation between the ferromagnetism and the metallicity, which is ascribed to the double-exchange mechanism. This M–I transition associated with FM to PM transition also happened in the MgO/La_0.67_Sr_0.33_MnO_3_ core-shell nanowires with *T*_MI_∼240 K at *H* = 0 and the *T*_MI_ shifted to ∼ 250 K under a perpendicular magnetic field of 1 T (Fig. 31c). In addition, MR measurements were also performed with both MgO/La_0.67_Ca_0.33_MnO_3_ (inset of Fig. 31b) and MgO/La_0.67_Sr_0.33_MnO_3_ (inset of Fig. 31c) core-shell nanowires at their transition temperature by sweeping the perpendicular magnetic field between ± 2.0 T. By defining the MR ratio as [R(H) - R(0)]/R(0) × 100%, a value of MR = 34% was achieved at *T* = 140 K and *H* = 2.0 T for La_0.67_Ca_0.3d3_MnO_3_ (inset of Fig. 31b), and 12% was achieved at *T* = 240 K and *H* = 2.0 T for La_0.67_Sr_0.33_MnO_3_ (inset of Fig. 31c).

#### Optical Properties

**Fig. 32:**
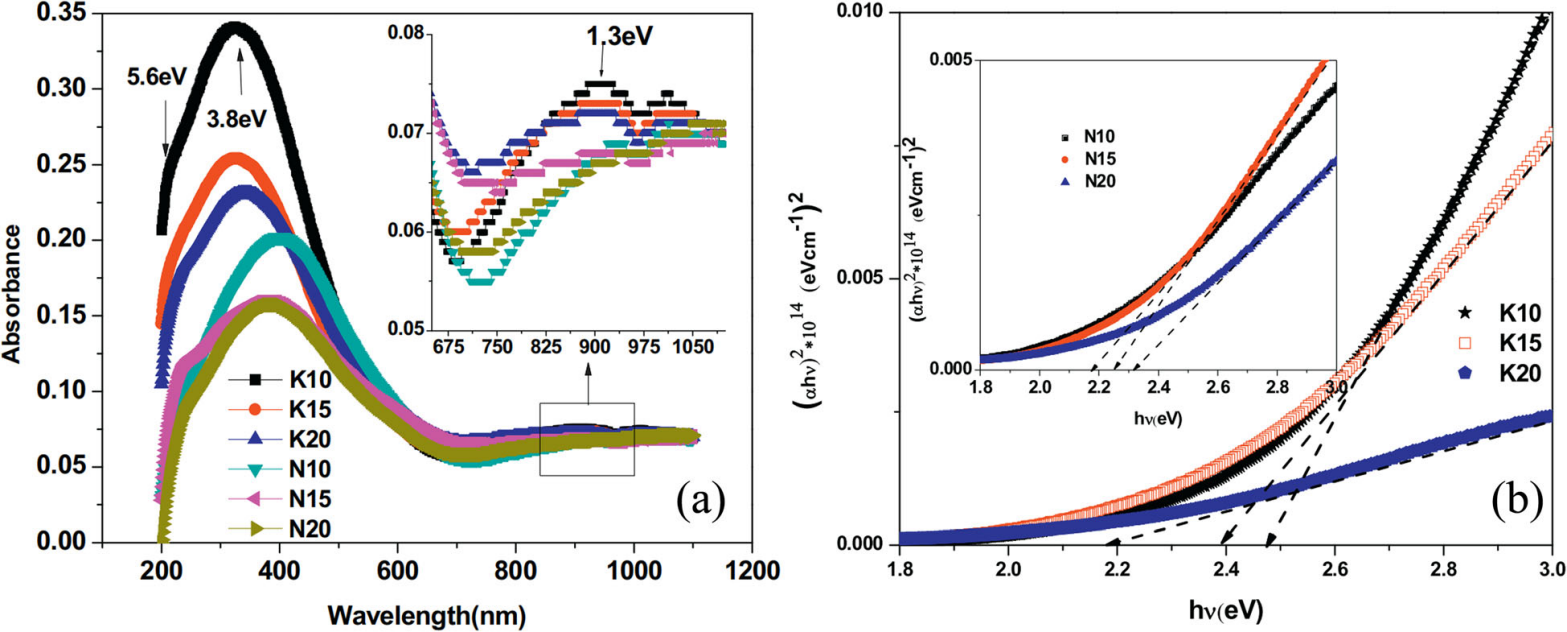
**a** UV–Vis absorption response of the La_0.7_Ca_0.3_MnO_3_ (LCMO) nanorods synthesized by hydrothermal method under different alkalinity conditions. **b** The variation of (α*hv*)^2^ absorption versus *hv* (photon energy) for the different samples (N10, N15, N20, K10, K15, and K20). N (or K) means the NaOH (or KOH) mineralizer, 10 (or 15, 20) for the NaOH (or KOH) concentration. Reproduced with permission of [80]

Arabi et al. [80] synthesized the La_0.7_Ca_0.3_MnO_3_ nanorods by hydrothermal method under different conditions (e.g., different mineralization agents KOH and NaOH; various alkalinity conditions (10, 15, and 20 M)). The UV–Vis absorption spectra of all the La_0.7_Ca_0.3_MnO_3_ nanorods are shown in Fig. 32a, where three obvious peaks are observed due to optical response in these La_0.7_Ca_0.3_MnO_3_ nanorods. The first peak was observed around 220 nm (5.6 eV) for all the samples. Strong absorption peak appeared at wavelengths about 325–380 nm (3.8–3.3 eV) and the third peak appeared around 950 nm (1.3 eV) in all the samples, as shown in inset of Fig. 32a. It is found that a decrease and broadening of the absorption peaks for the N-series samples is related to size reduction. Figure 32b shows the curves of (*αhv*)^2^ versus *hv*, and the intercepts of these plots on the *hν* axis provide the optical band gaps. It can be obtained that the main band gaps are estimated to be 2.48, 2.39, and 2.19 eV for the samples K10, K15, and K20 as well as 2.18, 2.25, and 2.32 eV for the samples N10, N15, and N20, respectively. Recently, Arabi et al. [230] also investigated the optical properties of La_0*.*68_Ca_0*.*32_MnO_3_ nanowires prepared by hydrothermal method. Their optical band gap is estimated to be about 2.13 eV.

### 2D Rare Earth-Doped Perovskite Manganite Oxide Nanostructures

#### Magnetic Properties

**Fig. 33:**
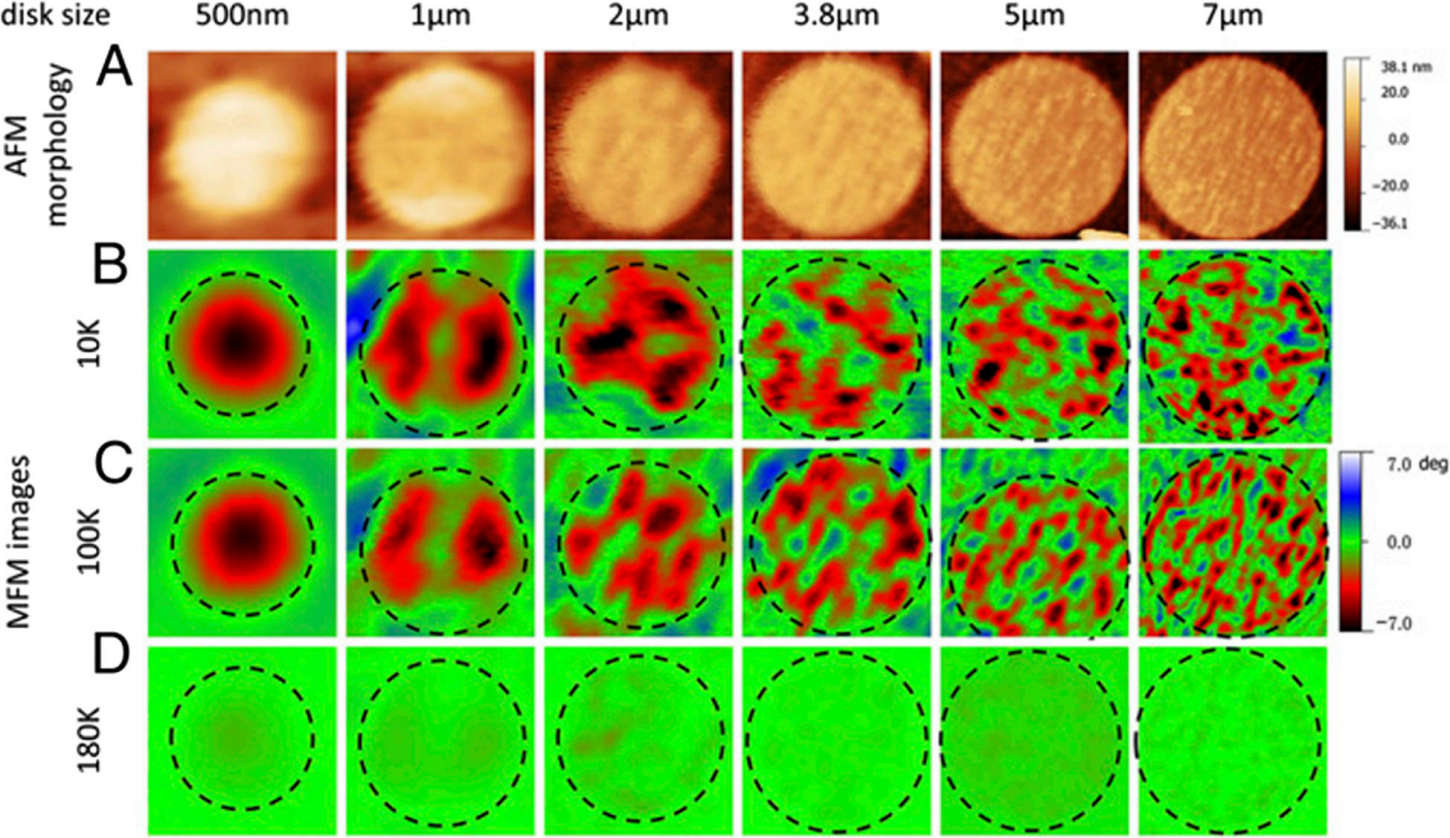
**a** AFM images of the La_0.325_Pr_0.3_Ca_0.375_MnO_3_ (LPCMO) disks with diameter sizes of 500 nm, 1 μm, 2 μm, 3.8 μm, 5 μm, and 7 μm in diameter. **b**–**d** MFM images of the LPCMO disks under external magnetic field of 1 T (external magnetic field direction is pointing perpendicularly to the sample surface plane) taken at 10 K (**b**), 100 K (**c**), and 180 K (**d**). The negative value in MFM image indicates attractive force and positive value indicates repulsive force. Reproduced with permission of [106]

Shao et al. [106] fabricated La_0.325_Pr_0.3_Ca_0.375_MnO_3_ single-crystalline disks with diameters from 500 nm to 20 μm to study the spatial confinement effect on EPS. By using electron beam lithography with a negative tone resist, the La_0.325_Pr_0.3_Ca_0.375_MnO_3_ disks were derived from the epitaxial La_0.325_Pr_0.3_Ca_0.375_MnO_3_ films with thickness of 60 nm grown on STO(001) substrates by PLD. Magnetic properties of these La_0.325_Pr_0.3_Ca_0.375_MnO_3_ disk arrays are measured by SQUID and MFM. Figure 33 shows the transition from the EPS state to a single ferromagnetic metallic (FMM) state. Figure 33a shows the AFM images of the morphologies for the La_0.325_Pr_0.3_Ca_0.375_MnO_3_ disks with different diameters. The corresponding MFM images of the La_0.325_Pr_0.3_Ca_0.375_MnO_3_ disks acquired at different temperatures (10 K, 100 K, and 180 K) under a perpendicular magnetic field of 1 T are shown in Fig. 33b–d. In the color scale, the contrast below zero (red or black) represents FMM phase, while the contrast above zero (green or blue) represents non-ferromagnetic phase. Obviously, all the disks show distinct features of the EPS state (i.e., the coexistence of the FMM and charge order insulating (COI) phases), except for the 500 nm disk. The typical length scale of the EPS domains is around a micrometer. It was also found that with decreasing temperature, the portion of FMM phase was increased.

**Fig. 34:**
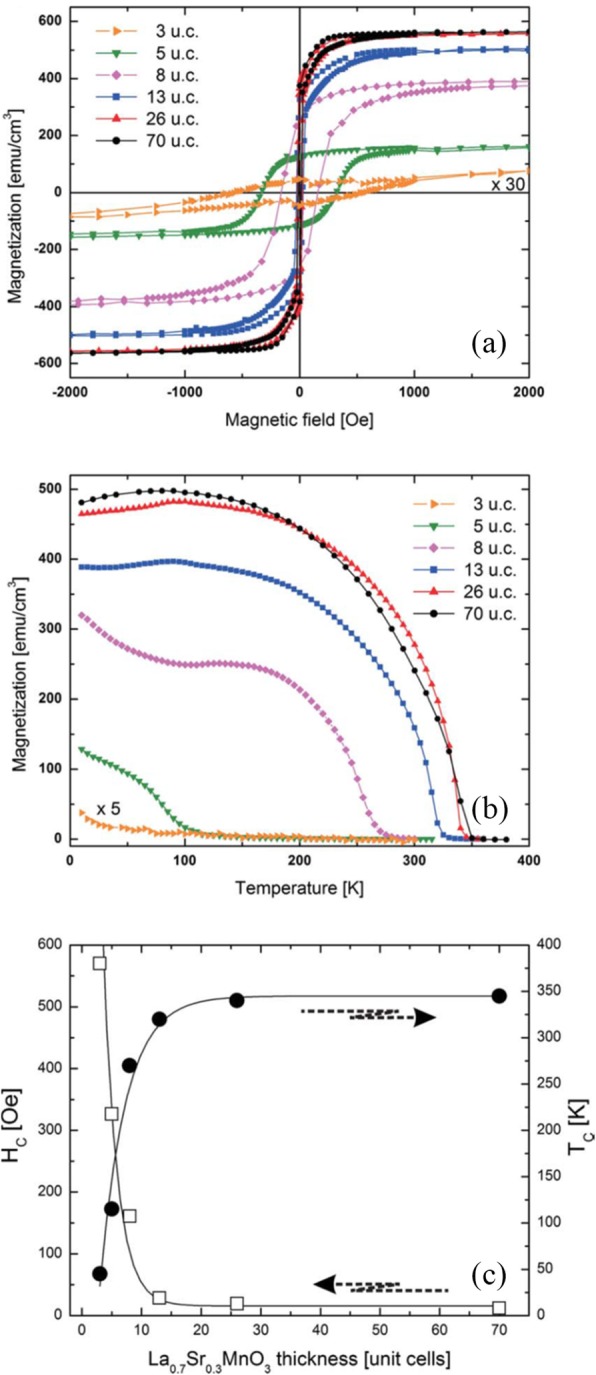
Ferromagnetic properties of ultrathin La_0.7_Sr_0.3_MnO_3_ films on SrTiO_3_ (001). **a** Magnetic hysteresis loops measured at 10 K. The diamagnetic contribution to magnetization not shown has been attributed to the substrate and has been subtracted. **b** Temperature dependence of the magnetization measured at 100 Oe. All samples were field cooled at 1 T from 360 K along the [100] direction before the measurements were performed. **c** Layer thickness dependence on the coercive field *H*_*C*_ and the Curie temperature *T*_*C*_. Reproduced with permission of [231]

Huijben et al. [231] grew ultrathin La_0.7_Sr_0.3_MnO_3_ films with thicknesses from 3 to 70 unit cells on STO substrates by PLD method. Their magnetic properties are shown in Fig. 34. Figure 34a shows the *M*-*H* loops of all samples. It is observed that the saturation magnetization (*M*_S_) is increased with increasing the film thickness up to 13 unit cells (~ 48 Å), whereas the coercive field (*H*_C_) is decreased. The *M*-*T* curves for all the films with different thickness are displayed in Fig. 34b, from which the Curie temperature *T*_C_ is determined. The thicknesses dependent of *H*_C_ and *T*_C_ is shown in Fig. 34c. It is found that *H*_C_ and *T*_C_ are nearly constant for thicknesses down to 13 unit cells. Further reduction in the film thickness results in a dramatic change in the magnetic properties, although the films remain ferromagnetic down to three unit cells (~ 12 Å).

#### Magnetocaloric Properties

**Fig. 35:**
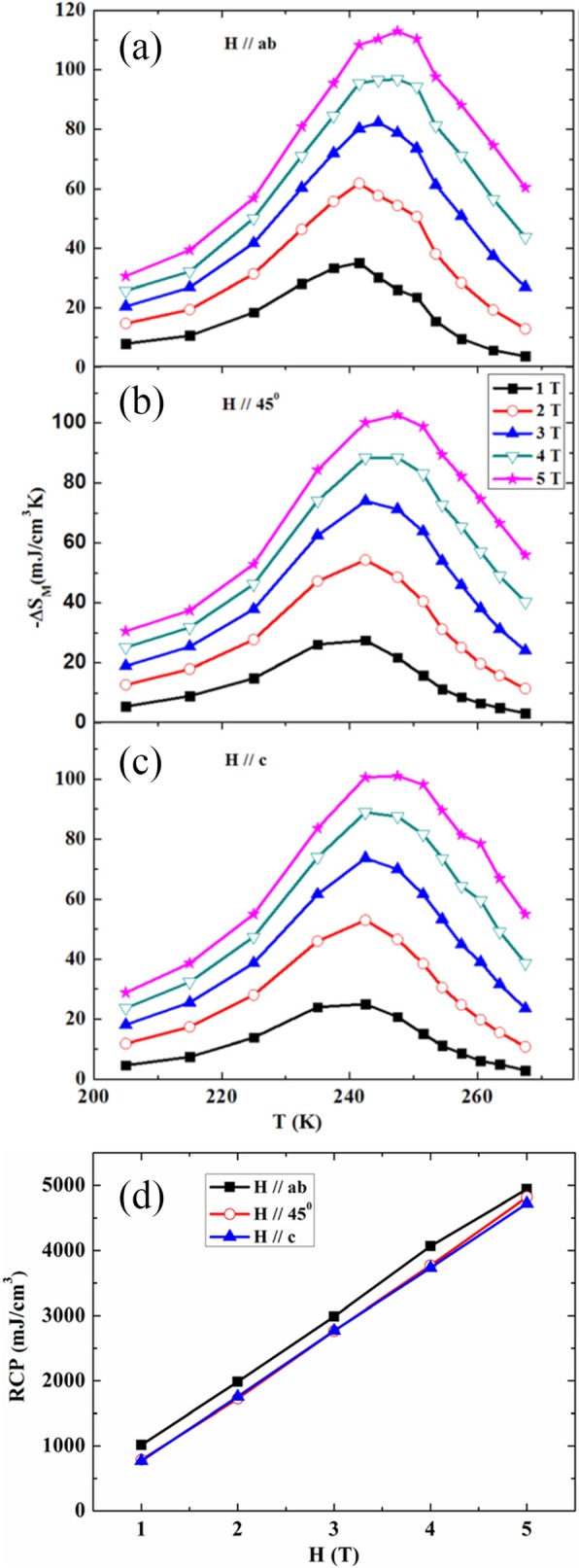
**a–c** Magnetic entropy changes in the La_0.8_Ca_0.2_MnO_3_ thin films as a function of temperature and external magnetic field in different directions. **a** H//*ab*, **b** H//45°, and **c** H//*c*. **d** Relative cooling power (RCP) of the thin film as a function of magnetic field in different field directions. Reproduced with permission of [232]

Debnath et al. [232] reported on the magnetocaloric properties of an epitaxial La_0.8_Ca_0.2_MnO_3_/LaAlO_3_ thin film grown by PLD method. The magnetic entropy changes in the La_0.8_Ca_0.2_MnO_3_ thin film for different magnetic field directions are shown in Fig. 35a–c, respectively. |*∆S*_*M*_| exhibits a peak with its maximum around 247 K near *T*_C_. The maximum values of |*∆S*_*M*_| were estimated to be 35.90, 27.50, and 24.97 mJ cm^−3^ K^−1^ under a field change of 1 T for the different field directions, H//ab, H//45°, and H//c, respectively. The |*∆S*_*M*_| peaks in all directions are significantly broadened over a wider temperature region. The RCP values of the thin film under different field directions are shown in Fig. 35d. Large RCP values (i.e., 1000 mJ/cm^3^ for the ab plane and 780 mJ/cm^3^ for the c-direction) are obtained, which are higher than those observed in other perovskite manganites and rare earth alloys [213, 233, 234]. Such higher entropy change value and higher RCP with no noticeable hysteresis loss will make the epitaxial La_0.8_Ca_0.2_MnO_3_ films more attractive for use as a magnetic refrigeration with large useful temperature ranges.

**Fig. 36:**
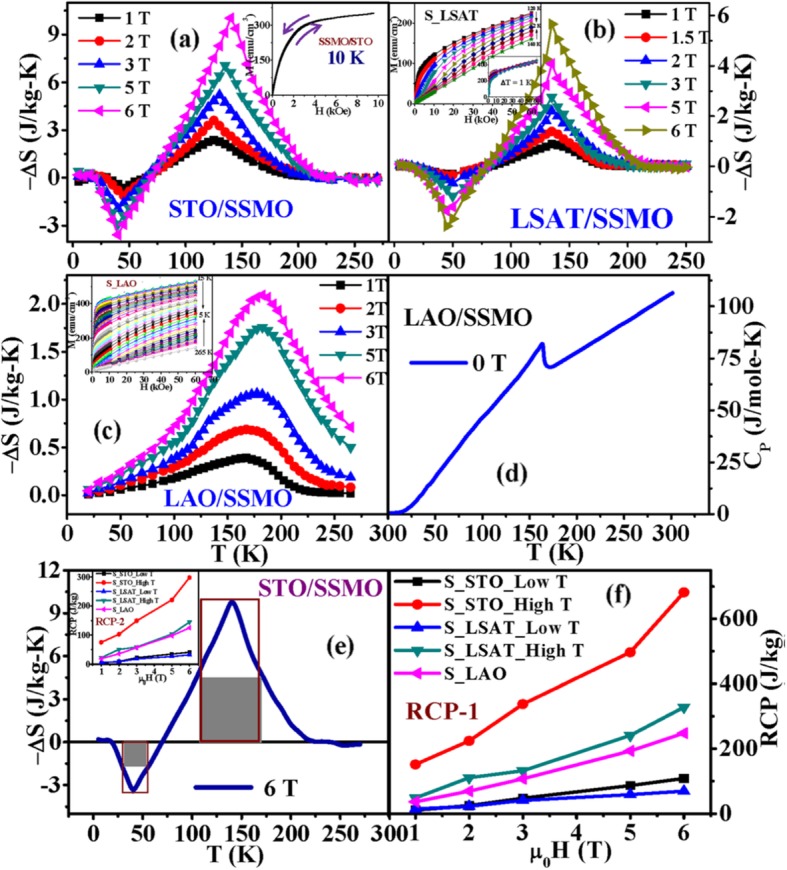
**a–c** Temperature dependent|*∆S*_*M*_| at different magnetic field calculated from M–H data for the SSMO/STO, SSMO/LSAT, and SSMO/LAO films, respectively. Inset of (**b**) and its inset shows the one cycle M–H curves of the SSMO/LSAT film in the temperature interval of 2 K and 1 K, respectively. Inset of (**c**) shows one cycle M–H curves of the SSMO/LAO films in the temperature interval of 5 K. **d** C_P_(T) of the SSMO/LAO film at *μ*_0_H = 0 T. **e** The *∆S*_*M*_ vs. T curve of the SSMO/STO film at *μ*_0_H = 6 T. The bigger rectangular sketch and shaded area corresponds to RCP-1 and RCP-2, respectively. Inset shows the value of RCP-2 as a function of magnetic field for three different films. **f** The value of RCP-1 as a function of magnetic field for three different films. Sm_0.55_Sr_0.45_MnO_3_ (SSMO) thin films deposited on LaAlO_3_ (LAO), SrTiO_3_ (STO), and (La_0.18_Sr_0.82_)(Al_0.59_Ta_0.41_)O_3_ (LSAT) single crystalline substrates are named as SSMO/STO, SSMO/LSAT, and SSMO/LAO, respectively. Reproduced with permission of [235]

Giri et al. [235] deposited epitaxial Sm_0.55_Sr_0.45_MnO_3_ thin films on LAO(001), LSAT(001), and STO(001) single crystal substrates by PLD technique, and the relationship between magnetocaloric effect and lattice strain induced by the substrates was investigated. The temperature-dependent |*∆S*_*M*_| values at different magnetic field calculated from *M*-*H* data for the Sm_0.55_Sr_0.45_MnO_3_ thin films grown on LAO (001), LSAT (001), and STO (001) single crystal substrates are shown in Fig. 36a–c, respectively. It is interesting to notice that the values of |*∆S*_*M*_| can be modulated by the lattice strain induced by the different substrates. The maximum value of |*∆S*_*M*_| was found to be ~ 10 J kg^−1^ K^−1^ for the Sm_0.55_Sr_0.45_MnO_3_ films grown on STO under a field change of 6 T. The inset of Fig. 36a shows the M-H loop measured at 10 K with increasing and decreasing magnetic fields of the films grown on STO. The field hysteretic loss is very less, which is a well characteristic of magnetic refrigeration. This low-field large magnetic entropy change in the thin film is mainly due to the rapid change of magnetization near the transition temperature in the easy magnetization plane. The specific heat (*C*_P_) data of the Sm_0.55_Sr_0.45_MnO_3_ film grown on LAO substrate is shown in Fig. 36d, which clearly shows a lambda-shaped anomaly close to *T*_C_. This is mainly arisen due to second-order magnetic phase transition. The peak temperature of *C*_P_ of the film matches well with *T*_C_ determined by dc magnetization measurement. The values of relative cooling power (RCP) are usually calculated for both the cases (near *T*_C_ and around *T*p), and several methods have been used to calculate the value of RCP. For example, in the first method, RCP-1 is calculated from the product of maximum peak value |*∆S*_*M*_| and the full width at half maximum, δ*T*_FWHM_, i.e., RCP-1 = $\left|{\Delta  S}_M^{Max}\right|$×δ*T*_FWHM_. In second method, RCP-2 is estimated from the maximum value (area) of the product |*∆S*_*M*_| ×Δ*T* under the |*∆S*_*M*_| vs. *T* curve. The Δ*S*_*M*_ versus *T* curve of the Sm_0.55_Sr_0.45_MnO_3_ films grown on STO at magnetic field of 6 T is shown in Fig. 36e. The bigger rectangular sketch and shaded area corresponds to RCP-1 and RCP-2, respectively. Inset in Fig. 36e shows the value of RCP-2 as a function of magnetic field for three different films. Figure 36f demonstrates the value of RCP-1 as a function of magnetic field for three different films. It can be observed that for both the temperature regime, the value of RCP increases with increasing the magnetic fields. An important observation is that the values of RCP are significantly larger around *T*_C_ than *T*_p_. Therefore, a material in the same refrigeration cycle with higher RCP is preferred as it would confirm the transport of a greater amount of heat in an ideal refrigeration cycle. The epitaxial Sm_0.55_Sr_0.45_MnO_3_ films grown on STO exhibit large MCE modulated by lattice strain; their larger |*∆S*_*M*_| and enhanced RCP with almost zero hysteresis loss make them ideal for magnetic refrigeration, providing an alternative approach in searching for energy efficient magnetic refrigerators.

#### Transport Properties

**Fig. 37:**
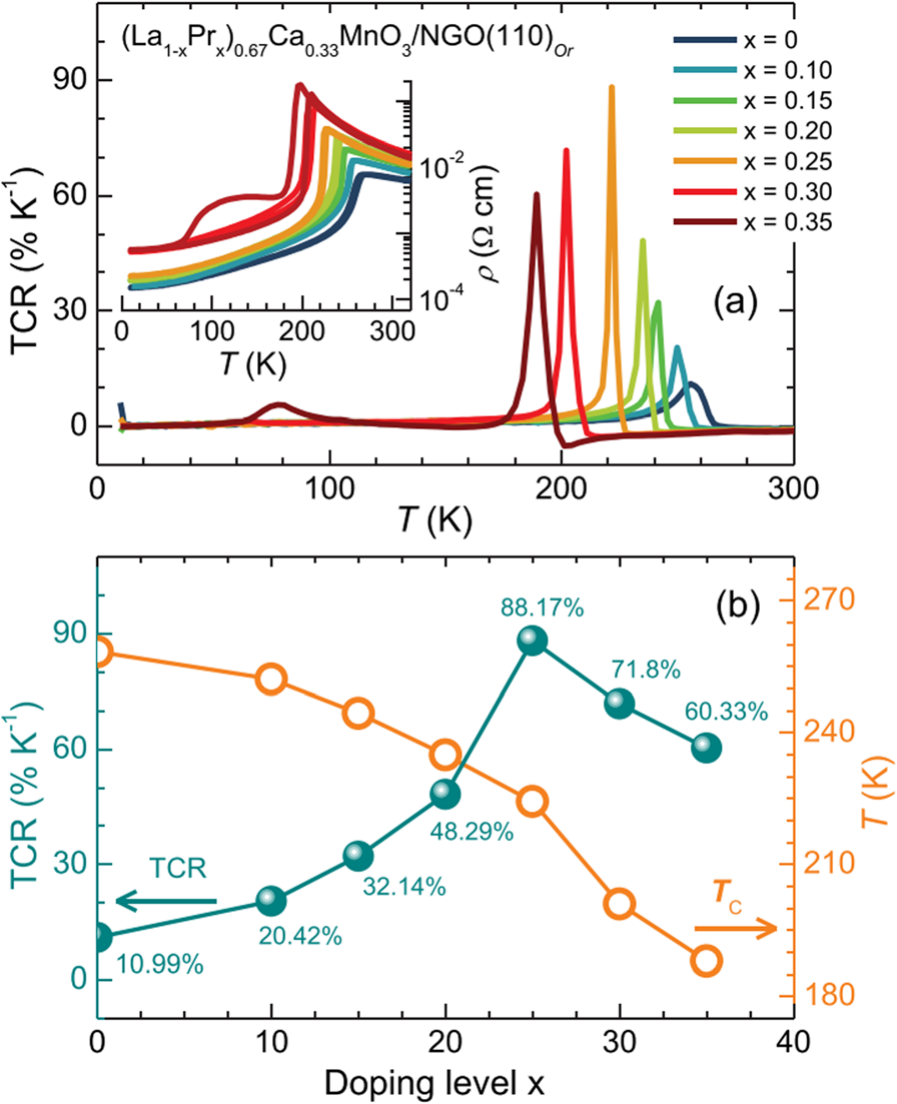
**a** Temperature dependence of TCR for the 30 nm (La_1-x_Pr_x_)_0.67_Ca_0.33_MnO_3_(LPCMO)/ NdGaO_3_(NGO) films at various doping levels (*x* = 0–0.35) at zero field. The inset shows the corresponding *ρ*-*T* curves. **b** *T*_C_ and TCR (peak value) as a function of the Pr doping level x, extracted from the *ρ*-*T* curves measured during cooling. Reproduced with permission of [236]

Chen et al. [236] investigated the transport properties of (La_1-x_Pr_x_)_0.67_Ca_0.33_MnO_3_ (0 ≤ × ≤ 0.35) films (with thicknesses from 9 to 60 nm) grown on NGO(110)_OR_ substrates by PLD. Their temperature coefficient of resistance (TCR, defined by (dρ/dT)/ρ, where ρ is the resistivity and T the temperature) is shown in Fig. 37a. The inset shows the corresponding ρ-T curves. The doping-level dependent T_C_ and TCR peak values are shown in Fig. 37b. Obviously, the monotonous reduction of *T*_C_ is accompanied by the increasing Pr-doping. TCR value greatly relies on the Pr-doping level *x*, which reaches the maximum value at 88.17% K^−1^ when doped at *x* = 0.25. The (La_1-x_Pr_x_)_0.67_Ca_0.33_MnO_3_ film at *x* = 0.25 may give a suitable FMM clusters size and distribution, leading to a sharp MIT with an optimized TCR. Dhakal et al. [105] also reported on the electrical properties of the (La_1-y_Pr_y_)_0.67_Ca_0.33_MnO_3_ (*y* = 0.4, 0.5, and 0.6) films with thickness of 30 nm grown on NGO(110) and STO(100) substrates by PLD. They found that the M-I transition temperature *T*_MI_ was decreased with increasing the Pr-doping concentration due to the reduction of average A-site cation radii <*r*_*A*_*>*. The transport properties of La_0.8-x_Pr_0.2_Sr_x_MnO_3_ (*x* = 0.1, 0.2, and 0.3) manganite films were also reported by Solanki et al. [118]. Their results confirmed the effects of Sr-concentration on transport properties. As smaller sized Pr^3+^ substituted at La-site in LaMnO_3_ results in the reduction in Mn-O-Mn bond angle from 180° making superexchange competitive with Zener double exchange, while substitution of Sr^2+^ in La_0.8-x_Pr_0.2_Sr_x_MnO_3_ system results in the increase in lattice parameters and the Mn-O-Mn bond angle toward 180°. Increase in Sr-concentration (from *x* = 0.1 to 0.3) enhances the e_g_ electron bandwidth due to larger size of Sr^2+^ ion, promoting the motion of more itinerant electrons between Mn^3+^ and Mn^4+^ which in turn suppresses resistivity and enhances T_P_ (metal–insulator/semiconductor transition temperature). In addition, due to substitution of Sr^2+^(x) at La-site, average grain size increases and grain boundary density decreases resulting in the suppression in scattering of e_g_ electrons which in turn increases *T*_P_.

**Fig. 38:**
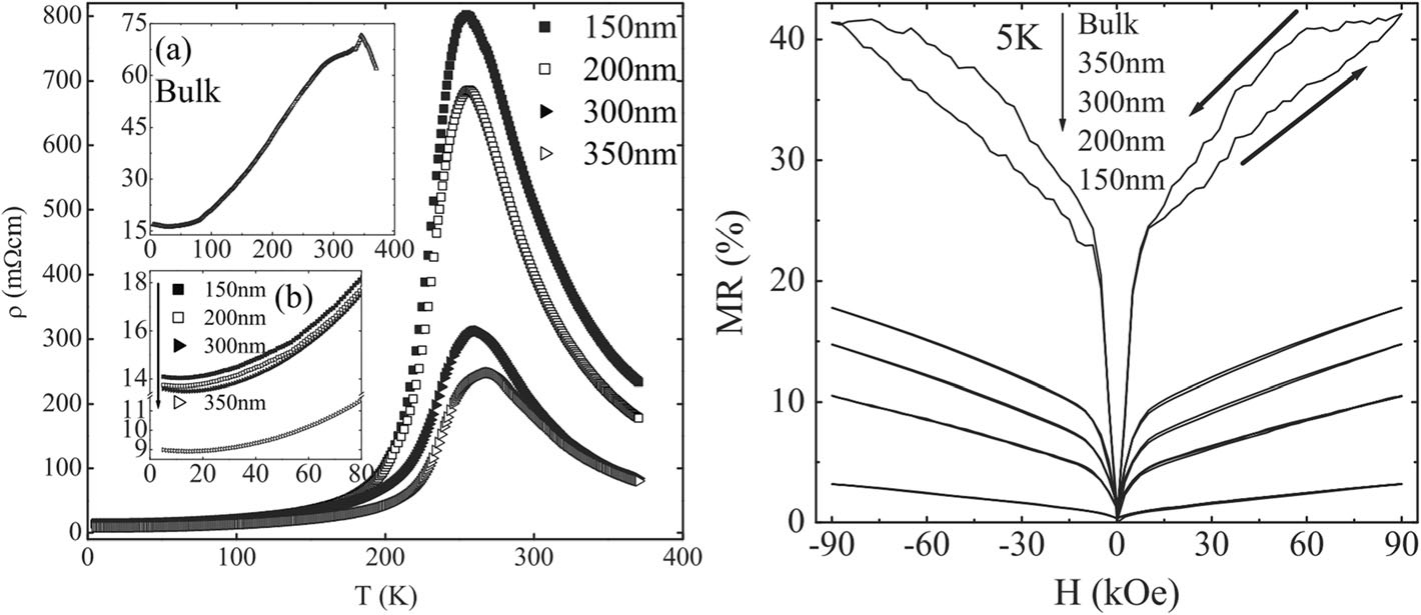
**a** ρ-T plots for the La_0.7_Pb_0.3_MnO_3_(LPMO)/LaAlO_3_(LAO) thin films grown by chemical solution deposition (CSD) method with various thicknesses. Inset (**a**): resistivity vs. temperature plot for bulk LPMO sample. Inset (**b**): enlarged view of low temperature (below 80 K) dependent resistivity behavior for the LPMO/LAO thin films having various thicknesses. **b** MR vs. H isotherms recorded at 5 K for the CSD grown LPMO/LAO thin films with different thicknesses and bulk LPMO sample. Reproduced with permission of [115]

The thickness-dependent transport properties of the La_0.7_Pb_0.3_MnO_3_ manganite films grown on LAO(100) single crystal substrates by CSD technique are also reported [115]. Figure 38a shows the *ρ*-*T* data of all the La_0.7_Pb_0.3_MnO_3_/LAO films under zero applied field. It is found that the *ρ* decreases and *T*_P_ increases up to 269 K (with thickness of 350 nm) as increasing the film thickness, which is ascribed to the total (in plane and out of plane) strain relaxation effect. Figure 38b shows low temperature MR behavior of various thickness films and bulk, the MR isotherms, at 5 K. It was found that bulk exhibits MR ~ 38% at 9 T, while the film only shows MR ~ 42% at room temperature. In addition, as the film thickness is increased, MR value at 5 K increases from 5% for 150 nm film to 18% for 350 nm film. This observation suggests the thickness-dependent microstructural effect on the transport and MR behavior of the La_0.7_Pb_0.3_MnO_3_/LAO films at low temperature under high fields. The thickness-dependent transport properties in the La_0.7_Sr_0.3_MnO_3_ thin films are also reported [116, 117, 237–239].

#### Optical Properties

**Fig. 39:**
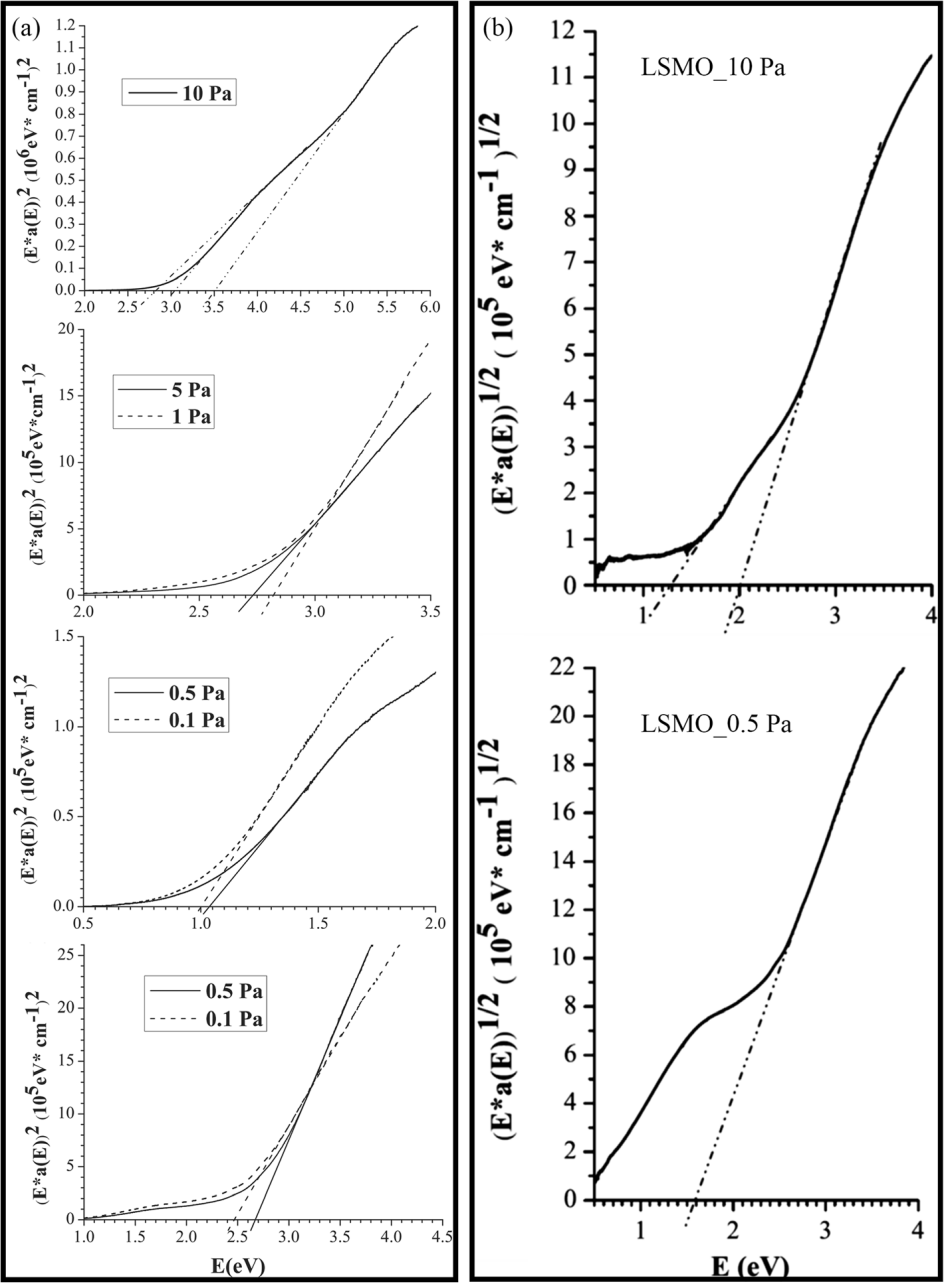
**a** Plots of (*Eα*(*E*))^2^ versus photon energy (*E*) of the LSMO films grown under different oxygen pressures. The direct transitions are identified by extrapolation of the straight line portions to energy axis. **b** Plots of (*Eα* (*E*))^1/2^ versus photon energy (*E*) of the LSMO films grown under oxygen pressures of 10 Pa and 0.5 Pa. Linear regions occur that are indicative of indirect transitions in the LSMO films grown under oxygen pressure of 10 and the amorphous nature for LSMO films grown under oxygen pressure of 0.5 Pa. Reproduced with permission of [240]

Cesaria et al. [240] reported the optical response of 200-nm-thick La_0.7_Sr_0.3_MnO_3-δ_ films, which were deposited by PLD on amorphous silica substrates at nearly 600 °C under different oxygen pressures (0.1, 0.5, 1, 5, and 10 Pa). A blue-shift of the transmittance-curve edge was observed as the p(O_2_) was increases from 1 to 10 Pa. That is ascribed to the changes of oxygen non-stoichiometry in the films, leading to larger Mn^4+^/Mn^3+^ ratios under higher oxygen pressure. In order to in-depth understand the optical response of the deposited films, the nature (direct or indirect) of the optical transitions in the films were investigated by plotting (*Eα*(*E*))^n^ versus the photon energy (*E*) for *n* = 1/2 and 2, where *α*(*E*) is the absorption coefficient. *n* is equal to 1/2 for indirect transition process or 2 for direct transition process. The graphs of (*Eα*(*E*))^2^ versus energy *E* for all the deposited films exhibit a linear regions, as shown in Fig. 39a, from which the direct energy gap values (*E*_g_) for the thin films can be estimated by extrapolation to the energy axis of the linear regions of the graphs. In the allowed direct transitions, the lowest energy one at nearly 1.0 eV was observed only for the films grown under oxygen pressures of 0.1 Pa and 0.5 Pa. That can be assigned to electronic excitations Mn^3+^(${\mathrm{e}}_g^1$)→Mn^3+^(${\mathrm{e}}_g^2$) from a bound state (owed to the lattice distortion around the Mn^3+^ ion) into a final state also bound by lattice distortions. The highest observed transition appeared at nearly 3.5 eV. Other transitions were yielded at intermediate energies for all the examined values of p(O_2_): optical transitions of at least 2.45 eV were observed with increasing energy as p(O_2_) was increased. The films grown under oxygen pressures of 10 Pa exhibited further transitions (at 3.04 eV and 3.50 eV), which can be assigned to transitions from O 2p states to the higher energy Mn^3+^(${\mathrm{e}}_g^2$) band. In the plots of (*Eα* (*E*))^1/2^ versus energy (*E*) of the films grown under the oxygen pressure of 10 Pa and 0.5 Pa, linear regions were also observed, as shown in Fig. 39b, which may be assigned to indirect transitions (phonon assisted transitions, i.e., phonon absorption and phonon emission) or indicative of amorphous nature. The occurrence of two adjacent linear dependences cab be interpreted as corresponding to phonon absorption and phonon emission processes leading to an indirect band gap. Indirect transitions are assigned to the films grown under oxygen pressures of 10 Pa, 5 Pa, and 1 Pa, and amorphous nature is detected in the films grown under oxygen pressures of 0.5 Pa and 0.1 Pa. Tanguturi et al. [241] reported the optical properties of Nd_0.7_Sr_0.3_MnO_3_ films grown on amorphous-SiO_2_ substrate. In the absorption coefficient spectra of the as-deposited and annealed films, a broad peak in the region *hv* < 2 eV was observed and beyond that they rose rapidly up to around 4 eV. Beyond 4 eV, no appreciable change in the spectrum was observed. The energy band gaps of the films were determined by plotting (*αE*)^2^ as a function of energy (*E*), which were determined to be 2.98 eV and 2.64 eV for the as-deposited and annealed films, respectively. Therefore, the amorphous film exhibits larger band gap as compared with the crystalline one. Such a large band gap value in amorphous phase is a known phenomenon [242].

### 3D Rare Earth-Doped Perovskite Manganite Oxide Nanostructures

**Fig. 40:**
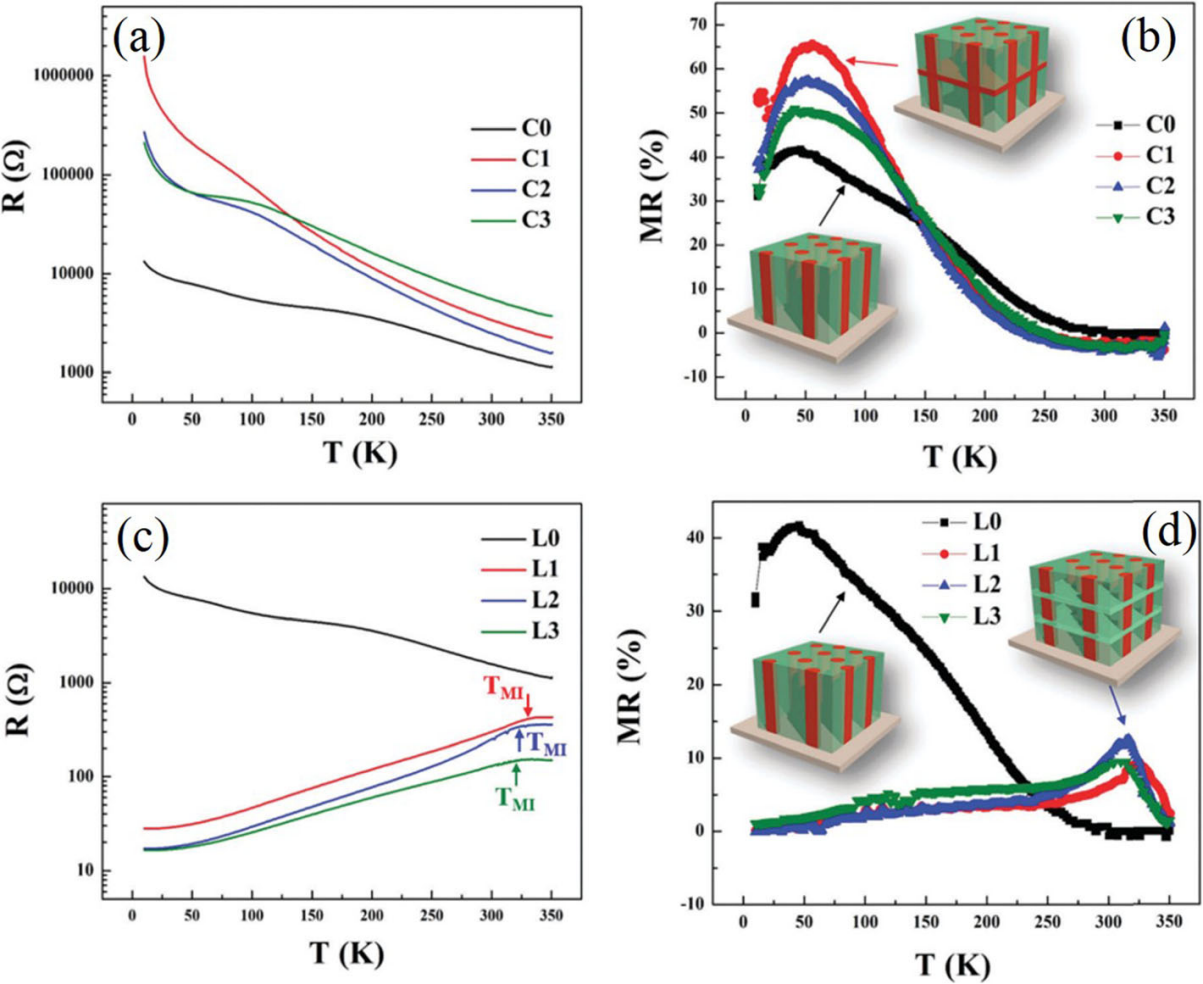
**a** R–T plots of 3D CeO_2_ framed nanocomposite thin films C0—C3. **b** The temperature dependence of MR for the nanocomposite thin films C0–C3. **c** R–T plots of 3D La_0.7_Sr_0.3_MnO_3_ (LSMO) framed nanocomposite thin films L0–L3. The arrows point out the metal-to-insulator transition temperature T_MI_ of L1–L3. **d** The temperature dependence of MR for the nanocomposite thin films L0 - L3 with the metal-to-insulator transition temperature T_MI_ marked for samples L1–L3. The single layer LSMO - CeO_2_ VAN thin films are named as C0 or L0, without LSMO or CeO_2_ as the interlayers. 3D CeO_2_ interlayered thin films with 1, 2, and 3 interlayers inserted in VAN structures are named as samples C1, C2, and C3, respectively. Similarly, 3D LSMO interlayered thin films with 1, 2, and 3 interlayers inserted in VAN are named as sample L1, L2, and L3, respectively. Reproduced with permission of [198]

Up to date, only a limited works on 3D rare earth-doped perovskite manganite oxide nanostructures are available. Here, an example of 3D rare earth-doped perovskite manganite oxide nanostructure is demonstrated, which is constructed by interlayering La_0.7_Sr_0.3_MnO_3_ (LSMO)–CeO_2_-based epitaxial vertically aligned nanocomposite (VAN) thin films with pure CeO_2_ (or LSMO) layers. This 3D strained framework nanostructures combine both the lateral strain by the layered structures and the vertical strain in the VAN, and thus maximize the 3D strain states in the systems, manipulating the electron transport paths in these systems. For example, in the 3D nanostructured LSMO–CeO_2_ VAN systems, the electrical transport properties can be effectively tuned from a 3D insulating CeO_2_ framework with integrated magnetic tunnel junction structures, to a 3D conducting LSMO framework by varying the types of the interlayers (i.e., CeO_2_ or LSMO) and the number of interlayers from 1 to 3 layers [198]. Figure 40 shows the transport properties of these 3D framed nanostructures. The temperature-dependent resistance (R–T) curves at zero-field are shown in Fig. 40a for samples C0–C3, and Fig. 40b depicts the temperature dependence of the MR (%) in the 3D-framed nanostructures C0–C3. It is observed that in Fig. 40a, the resistance is decreased with increasing temperature, indicating typical semiconductor behavior in C0–C3 due to the large portion of CeO_2_ introduced in the nanostructures (CeO_2_:LSMO ≥ 1:1 in C0–C3). The MR (%) of the films C0–C3 is increased at first and then reduced as the temperature increasing from low temperature to room temperature. Therefore, a MR peak is observed around 50 K. It is also noticed that the 3D CeO_2_ frameworks could enhance the overall MR properties; for example, the MR peak value is increased from 40% (C0) to 51% (C3), 57% (C2), and maximized at 66% (C1). Such an enhancement can be ascribed to the 3D CeO_2_ framework not only tailoring the out-of-plane strain of the LSMO phase but also building up the 3D tunneling framework for the electron transport. The relatively lower MR (%) in C2 and C3 samples compared to C1 is possibly related to the surface roughness observed in both samples where the 3D insulating framework might not be effective in the top layers. In contrast to the C1–C3 samples, a metallic behavior is observed in the L1–L3 samples with a 3D LSMO framework, as shown in Fig. 40c. The resistances are gradually increased from 10 to 350 K with a M–I transition temperature (*T*_MI_) at ~ 325 K. Such a metallic behavior is associated with the high composition of LSMO in L1–L3 and the 3D interconnected conductive LSMO frames built in the composite films L1–L3. Meanwhile, the resistance of the composite films L1–L3 decreases with inserting more lateral LSMO interlayers over the entire temperature regime. The LSMO interlayers interconnect with the vertical LSMO domains forming a conductive 3D frame in the film. Thus, the tunneling MR effect is effectively reduced. Figure 40d demonstrates the temperature dependence of MR for the nanocomposite thin films L0–L3 with the M–I transition temperature (*T*_MI_) marked for samples L1–L3. It is observed that such L1–L3 structures enable higher MR values at higher temperatures, e.g., 13% at 316 K in sample L2, which is a dramatic MR value improvement compared to C0–C3 and the previous reports at higher temperatures (e.g., near room temperature). Based on the above observations, it is clear that magnetic tunneling junctions (MTJ) of the LSMO/CeO_2_/LSMO and their geometrical arrangement in the 3D framework nanostructures are very important for enhancing the low-field MR properties. In C1–C3 samples, there are effective vertical and lateral MTJ structures integrated in the system by incorporating CeO_2_ interlayers in the VAN system, such 3D insulating frameworks effectively maximize the 3D magnetic tunneling effect and lead to a record high MR% in the LSMO based systems. This 3D strain framework concept opens up a new avenue to maximize the film strain beyond the initial critical thickness and can be applied to many other material systems with strain-enabled functionalities beyond magneto-transport properties.

## Applications of Rare Earth-Doped Perovskite Manganite Oxide Nanostructures

### Rare Earth-Doped Perovskite Manganite Oxide Nanoparticles

#### Magnetic Refrigeration

Magnetocaloric effect (MCE) makes magnetic materials attractive for potential applications in magnetic refrigeration. As compared to the conventional gas compression technology, the magnetic refrigeration technology offers many advantages, such as no use of any gasses or hazardous chemicals, low energy consumption, and low capital cost [243–245].

Mahato et al. [246] synthesized La_0.7_Te_0.3_MnO_3_ nanoparticles with average particle size of 52 nm where a large magnetic entropy change of 12.5 J kg^−1^ K^−1^ was obtained near *T*_C_ for a field change of 50 kOe. These results confirmed the application for magnetic refrigeration. Yang et al. [247] reported that for La_0.7_Ca_0.3_MnO_3_ nanoparticles with average size of 30 and 50 nm, their maximum magnetic entropy changes at 15 kOe applied field were 1.01 and 1.20 J kg^−1^ K^−1^, respectively, indicating that the La_0.7_Ca_0.3_MnO_3_ nanoparticles could be considered as a potential candidate for magnetic refrigeration applications at room temperature. Phan et al. [248] demonstrated strong enhancement of both the MCE and refrigerant capacity in the nanostructured mixed phase manganite of La_0.35_Pr_0.275_Ca_0.375_MnO_3_ (with an average particle size of 50 nm). Compared to other candidates such as Pr_0.65_(Ca_0.7_Sr_0.3_)_0.35_MnO_3_ (~ 67 nm) [249], the higher entropy change ($-{\Delta  \mathrm{S}}_{\mathrm{M}}^{\mathrm{M}\mathrm{ax}}$) and RC (refrigerant capacity) values were achieved in the nanocrystalline sample.

#### Biomedical Applications

Magnetic nanoparticles offer some attractive possibilities in biomedicine. First, they have controllable sizes ranging from a few nanometers up to tens of nanometers, which places them at dimensions that are smaller than or comparable to those of a cell (10–100 μm), a virus (20–450 nm), a protein (5–50 nm), or a gene (2 nm wide and 10–100 nm long). This means that they can “get close” to a biological entity of interest. Indeed, they can be coated with biological molecules to make them interact with or bind to a biological entity, thereby providing a controllable means of “tagging” or addressing it. Second, the nanoparticles are magnetic, which means that they obey Coulomb’s law, and can be manipulated by an external magnetic field gradient. This “action at a distance,” combined with the intrinsic penetrability of magnetic fields into human tissue, opens up many applications involving the transport and/or immobilization of magnetic nanoparticles, or of magnetically tagged biological entities. In this way, they can be made to deliver a package, such as an anticancer drug, or a cohort of radionuclide atoms, to a targeted region of the body, such as a tumour. Third, the magnetic nanoparticles can be made to resonantly respond to a time-varying magnetic field, with advantageous results related to the transfer of energy from the exciting field to the nanoparticle. For example, the particle can be made to heat up, which leads to their use as hyperthermia agents, delivering toxic amounts of thermal energy to targeted bodies such as tumours; or as chemotherapy and radiotherapy enhancement agents, where a moderate degree of tissue warming results in more effective malignant cell destruction. These, and many other potential applications, are made available in biomedicine as a result of the special physical properties of magnetic nanoparticles [250].

Bhayani et al. [251] report, for the first time, immobilization of commonly used biocompatible molecules on La_1-x_Sr_x_MnO_3_ nanoparticles, namely bovine serum albumin and dextran. Such bioconjugated nanoparticles have a tremendous potential application, especially in the field of biomedicine. Daengsakul et al. [61, 62] reported the cytotoxicity of La_1-x_Sr_x_MnO_3_ nanoparticles with *x* = 0, 0.1, 0.2, 0.3, 0.4, and 0.5 evaluated with cell NIH 3T3. The result showed that the La_1-x_Sr_x_MnO_3_ nanoparticles were not toxic to the cells. This will be useful for medical applications. Similar studies about the toxicity of the nanoparticles were performed by Zhang et al. for safe biomedical applications [252].

Magnetic resonance imaging (MRI) represents a powerful imaging method commonly utilized in clinical practice. The method shows excellent spatial resolution, which is very suitable not only for examination of human bodies but also for detailed anatomical studies of animal models in vivo in biological research. On the other hand, the sensitivities of other techniques, such as optical methods, single-photon emission computed tomography or positron emission tomography, are much higher. Thus, the design and synthesis of so called dual or multimodal probes is an important field. The combination of both respective approaches utilizing only one dual probe, e.g., magnetic nanoparticles tagged with fluorescent moieties, establishes a very useful method for bioimaging. Moreover, fluorescent magnetic nanoparticles are promising materials for other medical applications, where the same tool might be used either for diagnostics or for therapy, like for magnetic hyperthermia and optically driven surgery. The positioning of the magnetic cores with the external magnetic field could be used in cell micromanipulation [253–255]. Kačenka et al. [256] reported the potential of magnetic nanoparticles based on the La_0.75_Sr_0.25_MnO_3_ perovskite manganite for MRI. Fluorescent magnetic nanoparticles based on a perovskite manganite La_0.75_Sr_0.25_MnO_3_ core coated with a two-ply silica layer were synthesized and thoroughly characterized in order to prepare a novel dual MRI/fluorescence probe with enhanced colloidal and chemical stability. Viability tests show that the complete particles are suitable for biological studies.

In recent years, magnetic nanoparticles have been used in magnetic hyperthermia, referring to the introduction of ferromagnetic or super-paramagnetic particles into the tumor tissue. The magnetic nanoparticles create heat that can be used to treat cancer when they are placed in alternating magnetic fields. The La_1-x_Sr_x_MnO_3_ nanoparticles for hyperthermia applications are studied in details [257–260].

#### Catalysts

Research in environmental catalysis has continuously evolved over the last two decades owing to the necessity of obtaining worthwhile solutions to environmental pollution problems. The development of innovative environmental catalysts is a crucial factor toward the purpose of determining new sustainable manufacturing technologies. Rare earth perovskite manganites attract notable attention of explorers due to their high catalytic activity in numerous redox reactions [261, 262].

Oxygen electrocatalysis is one of the key processes limiting the efficiency of energy conversion devices such as fuel cells, electrolysers, and metal-air batteries. In particular, the oxygen reduction reaction (ORR) is commonly associated with slow kinetics, requiring high overpotentials, and high catalyst loadings. Celorrio et al. [263] reported the effect of tellurium (Te) doping on the electrocatalytic activity of La_1-x_Te_x_MnO_3_ nanoparticles with an average diameter in the range of 40 - 68 nm toward the oxygen reduction reaction.

Carbon monoxide is a colorless, odorless, and tasteless gas that is slightly lighter than air. It is toxic to humans and animals when encountered in higher concentrations. The catalytic oxidation of CO is utilized in various applications, e.g., indoor air cleaning, CO gas sensors, CO_2_ lasers, and automotive exhaust treatment. The structural and catalytic properties of La_1-x_(Sr or Bi)_x_MnO_3_ samples with *x* = 0.0, 0.2 or 0.4 for CO oxidation, are investigated [264].

Volatile organic compounds (VOCs), emitted from many industrial processes and transportation activities, are considered as great contributors to the atmospheric pollution and dangerous for their effect on the human health [265]. From an economical point of view, compared to an incineration process, catalytic combustion is one of the most interesting technology for the destruction of emissions of VOCs. Blasin-Aubé et al. [266] reported that the La_0.8_Sr_0.2_MnO_3+x_ perovskite-type catalyst is highly active in the oxidative destruction of VOCs, especially for oxygenated compounds.

The possibility of catalytic activity enhancement in NO reduction is also studied [267]. Ran et al. synthesized the Ce-doped PrMnO_3_ catalysts and investigated the effect of cerium doping on the catalytic properties of Ce-doped PrMnO_3_ catalysts. Their results showed that in the case of the Ce-doped series with lower doping ratio, most of the Ce^4+^ ions were introduced into the A-site to form perovskite-type oxides with some additional ceria. The oxidation state of manganese was more easily affected by the addition of cerium and more vacancies might arise at the A-site due to the structural limit of the oxide. High catalytic activity in NO reduction might be caused by the presence of oxygen vacancies and the relative ease of oxygen removal. Besides, ceria could also adsorb oxygen to sustain the reduction of NO.

#### Solid Oxide Fuel Cells

Solid oxide fuel cells (SOFCs) have become of great interest as a potential economical, clean, and efficient means of producing electricity in a variety of commercial and industrial applications. Its major advantages include high efficiency, potential for cogeneration, modular construction, and very low pollutant emissions. Lanthanum manganite-based oxides, e.g., La_1-x_Ca_x_MnO_3_ and La_1-x_Sr_x_MnO_3_, are promising materials as cathodes, because of their high electrical conductivity and good compatibility with yttria-stabilized zirconia (YSZ).

For example, nano-sized (La_0.85_Sr_0.15_)_0.9_MnO_3_ and Y_0.15_Zr_0.85_O_1.92_ (LSM–YSZ) composite with 100–200 nm in diameter was co-synthesized by a glycine–nitrate process (GNP) [268]. Alternating current impedance measurement revealed that the co-synthesized LSM–YSZ electrode shows lower polarization resistance and activation energy than the physically mixed LSM–YSZ electrode. This electrochemical improvement was attributed to the increase in three-phase boundary and good dispersion of LSM and YSZ phases within the composite. Lay et al. [269] synthesized the Ce-doped La/Sr chromo-manganite series (Ce_x_La_0.75- x_Sr_0.25_Cr_0.5_Mn_0.5_O_3_ with *x* = 0, 0.10, 0.25, and 0.375) as potential SOFC anode or solid oxide electrolyzer cell (SOEC) cathode materials. All those materials are stable in both elaboration and operating conditions of an SOFC anode, and they are also stable in steam electrolysis of cathodic conditions in SOEC. Besides, the possibility of A_2-x_A′_x_MO_4_ (A = Pr, Sm; A′ = Sr; M = Mn, Ni; *x* = 0.3, 0.6) as a cathode of SOFC was investigated by Nie et al. [270].

Besides being used as cathodes in solid oxide fuel cells, rare earth-doped perovskite manganite oxides (Ln_x_A_1-x_MnO_3_) also exhibit high potential for being used as redox materials for solar thermochemical fuel production from thermochemical H_2_O/CO_2_ splitting [271]. It was reported that substituted lanthanum manganite perovskite was one of the most suitable candidates among the perovskite family, owing to its unique redox properties [272]. To further improve the redox properties of these materials, Nair and Abanades [273] performed a systematic study to investigate the effects of synthesis methods on the redox efficiency and performance stability for CO_2_ splitting. They synthesized single-phase La_x_Sr_1-x_MnO_3_ (LSMO) by using various technical routes such as solid-state reactions, pechini process, glycine combustion, or glucose-assisted methods, and found that the materials synthesized by the pechini method exhibited the highest reactivity among the series, and a stable CO production of ~ 260 μmol g^−^1 was achieved at *x* = 0.5. They also found that the substitutions of Y/Ca/Ba at A-site and Al/Fe at B-site in (La,Sr)MnO_3_ did not enhance the redox cycling capability as compared with LSMO. It was observed that Sr was the best A-site substituent and the presence of single Mn cation alone in B site was the most suitable option for promoting CO_2_-splitting activity. Furthermore, the addition of promotional agents and sintering inhibitor such as MgO and CeO_2_ without altering the La_0.5_Sr_0.5_MnO_3_ composition could improve the CO_2_-splitting activity. For an overview on the recent progress on solar thermochemical splitting of water to generate hydrogen, we refer to other review articles **[**274–276].

### 1D Rare Earth-Doped Perovskite Manganite Oxide Nanostructures

#### Catalysts

In the catalytic combustion of methane, the perovskite manganites nanoparticles generally lose their activities due to the severe sintering under such a high-output and high-temperature reaction. As a consequence, designing of highly reactive and stable catalysts has been an interesting research direction in heterogeneous catalysis. Recently, some results indicate that the catalytic properties of the catalysts could be improved by controlling their morphologies and structure. For example, SrCO_3_ nanowires showed higher activity for ethanol oxidation than the nanoparticles [277]. Besides, CeO_2_ nanorods were more reactive for CO oxidation than the corresponding nanoparticles [278]. However, catalytic properties of 1D Rare earth-doped perovskite manganite have been scarcely reported. Teng [279] reported the hydrothermal synthesis of La_0.5_Sr_0.5_MnO_3_ nanowires, and the stability and the activity for methane combustion of La_0.5_Sr_0.5_MnO_3_ nanowires were also investigated. The results showed that after being calcined for a long time, the nanowires showed a higher stability as compared with the La_0.5_Sr_0.5_MnO_3_ nanoparticles. The nanowire catalyst maintained a higher catalytic activity for methane combustion. The photocatalytic activity of La_1-x_Ca_x_MnO_3_(*x* ≈ 0*.*3) nanowires synthesized by hydrothermal method was also investigated by Arabi et al. [230]. The results revealed that La_0*.*68_Ca_0.32_MnO_3_ nanowires exhibited sufficient photocatalytic activity for degradation of methylene blue solution under visible-light irradiation.

#### Solid Oxide Fuel Cells

Up to now, various approaches have been suggested to fabricate LSM/YSZ composite cathodes for SOFCs. Several researches have shown that electrode microstructure (i.e., particle size, pore size, and porosity) has a strong influence on the value of the area specific resistance (ASR) [280–282]. Da and Baus synthesized La_0.65_Sr_0.3_MnO_3_ (LSM) nanorods through a simple hydrothermal reaction. It is worth noting that the ASR values in this work are substantially lower than most of the former reports available in the literature [268, 283–288]. They pointed out that the promising performance of the nanostructured LSM cathodes was attributed to the optimized microstructure, i.e., high surface area, small grain size, and good inter-granular connectivity, which make it a potential candidate for intermediate temperature SOFC application. In addition, nano-tube structured composite cathodes were also investigated [283]. La_0.8_Sr_0.2_MnO_3-δ_/Zr_0.92_Y_0.08_O_2_ (LSM/YSZ) composite nano-tubes are co-synthesized by a pore wetting technique as a cathode material for SOFCs. The as-prepared nanostructured composite cathode shows low ASR values of 0.17, 0.25, 0.39, and 0.52 Ω cm^−2^ at 850, 800, 750, and 700 °C, which is mainly due to small grain size, homogeneous particle distribution and fine pore structure of the material.

#### Magnetic Memory Devices

The elaboration of submicron MR read heads and high-sensitive elements of non-volatile memories (MRAM) passes through patterning processes that are commonly used in the semiconductor industry. The planar processes for thin-film patterning are based on two main steps: (i) the pattern definition in photon or electron sensitive polymer (resist) by lithography and (ii) the transfer of these nanostructures in the manganite film using dry etching [163]. Conventional UV lithography is traditionally used to get patterns higher than one micron in size; however, patterning at dimensions lower than 50 nm needs high-resolution techniques such as scanning electron beam lithography (SEBL), X-ray lithography (XRL), or NI [289, 290]. At present, ultimate resolution limits of SEBL and XRL are well known [291, 292] and the electron PMMA (polymethylmethacrylate) resist allows replications below 20 nm. After nanolithography, the pattern transfer can be achieved using direct etching with the resist as mask, or using a metallic lift-off process followed by etching. The lift-off process is the preferred method for manganite etching since these CMR oxides are very hard materials compared to metals.

A magnetic domain wall separates two oppositely polarized magnetic regions, and a number of data storage schemes based on domain walls in magnetic nanowires have been proposed [293, 294]. In the race-track memory, each magnetic domain wall represents a data bit [293]. During the write operation, the domain wall is moved by external magnetic field or spin transfer torque [295–297]. To read a bit, GMR or TMR type devices are used to detect the stray field from the domain wall. To utilize such a scheme, it is critical to controllably create domain walls. Magnetic nanowires with an artificial pinning center, such as notches [293], bent conduits [298], and narrow rings [299], can serve this purpose. In perovskite manganite nanostructures, various types of domain patterns such as stripes [300], bubbles [301], and checker-boards [302] have been reported. For example, Wu et al. [303] reported on the perpendicular stripe magnetic domains in La_0.7_Sr_0.3_MnO_3_ nanodots. Takamura et al. [304] reported flower-shaped, flux closure domain, and vortex structures in patterned manganites created by Ar+ ion milling. Mathews et al. [305] reported successful fabrication of La_0.67_Sr_0.33_MnO_3_ nanowires on NdGaO_3_ substrate by using interference lithography. It was demonstrated that not only the shape anisotropy but also the substrate induced anisotropy play important roles in determining the magnetic easy axis in these manganite nanostructures. In spite of challenges in controlling magnetic domain walls in perovskite manganite oxide nanowires, several groups have reported current induced domain wall motion in perovskite manganite oxide materials such as La_0.7_Sr_0.3_MnO_3_ and La_0.67_Ba_0.33_MnO_3-δ_. By using FIB milling, Ruotolo et al. [306] and Céspedes et al. [139] patterned La_0.7_Sr_0.3_MnO_3_ into nanowires containing notches as the domain wall pinning centers. The MR measurements confirm the current induced domain wall depinning with a critical current density of 10^11^ A/m^2^. Liu et al. [307] reported current dependent low-field MR effect in La_0.67_Sr_0.33_MnO_3_ nanowires with constrictions and they ascribed this effect to the spin polarized bias current. In a similar constricted La_0.67_Ba_0.33_MnO_3-δ_ nanowire, Pallecchi et al. [308] observed magnetic field and DC bias current dependent asymmetric resistance hysteresis, which was also connected to the effect spin transfer torque. Surprisingly, the threshold current was found to be in the range of 10^7^–10^8^ A/m^2^, much smaller than the typical current (10^11^ A/m^2^) needed for moving domain walls in metals [309]. A number of possibilities, such as stronger spin torque due to half metallicity, Joule heating assistance, and spin wave excitation, may contribute to such a drastic reduction in the threshold current.

#### Spintronic Devices

In terms of perovskite manganites, the large MR and the great tunability of CMR oxides are promising for magnetic recording, spin valve devices, and magnetic tunnelling junctions [310–314]. However, there are several obstacles related to perovskite manganites in nanodevice applications. First, the spin polarization of manganites decays rapidly with temperature. Second, the defect chemistry and the stoichiometry–property correlation in perovskite manganites are quite complex [315, 316]. Third, the physical properties of interfaces in manganite-based devices remain elusive [317, 318]. Finally, there is the urgent need for developing suitable device processing techniques.

The spintronic devices exhibit prominent advantages, such as nonvolatility, increased processing speed, increased integration densities, and reduced power consumption. The significance and value of spintronics lies in the study of active control of carrier spin, and then the development of new devices and technologies, for example, spin transistors, spin-FET, spin-LED, spin-RTD, spin filters, spin modulators, reprogramming gate circuit.

Nanowires have been successfully incorporated into nanoelectronics [319], and naturally they are envisaged as ideal building blocks for nanoscale spintronics. For perovskite manganite oxides, nanowires and related heterostructures hopefully can enhance the Curie temperature [82] and the low-field MR [75, 320]. Another promising research topic is anisotropic magnetoresistance (AMR) which is a result of spin-orbital interaction and has important applications in magnetic field detection and data storage [321]. Significant magnetic anisotropy has been observed in perovskite manganite oxide nanowires [322]. In addition, the morphology of nanowire can be modified by annealing or growing on engineered substrates [323], which could significantly affect their properties. It is expected that more efforts will be made to explore the anisotropic transport properties of perovskite manganite oxide nanowires and to gain deeper understanding of low-dimensional spin-dependent transport properties.

Spin valves based on perovskite manganite oxide nanowires are also reported. Gaucher et al. [324] successfully fabricated La_2/3_Sr_1/3_MnO_3_ nanowires with the smallest width of 65 nm by combining electron beam lithography and ion beam etching. They showed that the electronic transport properties of these perovskite manganite oxide nanowires are comparable to those of unpatterned films.

### 2D Rare Earth-Doped Perovskite Manganite Oxide Nanostructures

#### Magnetic Memory Devices

Typically, micrometer-sized La_0*.*7_Sr_0*.*3_MnO_3_ dots with smooth edge and surface are etched in the corresponding films by such lift-off process. Ruzmetov et al. [135] fabricated regular arrays of epitaxial perovskite La_2/3_Sr_1/3_MnO_3_ magnetic nanodots by PLD combined with electron beam lithography and argon ions exposure. The diameters of these dots are less than 100 nm with a height about 37 nm. These perovskite magnetic nanodots maintain their crystallinity, epitaxial structure, and ferromagnetic properties after the fabrication process, which have promising applications in magnetic massive memory devices.

**Fig. 41:**
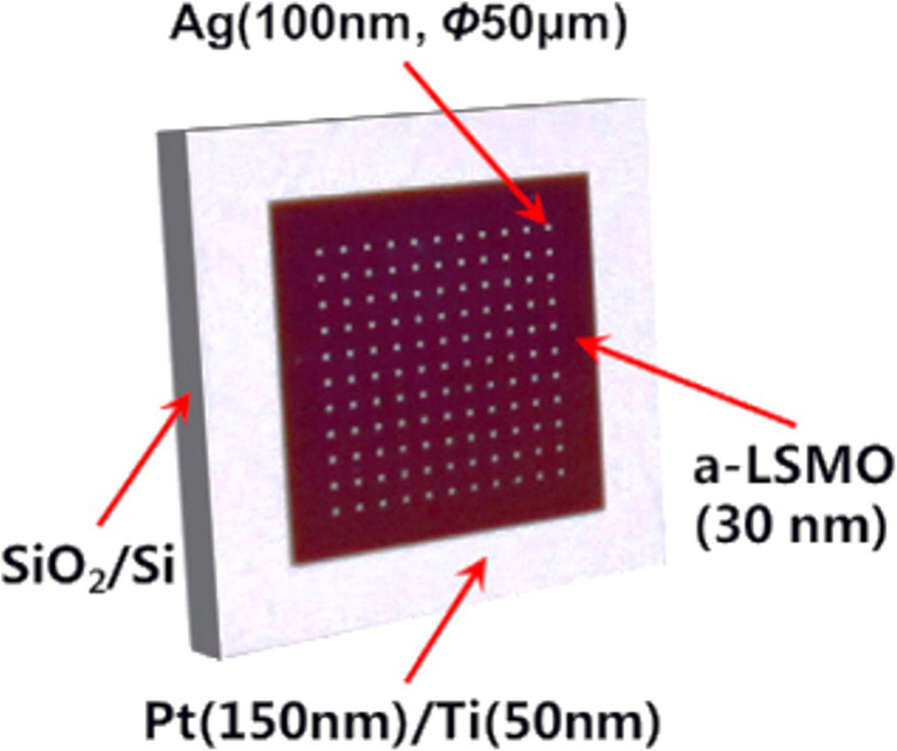
Schematic of the Ag/La_0.79_Sr_0.21_MnO_3_ (*a*-LSMO)/Pt memory device. Reproduced with permission of [325]

Liu et al. [325] demonstrated a new programmable metallization cell based on amorphous La_0.79_Sr_0.21_MnO_3_ thin films for nonvolatile memory applications. The schematic diagram of the metallization cell is shown in Fig. 41, where amorphous La_0.79_Sr_0.21_MnO_3_ thin films are deposited on the Pt/Ti/SiO_2_/Si substrates by rf magnetron sputtering. The Ag/amorphous La_0.79_Sr_0.21_MnO_3_/Pt cell exhibited reversible bipolar resistive with a R_OFF_/R_ON_ ratio (> 10^2^), stable write/erase endurance (> 10^2^ cycles) with resistance ratio over 10^2^, and stable retention for over 10^4^ s. Such a sandwiched device may be a promising candidate for future nonvolatile memory applications.

Hoffman et al. [326] also tested the non-volatile memories using an electric-field-induced M–I transition in the PbZr_0.2_Ti_0.8_O_3_/La_1-x_Sr_x_MnO_3_ (PZT/LSMO), PZT/La_1-x_Ca_x_MnO_3_ (PZT/LCMO), and PZT/La_1-x_Sr_x_CoO_3_ (PZT/LSCO) devices. To study the switching speed of the Mott transition field effect devices, they fabricated a series of devices where the room temperature RC-time constants varied from 80 ns to 20 μs. They found the circuit RC- time constant limited the switching speed of devices down to 80 ns, offering the opportunity for faster operation though device scaling. Room temperature retention characteristics show a slow relaxation, with more than 75% of the initial polarization maintained after 21 days. These Mott transition field effect devices have promising potential for future non-volatile memory applications.

#### Spintronic Devices

Spin valves may be the most influential spintronic devices which have already found applications in magnetic data storage industry. Its basic working principle is the GMR effect [327, 328], where the resistance of a FM/NM (non-magnetic)/FM multilayers depends on the relative alignment between the two FM layers. Most GMR research focuses on transition metals, but spin valves have also been realized in oxides, in particular FM manganites [329, 330]. A related concept is the magnetic tunnel junction (MTJ) [331, 332], which also has vast applications in nonvolatile magnetic memory devices. The basic difference between spin valve and MTJ lies in the middle spacer layer, which needs to be insulating for MTJ, whereas for spin valve, this layer is conducting. A number of efforts have been devoted to create oxide based MTJ devices so far, especially due to the 100% spin polarization in several FM oxides, such as La_1-x_Sr_x_MnO_3_ with *x* ~ 0.33, CrO_2_ and Fe_3_O_4_. Lu et al. [333] and Sun et al. [334] first fabricated all-oxide MTJ device with La_0.67_Sr_0.33_MnO_3_ and SrTiO_3_ as FM and insulating layer, respectively. Subsequently, a record tunneling magnetoresistance (TMR) ratio of 1850% was reported in 2003 by Bowen et al. [335]. Despite these promising results, a major issue for the oxide based MTJ devices is that the working temperature is often lower than the room temperature, generally ascribed to the degraded interfaces [336, 337].

#### Magnetic Sensors

The application of perovskite manganite CMR thin films to magnetic sensors at room temperature has been considered. Compared to magnetoresistive sensors using permalloy films, the field coefficient of resistance (dR/dH)/R is much smaller, typically of about (10^−2^–10^−1^)% per mT. However, they can operate over a wide field range and their characteristics should be maintained at submicron lateral size, since the CMR mechanism does not involve large scale entities such as magnetic domains and walls. By means of magnetic flux concentration with soft ferrite poles, the field coefficient of resistance can be increased up to 4% per mT [338]. Other applications of CMR of La_0.67_Sr_0.33_MnO_3_ thin films to position sensors and to contact-less potentiometers are presently investigated [339]. The basic idea is to exploit the large resistance variation induced by the stray field of a permanent magnet in Sm-Co or Nd-Fe-B alloys.

In recent years, there has been growing demand for sensitive yet inexpensive infrared detectors for use in a variety of civilian, industrial, and defense applications such as thermal imaging, security systems and surveillance, night vision, biomedical imaging, fire detection, and environmental detection. The material for bolometric applications should possess a high temperature coefficient of resistivity (TCR), which enables small temperature variations caused by absorbed infrared radiation (IR) generate a significant voltage drop across the bolometer. High TCR in recently discovered CMR manganese oxides in the vicinity of metal-to-semiconductor phase transition makes them suitable for thermometer and bolometer applications. For example, Lisauskas et al. [340] reported that the epitaxial submicron thick perovskite manganite La_0.7_(Pb_0.63_Sr_0.37_)_0.3_MnO_3_ films deposited on LAO single crystal by PLD exhibited high value of TCR (= 7.4% K^−1^ @ 295 K). Choudhary et al. [341] synthesized the polycrystalline/amorphous films with mixed-valence manganites (e.g., La_0.7_Ca_0.3_MnO_3_, La_0.5_Sr_0.5_MnO_3_, La_0.5_Ba_0.5_MnO_3_, and (La_0.6_Pr_0.4_)_0.67_Ca_0.33_MnO_3_) by PLD at low temperature (450 °C) on single crystal (001) silicon substrate. These films are evaluated for uncooled bolometric applications.

#### Solid Oxide Fuel Cells

Lussier et al. [342] identified a mechanism whereby the strain at an interface is accommodated by modifying the chemical structure of the SOFC material to improve the lattice mismatch and distribute the strain energy over a larger volume (thickness), concentrate on two particular manganite compounds, La_2/3_Ca_1/3_MnO_3_ and La_1/2_Sr_1/2_MnO_3_ thin films.

### 3D Rare Earth-Doped Perovskite Manganite Oxide Nanostructures

Recently, several 3D rare earth-doped perovskite manganite oxide nanostructures such as 3D (La_0.275_Pr_0.35_Ca_0.375_)MnO_3_ nanobox array structures (145), 3D strained LSMO–CeO_2_ VAN nanostructures (198) fabricated by PLD technique are reported. It was found that 3D (La_0.275_Pr_0.35_Ca_0.375_)MnO_3_ nanobox array structures exhibited an insulator-metal transition at higher temperature than that in the corresponding thin film, which provided a new way to tune the physical properties of CMR oxide 3D nanostructures. This enables 3D (La_0.275_Pr_0.35_Ca_0.375_)MnO_3_ nanobox array structures to find promising application in oxide nanoelectronics by making full use of the huge electronic/spintronic phase transition. The 3D framework of LSMO–CeO_2_ VAN nanostructures combine not only the lateral strains from the layered structures but also the vertical strain from the VAN, and thus maximize the 3D strain states in the systems, controlling the electron transport paths. This new 3D framed design provides a novel approach in maximizing film strain, enhancing strain-driven functionalities, and manipulating the electrical transport properties effectively. At present, the applications of 3D rare earth-doped perovskite manganite oxide nanostructures in the fields of oxide nanoelectronics, spintronics, and solar energy conversion are still in their infancy; thus, many problems remain unsolved and technical challenges lie ahead. In this direction, there is a long way to walk on before the commercialization of rare earth-doped perovskite manganite oxide nanostructures.

## Conclusions and Perspectives

In this work, we have discussed the recent advances in the fabrication, structural characterization, physical properties, and functional applications of rare earth-doped perovskite manganite oxide nanostructures. It is our aim that we have captured all the excitements achieved in the development of rare earth-doped perovskite manganite oxide nanostructures used for microelectronic, magnetic, and spintronic nanodevices, providing some useful guidelines for the future researches. In spite of great progress made in the past two decades, considerable effort is highly required to realize the practical applications of rare earth-doped perovskite manganite oxide nanostructures in the next generation of oxide nanoelectronics. While many fascinating physical properties of rare earth-doped perovskite manganite oxide nanostructures are originated from the interactions among the spin, charge, orbital, and lattice degrees of freedom, whereas there is still a long way to go for obtaining a full understanding of the interaction mechanisms among the spin, charge, orbital, and lattice degrees of freedom. It is expected that in the next years, further progress will be achieved in the experimental and theoretical investigations on rare earth-doped perovskite manganite oxide nanostructures. We believe that this review of the recent advances on rare earth-doped perovskite manganite oxide nanostructures will motivate their future researches and applications of not only in the fields of oxide nanoelectronics, but also in energy and biomedical fields.

## Data Availability

It is a review article that gives a comprehensive overview of the recent progress in the fabrication, structural characterization, physical properties, and functional applications of rare earth-doped perovskite manganite oxide nanostructures.

## Abbreviations

0D: Zero-dimensional
1D: One-dimensional
2D: Two-dimensional
AAO: Anodic aluminium oxide
AFM: Antiferromagnetic
AFM: Atomic force microscopy
CMR: Colossal magnetoresistance
CSD: Chemical solution deposition
CTAB: Cetyltrimethylammonium bromide
CVD: Chemical vapor deposition
EDS: Energy Dispersive X-ray spectroscopy
EELS: Electronic energy loss spectroscopy
EPS: Electronic phase separation
FC: Field cooling
FE-SEM: Field-emission scanning electron microscopy
FIB: Focus ion beam
FM: Ferromagnetic
FTIR: Fourier-transform infrared spectroscopy
HRTEM: High-resolution TEM
LAO: LaAlO_3_
LCMO: La_1-x_Ca_x_MnO_3_
LPCMO: (La_5/8-0.3_Pr_0.3_)Ca_3/8_MnO_3_
LSMO: La_0.7_Sr_0.3_MnO_3_
MBE: Molecular beam epitaxy
MCE: Magnetocaloric effect
M-H: Microwave-hydrothermal
M–I: Metal-insulator
MOCVD: Metalorganic chemical vapor deposition
MR: Magnetoresistance
MRI: Magnetic resonance imaging
MSS: Molten salt synthesis
MTJ: Magnetic tunneling junctions
NGO: NdGaO_3_
PLD: Pulsed laser deposition
PMMA: Polymethylmethacrylate
PVD: Physical vapor deposition
RCP: Relative cooling power
SAED: Selected area electron diffraction
SEBL: Scanning electron beam lithography
SG: Spin glass
SOFCs: Solid oxide fuel cells
SPM: Super-paramagnetic
STEM: Scanning transmission electron microscopy
STO: SrTiO_3_
TCR: Temperature coefficient of resistivity
TEM: Transmission electron microscopy
UHV: Ultra-high vacuum
VOCs: Volatile organic compounds
XPS: X-Ray photoelectron spectroscopy
XRD: X-ray diffraction
XRL: X-ray lithography
YSZ: Yttria-stabilized zirconia
ZFC: Zero-field cooling
